# Supplementary material for: High-throughput characterization of the role of non-B DNA motifs on promoter function
Source: Cell Genom. 2022 Mar 15;2(4):100111. doi: 10.1016/j.xgen.2022.100111 (PMC9105345; doi:10.1016/j.xgen.2022.100111)
Supplement: Document S1. Figures S1–S15 and Tables S1 and S2 [file mmc1.pdf]

Cell Genomics, Volume *2*

Supplemental information

High-throughput characterization of the role of non-B DNA motifs on promoter function

Ilias Georgakopoulos-Soares, Jesus Victorino, Guillermo E. Parada, Vikram Agarwal, Jingjing Zhao, Hei Yuen Wong, Mubarak Ishaq Umar, Orry Elor, Allan Muhwezi, Joon-Yong An, Stephan J. Sanders, Chun Kit Kwok, Fumitaka Inoue, Martin Hemberg, and Nadav Ahituv

Supplemental Information

Table S1, related to figure 4. Selected putative G-quadruplexes for validation experiments. G-runs are marked in bold. Coordinates shown in hg19.

Gene Name	Sequence (5′-3′)	MPRA Coordinates
KCNB1	**GGG**T**GGGG**CCTCCC**GGG**CTCCA**GGGG**	chr20:48099339-48099539
DFF3	**GGGG**C**GGGGG**CCGC**GGG**CTC**GGGGG** CGC**GGGG**	chr14:73360145-73360345
DFF3	**GGGGG**AAGCAGC**GGG**TCCC**GGG**CGT GCT**GGGG**	chr14:73360145-73360345
GALNT9	**GGG**CT**GGGGG**T**GGGGG**CAGCC**GGGG**	chr12:132906730-132906930
AKT1	**GGG**CCGT**GGGG**CTCCCC**GGG**CGCT**GG G**	chr14:105261369-105261569
PRSS27	**GGGG**CGGCAC**GGGG**C**GGGG**CTGCGC C**GGGGG**AA**GGG**	chr16:2827279-2827479
CNOT6	**GGGGG**TAA**GGGGG**C**GGGG**CCT**GGG**	chr5:179921169-179921369
SERTAD 2	**GGGG**AC**GGG**C**GGGG**TAA**GGGGG**	chr2:64978074-64978274
ARF5	**GGGGG**C**GGGG**CCCGGAC**GGGGG**C**GG G**	chr7:127228267-127228467
TNX2	**GGG**CGAAGGC**GGGGG**C**GGGG**C**GGGG**	chr22:36878177-36878377

Table S2, related to figure 5. Selected gene coordinates for the MPRA experiment.
The promoters of ten gene targets were tiled. Marked is the non-B DNA motif that has been analyzed in previous studies. The human genome reference strand is defined as “Reference” and reference reverse complement strand as “Complement”.

Gene Name	Diseases associated	Strand	Coordinates MPRA	TSS position	Non-B DNA Motif	References
C-MYC	Cancer	Reference	chr8:128748165-128748364	chr8:128748315	DR, **G4**, H-DNA, IR	[25], [71], [56]
BCL-2	Cancer	Complement	chr18:60987335-60987534	chr18: 60987360	**G4**, STR	[72], [73]
ADAM-12	Cancer	Complement	chr10:128076900-128077099	chr10:128077024	IR, **Z-DNA**	[18]
C-KIT	Cancer	Reference	chr4:55523900-55524099	chr4:55524084	**G4**, IR, Z-DNA	[74]
FMR1	Fragile X Syndrome	Reference	chrX:146993440-146993639	chrX:146993469	**STR**	[75], [76]
KRAS	Cancer	Complement	chr12:25403860-25404059	chr12:25403870	**G4**, STR	[77], [78]
SNX-12	neurodegenerative diseases	Complement	chrX:70288232-70288431	chrX:70288272	STR, **Z-DNA**	[79], [18]
VEGF-12	Cancer	Reference	chr6:43737771-43737970	chr6:43737921	**G4**, IR, STR	[80]
SRSF-6	Cancer	Reference	chr20:42086418-42086617	chr20:42086568	IR, **Z-DNA**	[18]
ALOX-5	Cancer	Reference	chr10:45869511-45869710	chr10:45869661	**G4,** STR	[81]

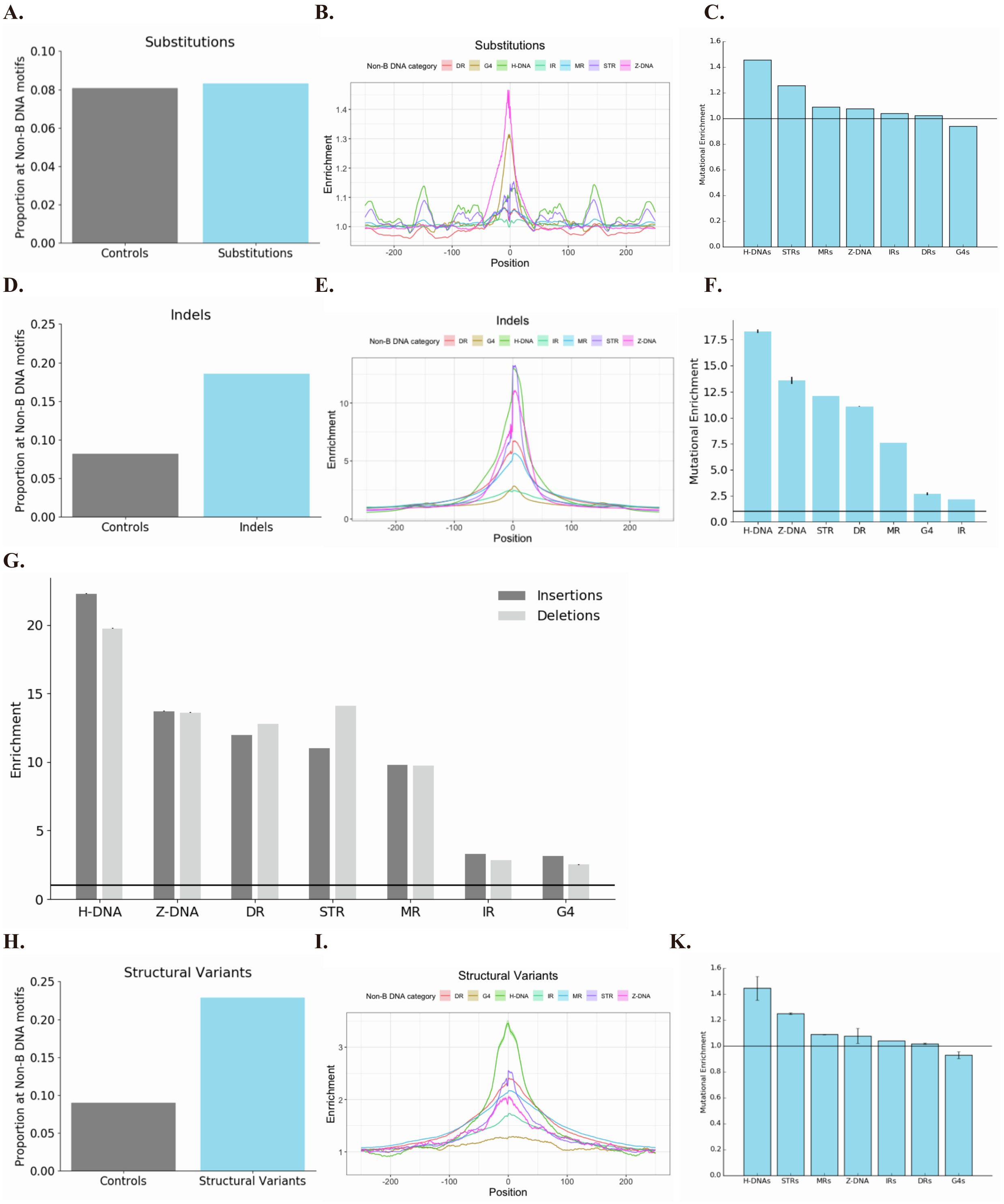

Figure S1, related to figure 1. Mutational density is locally increased at non-B DNA motifs.

a. Distribution of non-B DNA motifs relative to SNP positioning, uncorrected for trinucleotide context.

b. Proportion of SNP variants and simulated controls overlapping non-B DNA motifs. Mann-Whitney U test p-value <0.0001.

c. Enrichment of SNPs at non-B DNA motifs, relative to controls. Error bars indicate standard error from bootstrapping.

d. Distribution of non-B DNA motifs relative to indel variants positioning, uncorrected for trinucleotide context.

e. Proportion of indel variants and simulated controls overlapping non-B DNA motifs. Mann-Whitney U test p-value <0.0001.

f. Enrichment of indel variants at non-B DNA motifs, relative to controls. Error bars indicate standard error from bootstrapping.

g. Proportion of insertions and deletions overlapping each non-B DNA motif category. Error bars indicate standard error from bootstrapping.

h. Proportion of structural variants and simulated controls overlapping non-B DNA motifs. Mann-Whitney U test p-value <0.0001.

i. Proportion of structural variant breakpoints and simulated controls overlapping non-B DNA motifs. Mann-Whitney U test p-value <0.0001.

k. Enrichment of structural variant breakpoints at non-B DNA motifs, relative to controls. Error bars indicate standard error from bootstrapping. For certain barplots in this figure the standard error is smaller than the resolution of the image.

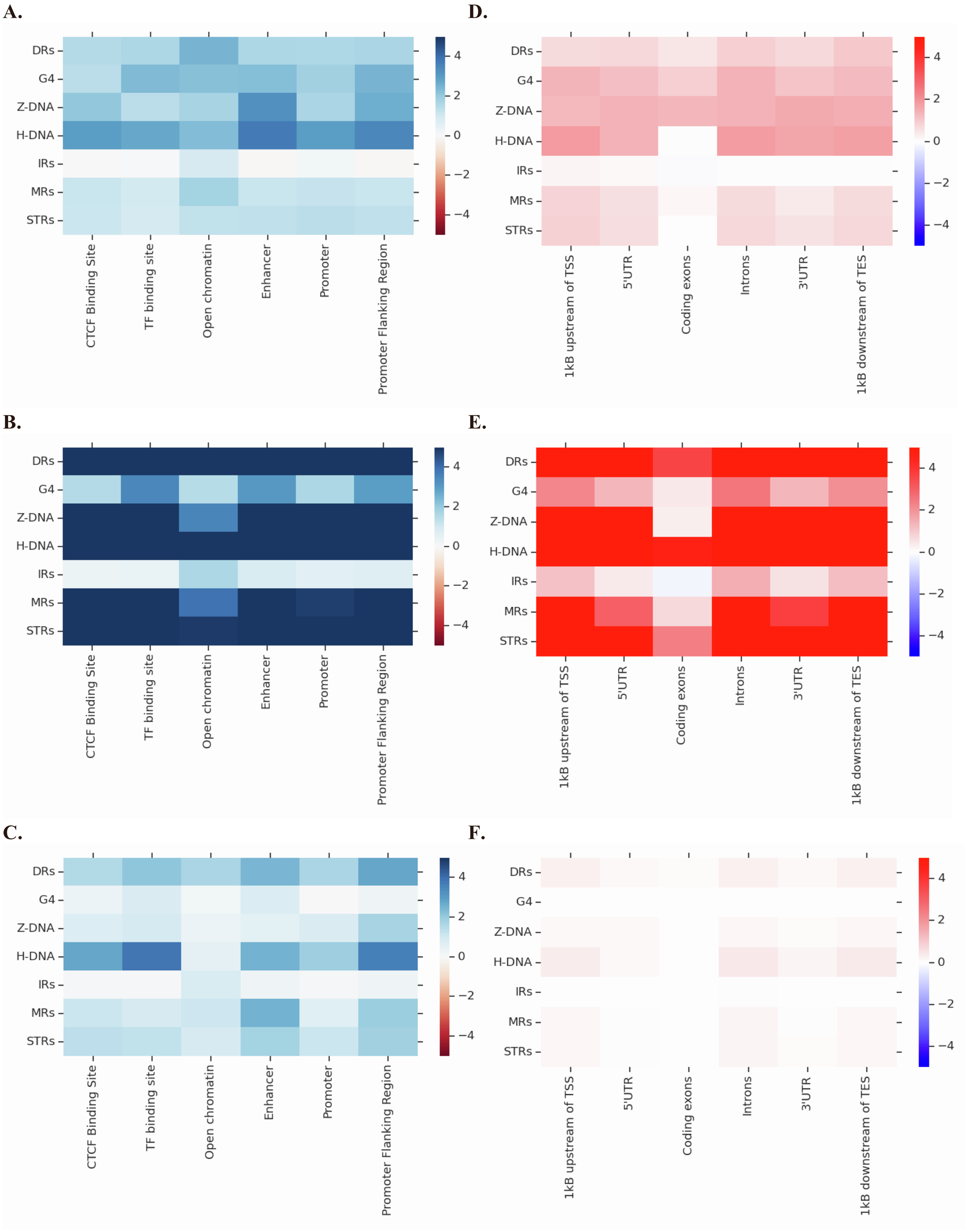

Figure S2, related to figure 1. Mutational distribution at non-B DNA motifs across regulatory elements and genic compartments.

a. Z-scores of the relationship between SNP variant frequency at Ensembl regulatory elements and non-B DNA motifs.
b. Z-scores of the relationship between indel variant frequency at Ensembl regulatory elements and non-B DNA motifs.
c. Z-scores of the relationship between structural variant frequency at Ensembl regulatory elements and non-B DNA motifs.
d. Z-scores of the relationship between SNP variant frequency at genic regions and non-B DNA motifs.
e. Z-scores of the relationship between indel variant frequency at genic regions and non-B DNA motifs.
f. Z-scores of the relationship between structural variant frequency at genic regions and non-B DNA motifs.

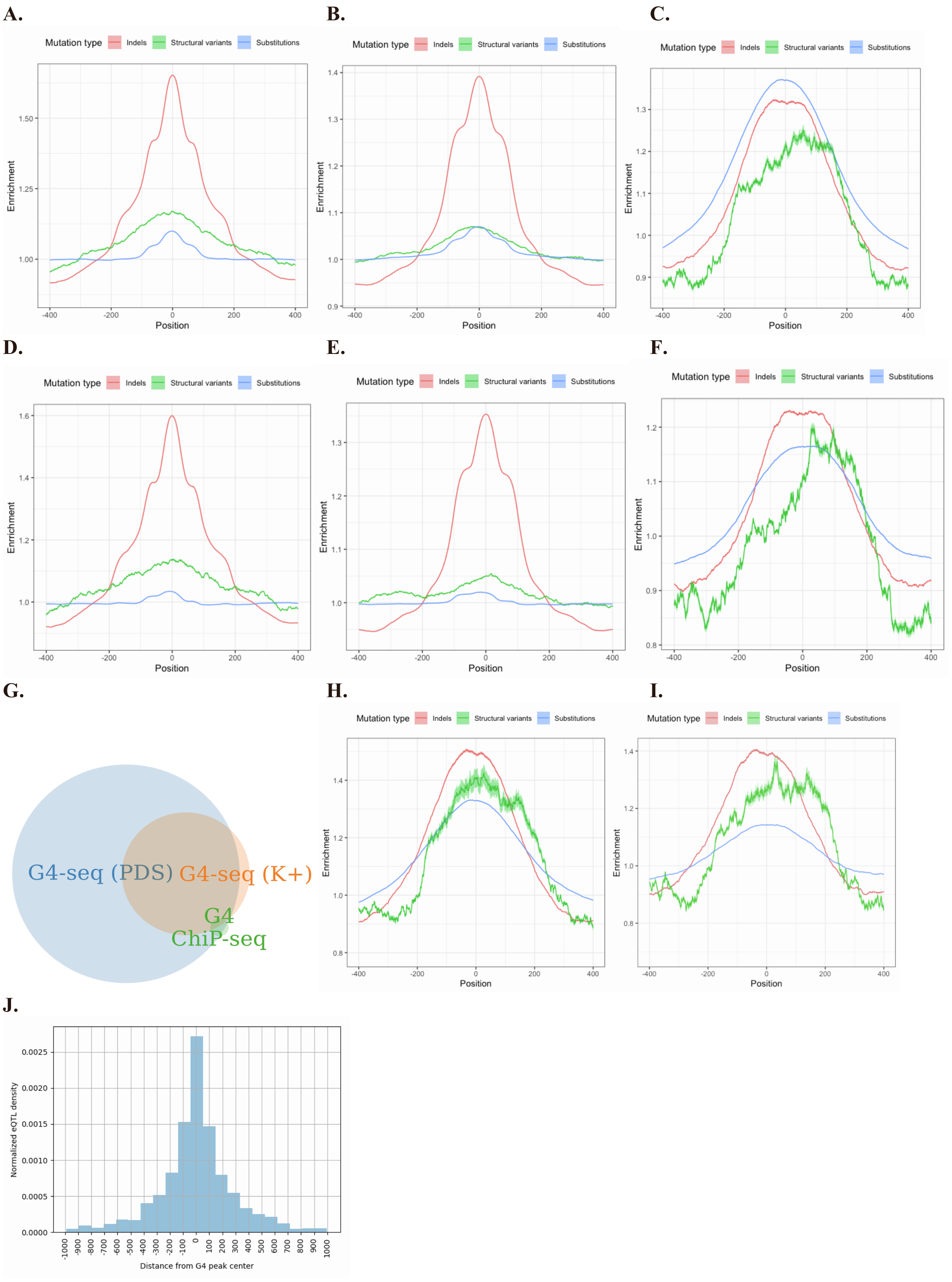
A.
B.
C.
D.
E.
F.
Mutation type
Indels
Structural variants
Substitutions
Enrichment
Position
G.
G4-seq (PDS)
G4-seq (K+)
G4
ChiP-seq
H.
I.
J.
Normalized eQTL density
Distance from G4 peak center

Figure S3, related to figure 1. G4 sequences identified from G4-seq and G4 ChIP-seq experiments are enriched for population variants.

a-c. Enrichment of mutations overlapping G4 sites derived from G4-seq and ChIP-seq experiments for **a.** K^+ treatment and **b.** PDS treatment, **c.** ChIP-seq experiment.

d-f Enrichment of mutations overlapping G4 sites correcting for trinucleotide context and mutation location, for G4-seq and ChIP-seq experiments for **d.** K^+ treatment and **e.** PDS treatment, **f.** G4 ChIP-seq experiment.

g. Venn diagram displaying the intersection between the two G4-seq experiments with PDS and K^+ treatments and the G4 ChIP-seq peaks.

h. Enrichment of variants from the center of G4 ChIP-seq peaks overlapping G4-seq peaks from PDS and K^+ treatments.

i. Enrichment of mutations overlapping G4 sites for SNPs, indels and structural variants at G4 peaks from G4 antibody treatment derived G4 sites.

j. eQTL density at the center of G4 ChIP-seq peaks overlapping G4-seq peaks from PDS and K^+ treatments.

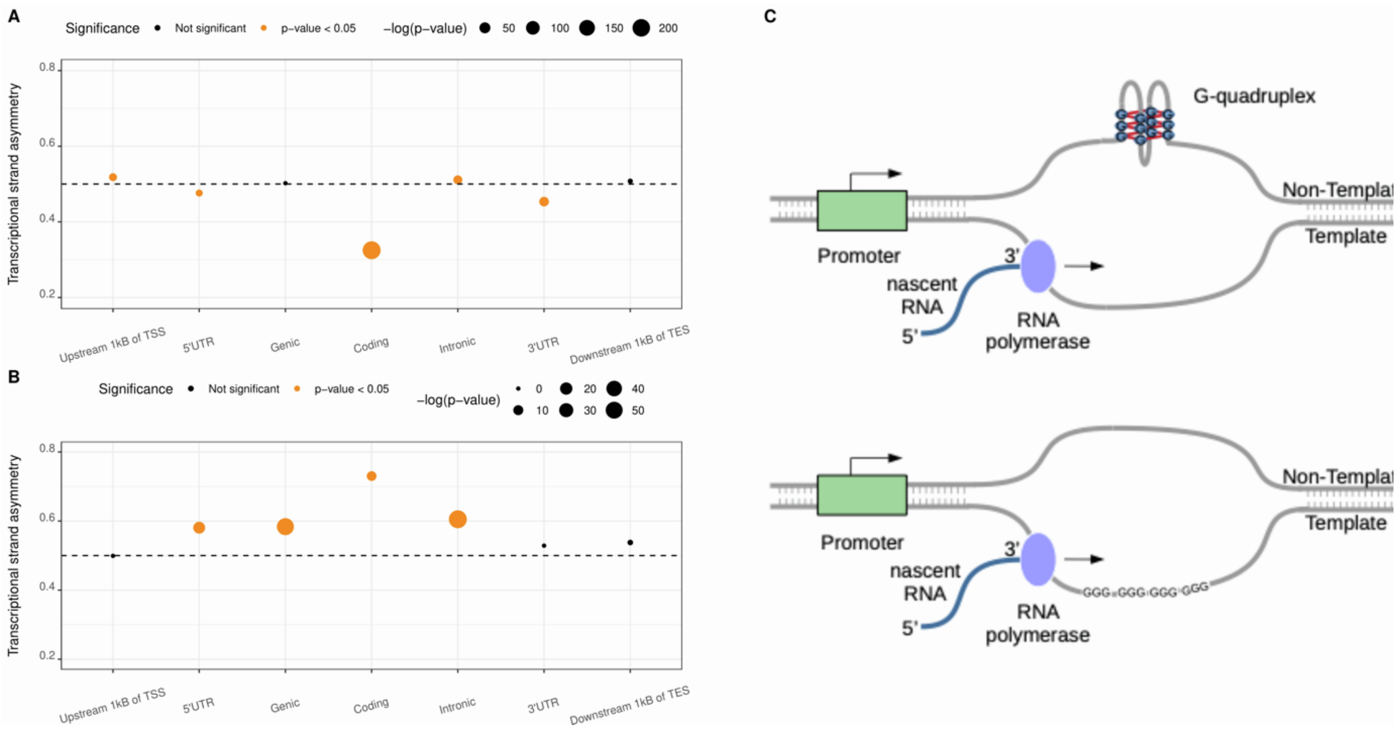

Figure S4, related to figure 1. G4 formation is influenced by its transcriptional orientation.
a. G4 motif strand bias between template and non-template strands.
b. Strand asymmetry of antibody-bound G4s across genic regions correcting for the background strand asymmetry of all the G4 motifs at each of the genic regions. Error bars represent standard deviation from bootstrapping. Statistical significance of strand asymmetry for antibody-bound G4s across genic regions correcting for the background strand asymmetry of all the G4 motifs at each of the genic regions. P-values are derived from binomial tests with Bonferroni correction.
c. G4s in vivo form preferentially at the non-template strand relative to the template strand, in which they could impede the RNA polymerase progression. Schematic representation of preferential G4 formation at the non-template strand (top) over the template strand (bottom) during transcription elongation.

A.

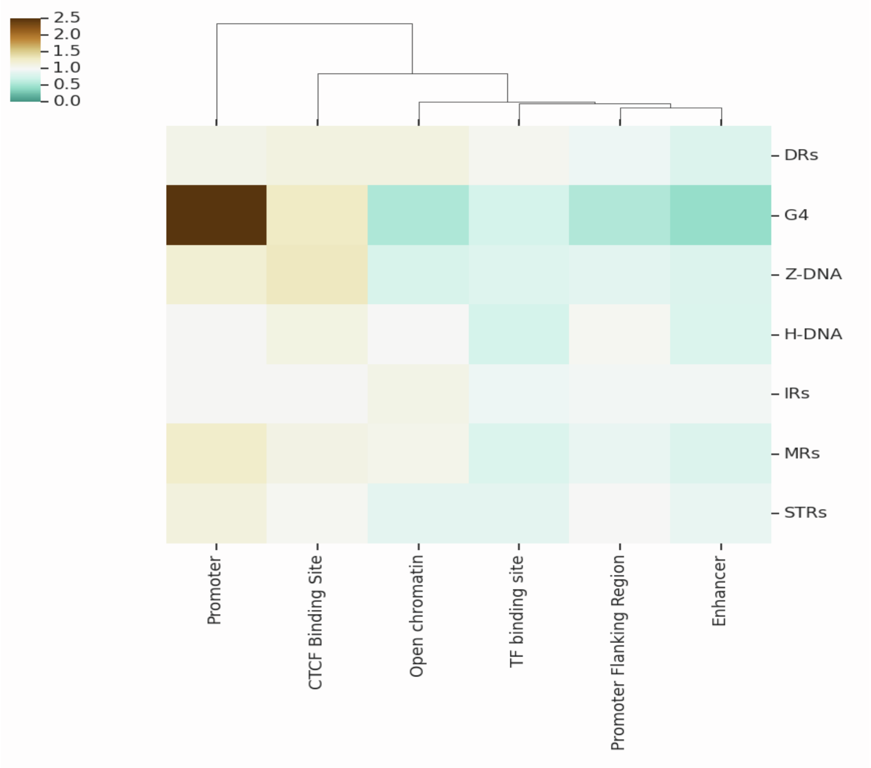

2.5
2.0
1.5
1.0
0.5
0.0
DRs
G4
Z-DNA
H-DNA
IRs
MRs
STRs
Promoter
CTCF Binding Site
Open chromatin
TF binding site
Promoter Flanking Region
Enhancer

B.

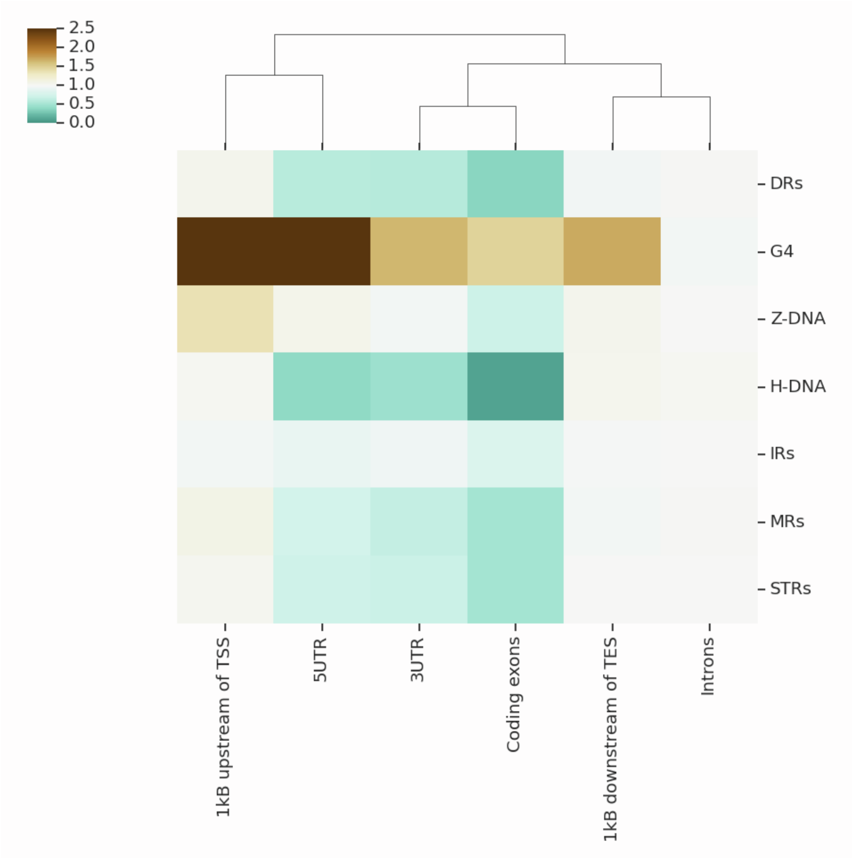

2.5
2.0
1.5
1.0
0.5
0.0
DRs
G4
Z-DNA
H-DNA
IRs
MRs
STRs
1kB upstream of TSS
5UTR
3UTR
Coding exons
1kB downstream of TES
Introns

Figure S5, related to figure 2. Non-B DNA motifs at regulatory regions and genes.
a. Enrichment of non-B DNA motifs across regulatory regions.
b. Enrichment of non-B DNA motifs across genic sub-compartments.

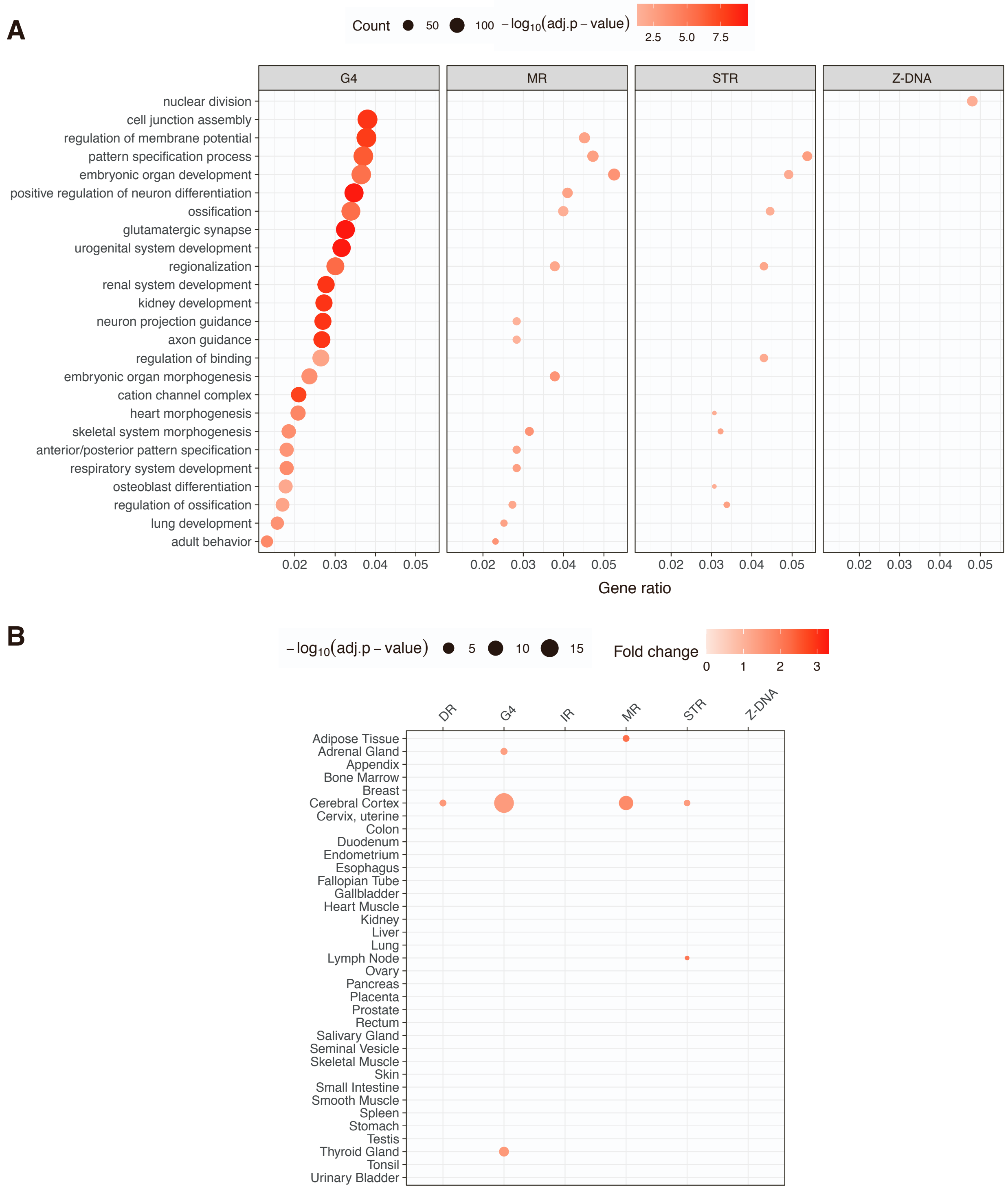

Figure S6, related to figure 2. Relationship between non-B DNA motifs at promoters, gene categories and tissue expression.

a. Gene ontology analysis of non-B DNA motifs found in gene promoters. For each non-B DNA motif up to the ten GO categories with highest gene ratio are shown.

b. Tissue-specific gene enrichment analysis for genes with non-B DNA motifs in their promoters.

A.

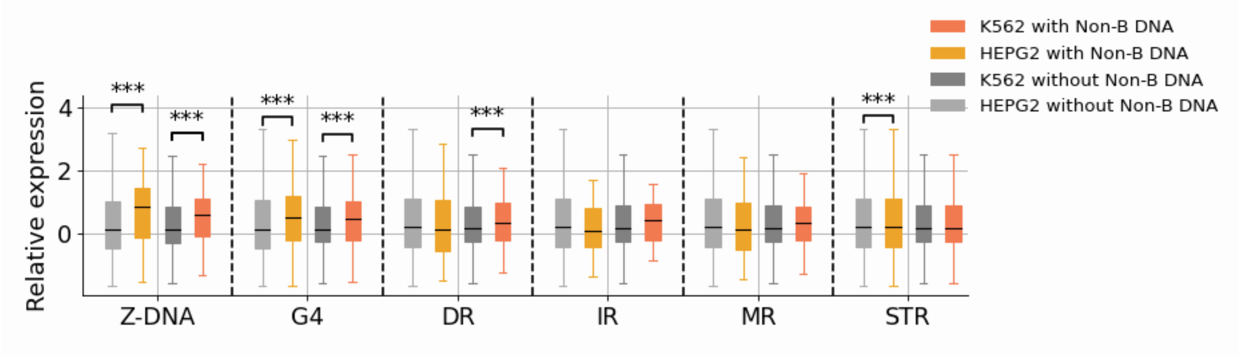

B.

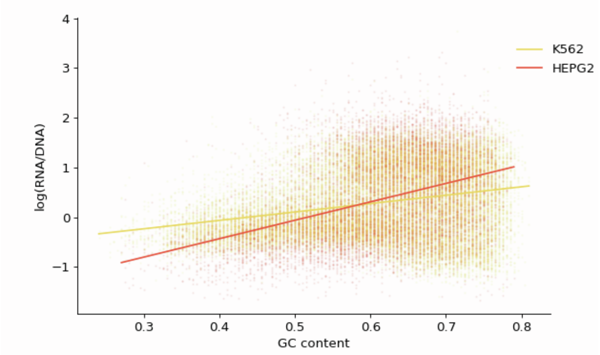

C.

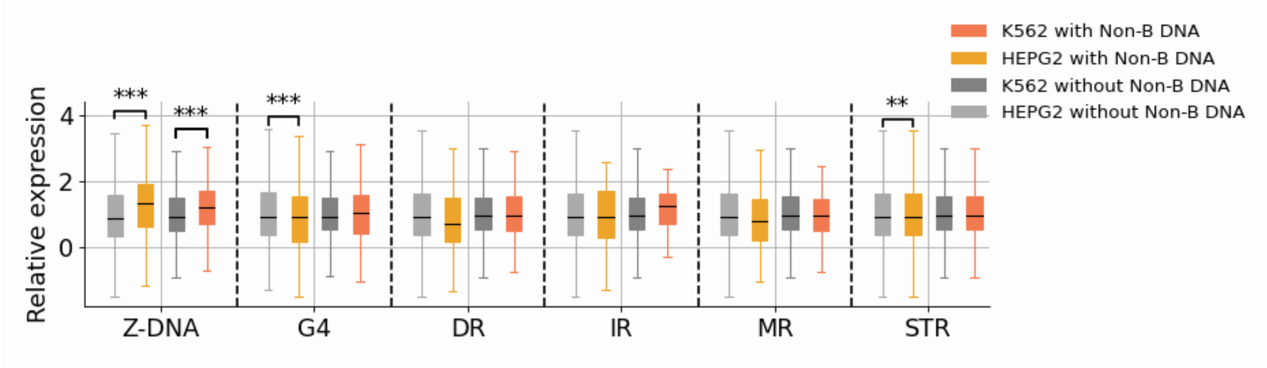

D.

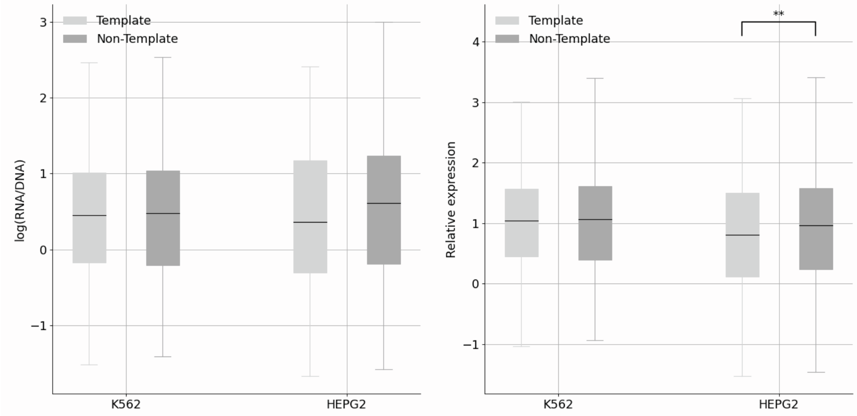

Figure S7, related to figure 3. Association between presence of different non-B DNA motifs and expression.

a. Expression of sequences with and without each non-B DNA motif are shown.
b. Fourth order polynomial model fitting the GC content and expression levels in HEPG2 and K562 cell lines.
c. Expression of sequences with and without each non-B DNA motif before and after GC content correction.
d. Expression of G4s found at the template and non-template orientations.
In figure panels adjusted p-values from t-tests with Bonferroni correction are displayed as * for p-value<0.05, ** for p-value<0.01 and *** for p-value<0.001.

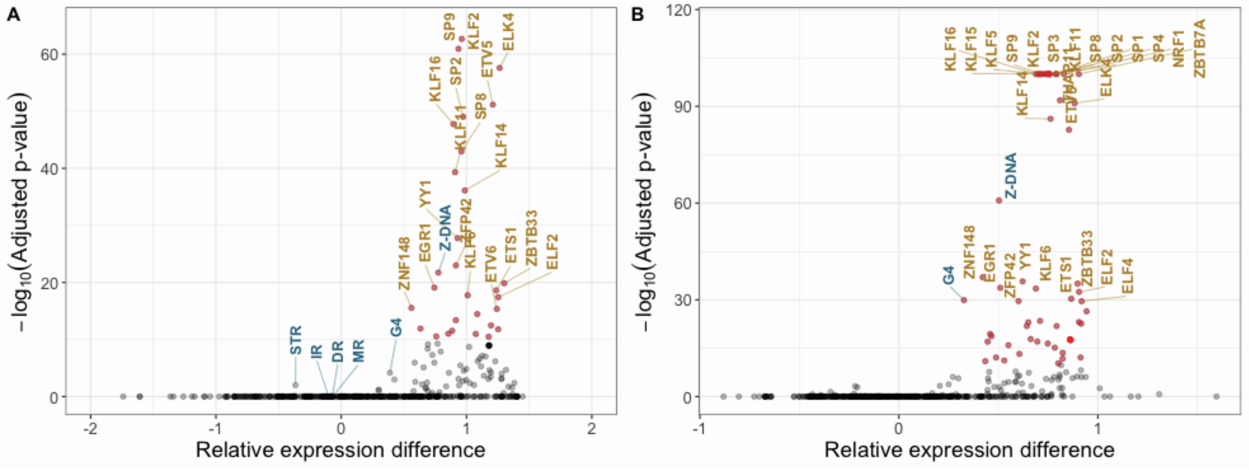

Figure S8, related to figure 3. Relative expression difference between the median expression for sequences with and without non-B DNA motifs and transcription factor binding sites. Results displayed in **A.** HepG2 and **B.** K562 cell lines, without performing GC content correction. Statistical significance is estimated with t-tests and Bonferroni correction.

A.

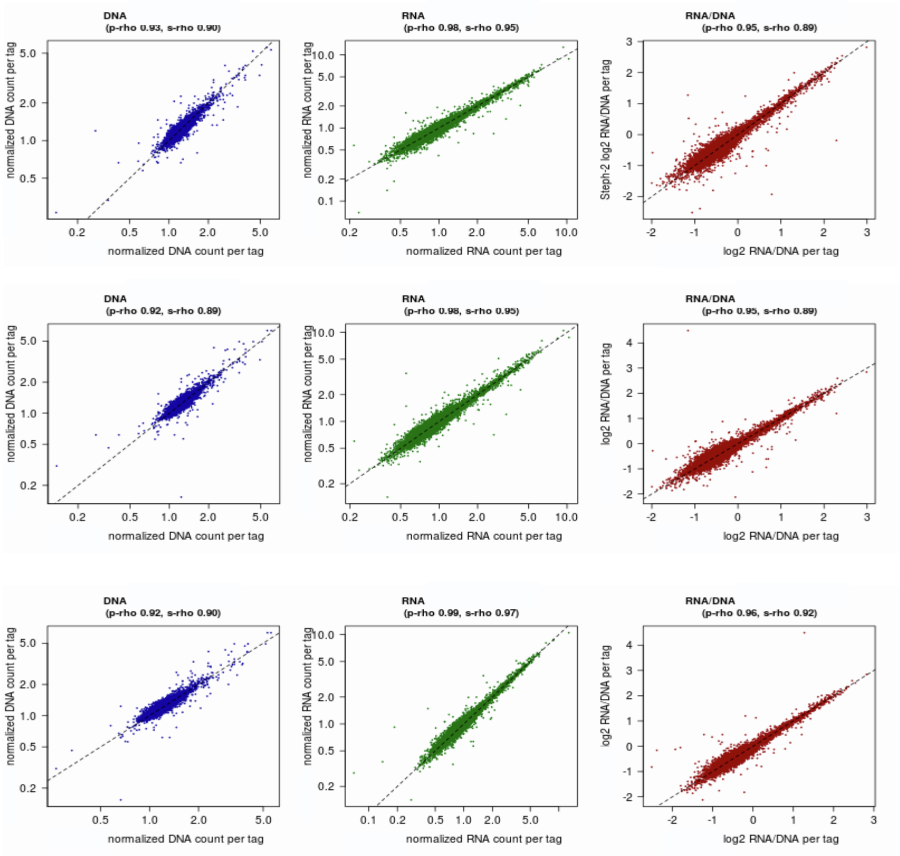
DNA
(p-rho 0.93, s-rho 0.90)
normalized DNA count per tag
normalized DNA count per tag
RNA
(p-rho 0.98, s-rho 0.95)
normalized RNA count per tag
normalized RNA count per tag
RNA/DNA
(p-rho 0.95, s-rho 0.89)
Steph-2 log2 RNA/DNA per tag
log2 RNA/DNA per tag
DNA
(p-rho 0.92, s-rho 0.89)
RNA
(p-rho 0.98, s-rho 0.95)
RNA/DNA
(p-rho 0.95, s-rho 0.89)
log2 RNA/DNA per tag
DNA
(p-rho 0.92, s-rho 0.90)
RNA
(p-rho 0.99, s-rho 0.97)
RNA/DNA
(p-rho 0.96, s-rho 0.92)

B.

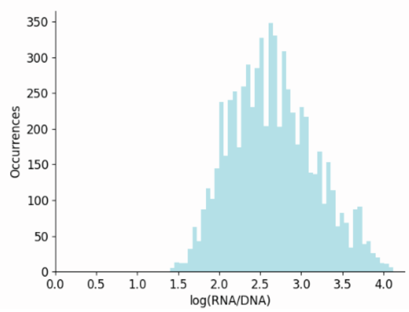
Occurrences
log(RNA/DNA)

C.

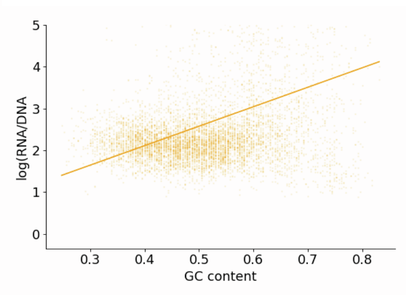
log(RNA/DNA)
GC content

D.

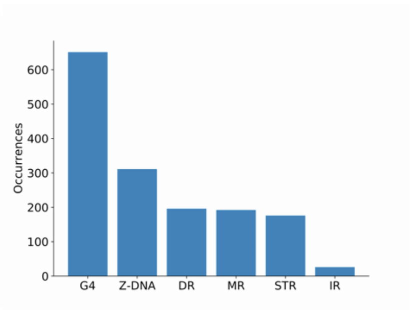
Occurrences
G4
Z-DNA
DR
MR
STR
IR

E.

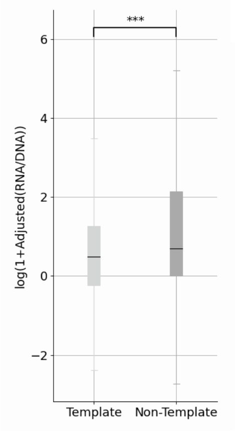
***
log(1+Adjusted(RNA/DNA))
Template
Non-Template

F.

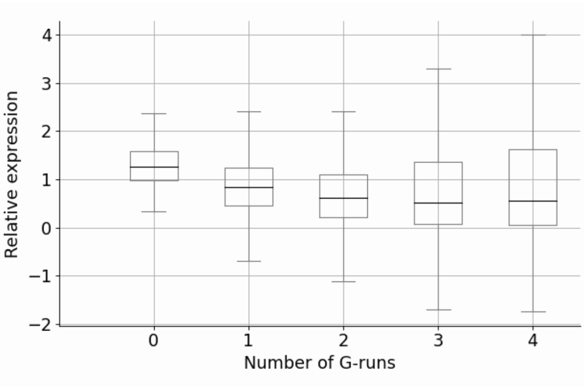
Relative expression
Number of G-runs

Figure S9, related to figure 4. Evaluation of non-B DNA motif regulatory roles using a neuronal MPRA.

a. Quality control between replicates of the MPRA experiment for the NPC cell line.

b. RNA/ DNA ratio for the autism dataset.

c. Scatter plot displaying association between expression levels and GC-content (Pearson correlation between GC content and expression levels: 0.32).

d. Number of occurrences of each non-B DNA motif category across the MPRA sequences

e. The orientation of G4s is significantly associated with expression (p-value<0.001, t-test).

f. Consecutive G-runs are associated with decreased expression levels.

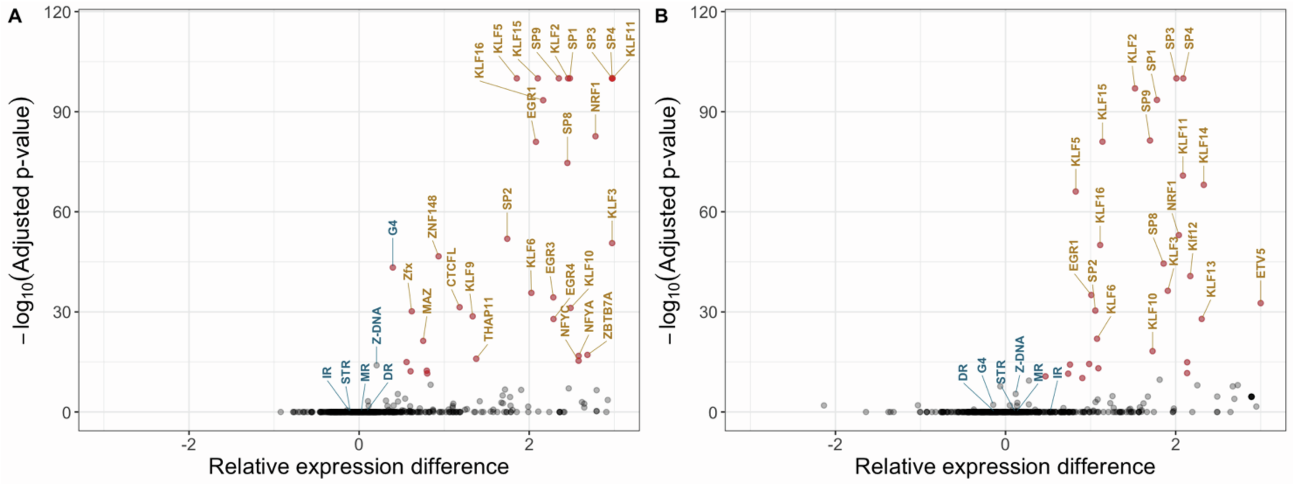

Figure S10, related to figure 4. Relative expression difference between the median expression for sequences with and without non-B DNA motifs and transcription factor binding sites in NPC cell lines.

a. Results displayed without performing GC content correction,

b. Results displayed after performing GC content correction.

Statistical significance is estimated with t-tests tests and Bonferroni correction.

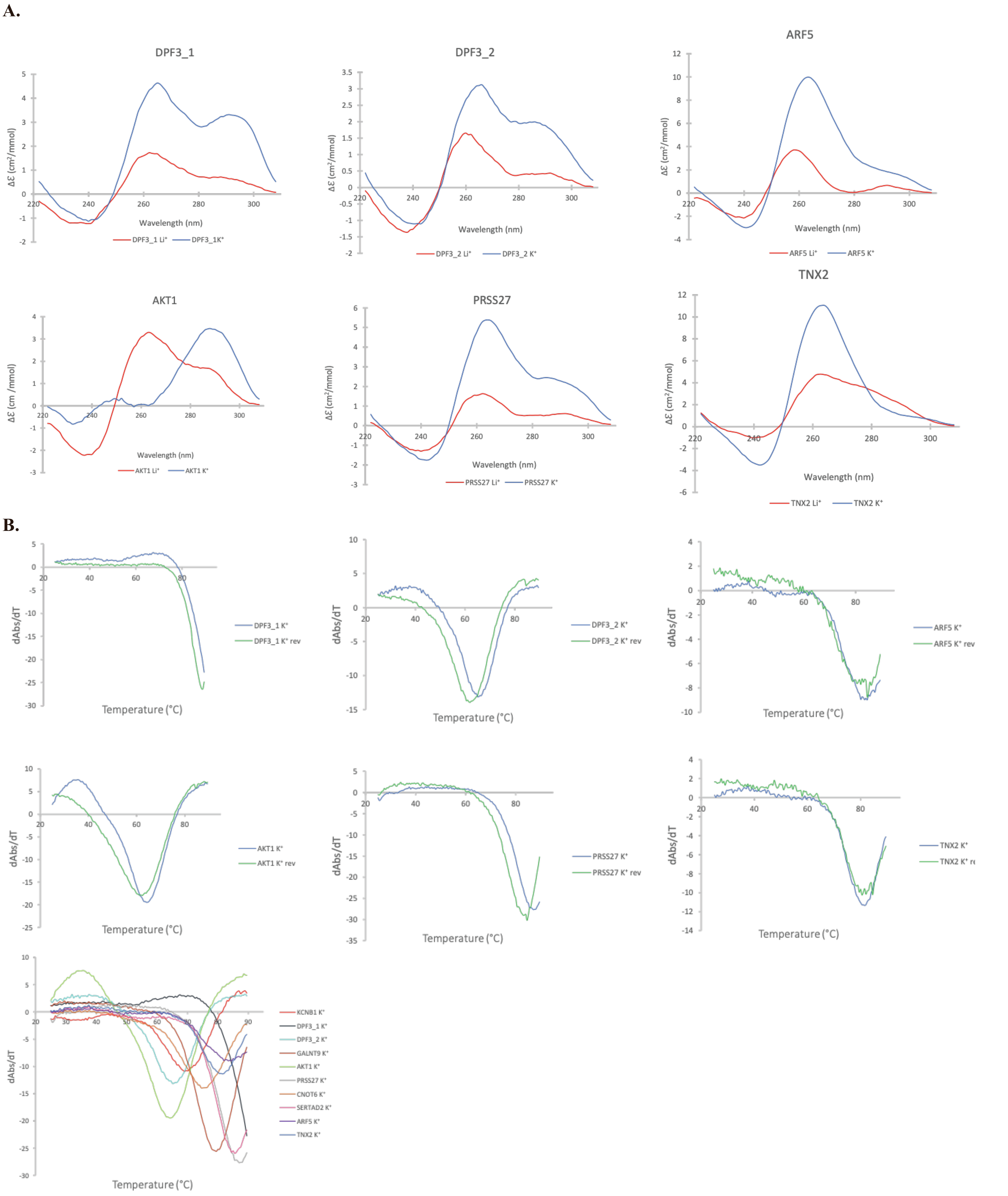
A.
DPF3_1
DPF3_2
ARF5
AKT1
PRSS27
TNX2
Wavelength (nm)
DPF3_1 Li⁺
DPF3_1K⁺
DPF3_2 Li⁺
DPF3_2 K⁺
ARF5 Li⁺
ARF5 K⁺
AKT1 Li⁺
AKT1 K⁺
PRSS27 Li⁺
PRSS27 K⁺
TNX2 Li⁺
TNX2 K⁺
B.
dAbs/dT
Temperature (°C)
DPF3_1 K⁺
DPF3_1 K⁺ rev
DPF3_2 K⁺
DPF3_2 K⁺ rev
ARF5 K⁺
ARF5 K⁺ rev
AKT1 K⁺
AKT1 K⁺ rev
PRSS27 K⁺
PRSS27 K⁺ rev
TNX2 K⁺
KCNB1 K⁺
DPF3_1 K⁺
DPF3_2 K⁺
GALNT9 K⁺
AKT1 K⁺
PRSS27 K⁺
CNOT6 K⁺
SERTAD2 K⁺
ARF5 K⁺
TNX2 K⁺

Figure S11, related to figure 4. Experimental validation of G4 formation potential for selected sequences.

a. Circular dichroism (CD) spectra of the candidate targets for G4 formation potential in presence of two cations. The monovalent ion-dependent nature (G4 stabilized in K^+ but not in Li^+) indicate the formation of DNA G4s. b. UV melting profiles of the G4 candidates in presence of K^+.

b. The reverse melting profile (K^+rev) is also showed and matched well with the forward melting profile (K^+). Hyporchromic shift at 295nm is a hallmark for G4 formation, which can be transformed to negative peak in derivative plot (dAbs/dT) for G4 stability analysis. The melting temperature (Tm) of a G4 can be identified at the maximum negative value.

A.

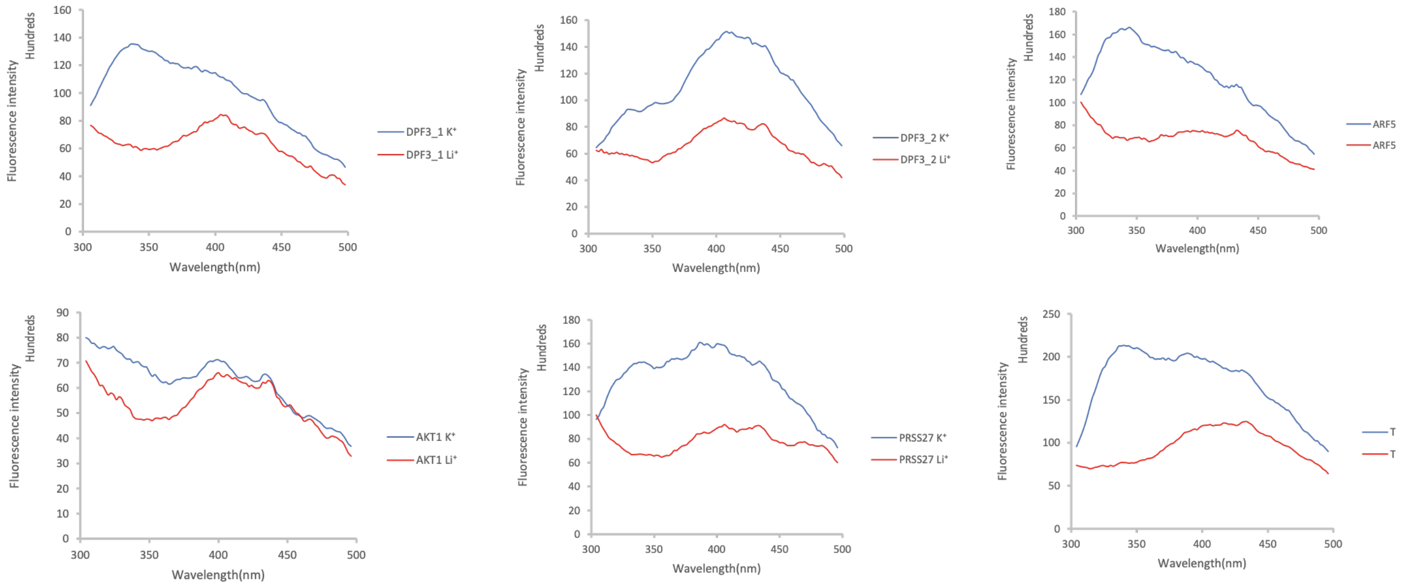

B.

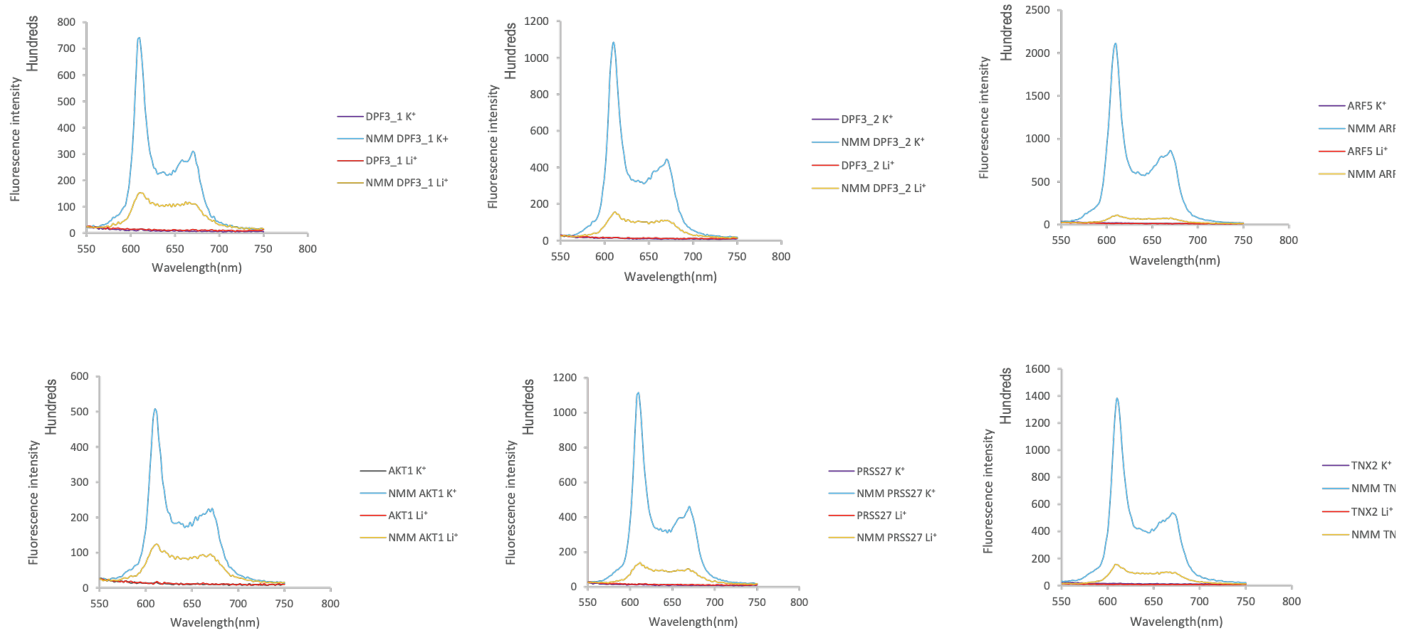

Figure S12, related to figure 4. Validation of G4 containing oligonucleotides using fluorescence based methods.

a. Intrinsic fluorescence of six candidate DNA oligonucleotides under Li^+ or K^+ conditions. The intrinsic fluorescence of G4s was increased when replacing Li^+ with K^+, highlighting the formation of DNA G4s.

b. Fluorescence emission associated with NMM ligand binding to G4 candidates in the presence of Li^+ or K^+ ions. In the absence of NMM ligand, no fluorescence was observed at ~610 nm. Upon NMM addition, weak fluorescence was observed under Li^+, which was substantially enhanced when substituted with K^+, supporting the formation of G4 which allows recognition of NMM and enhances its fluorescence.

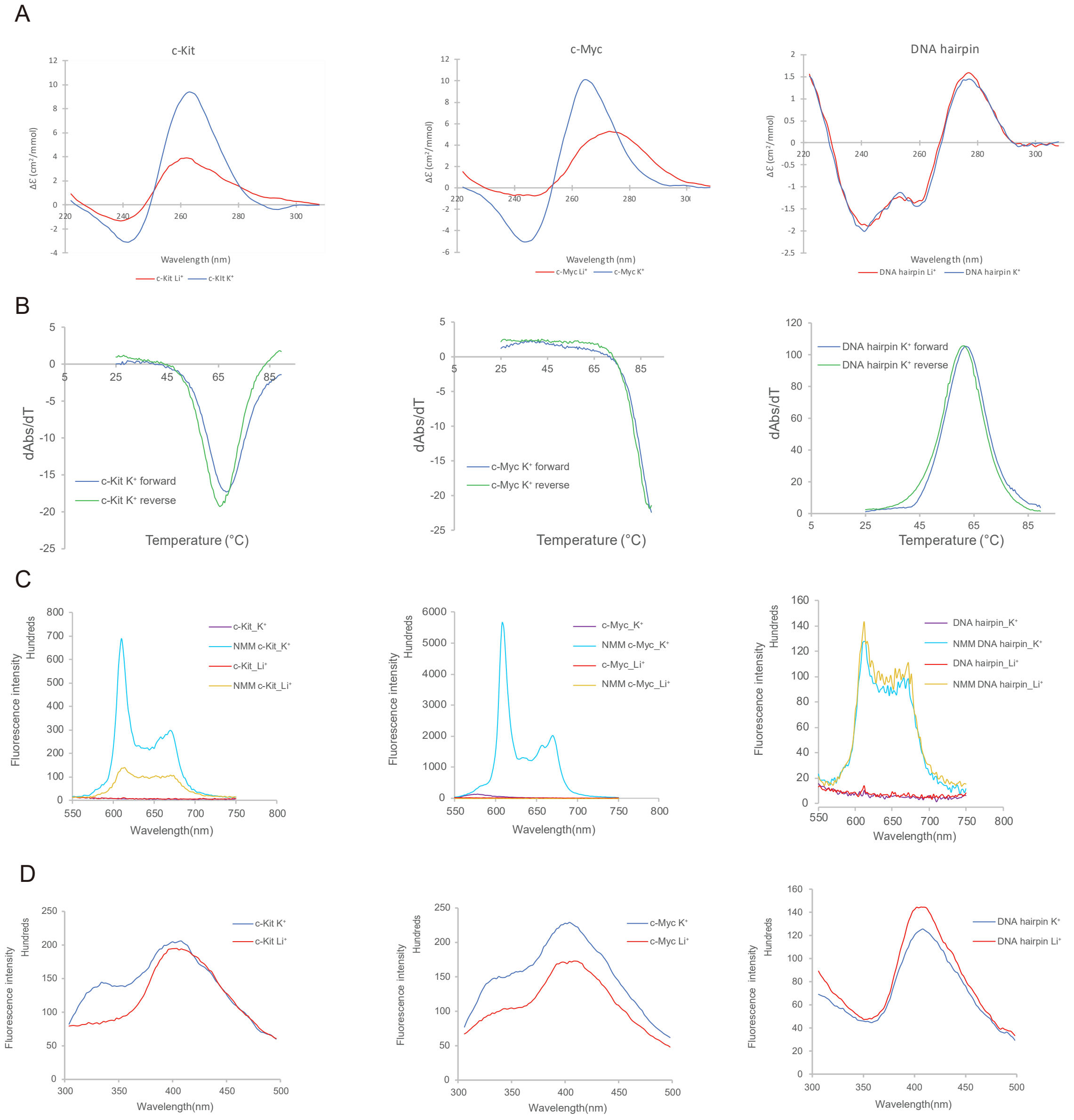

Figure S13, related to figure 4. Validation experiments performed with positive and negative control sequences.

a. Circular dichroism (CD) spectra of the candidate targets for G4 formation potential in presence of two cations. The monovalent ion-dependent nature (G4 stabilized in K^+ but not in Li^+) indicates the formation of DNA G4s, but not in DNA hairpin, the B-DNA motif.

b. UV melting profiles of the G4 candidates in presence of K^+. The reverse melting profile (K^+rev) is also shown and matched well with the forward melting profile (K^+). Hypochromic shift at 295nm is a hallmark for G4 formation, which can be transformed to a negative peak in the derivative plot (dAbs/dT) for G4 stability analysis. The melting temperature (Tm) of a G4 can be identified at the maximum negative value and the B-DNA motif showed a positive value at 260nm instead.

c. Fluorescence emission associated with NMM ligand binding to two G4-DNA and one B-DNA candidates in the presence of Li^+ or K^+ ions. In the absence of NMM ligand, no fluorescence was observed at ~610 nm. Upon NMM addition for two G4-DNAs, weak fluorescence was observed

under Li^+, which was substantially enhanced when substituted with K^+, supporting the formation of G4 which allows recognition of NMM and enhances its fluorescence. However, upon NMM addition for B-DNA, the fluorescence did not show a significant increase in the presence of Li^+ or K^+ ions which indicate no G4 formation for B-DNA with NMM.

d. Intrinsic fluorescence of two G4-DNA and one B-DNA candidates under Li^+ or K^+ conditions. The intrinsic fluorescence of G4s was increased when replacing Li^+ with K^+, highlighting the formation of DNA G4s but not in the B-DNA motif.

A.

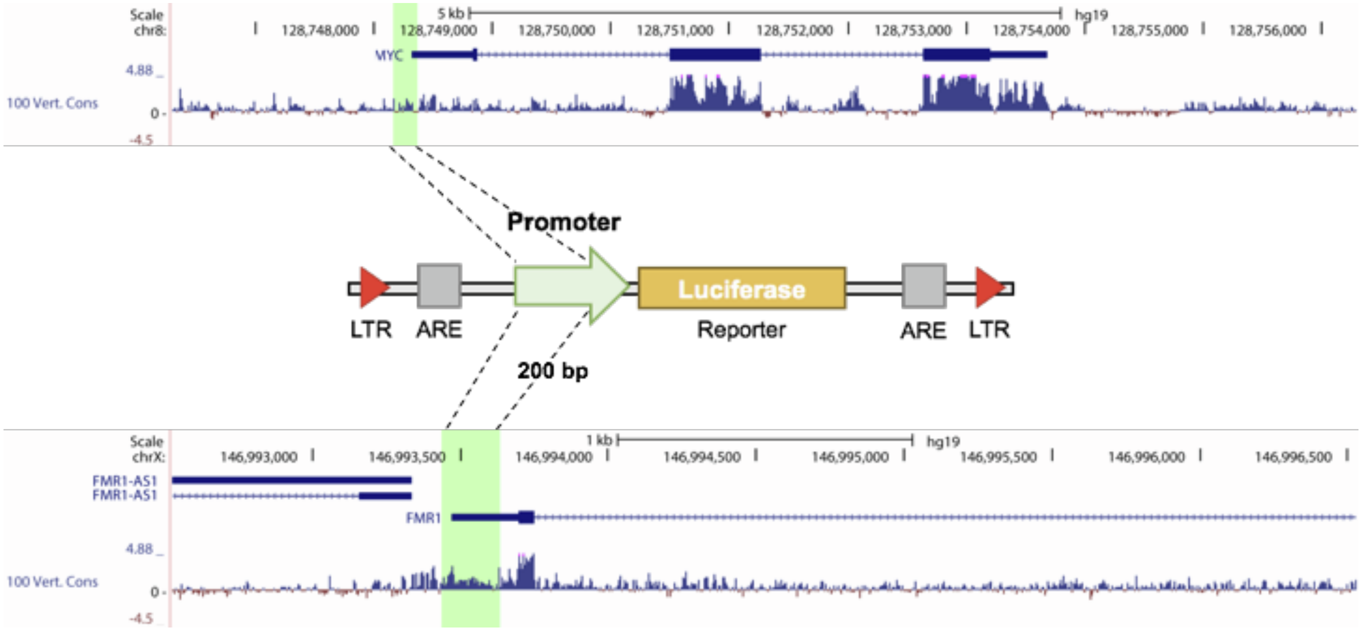

B.

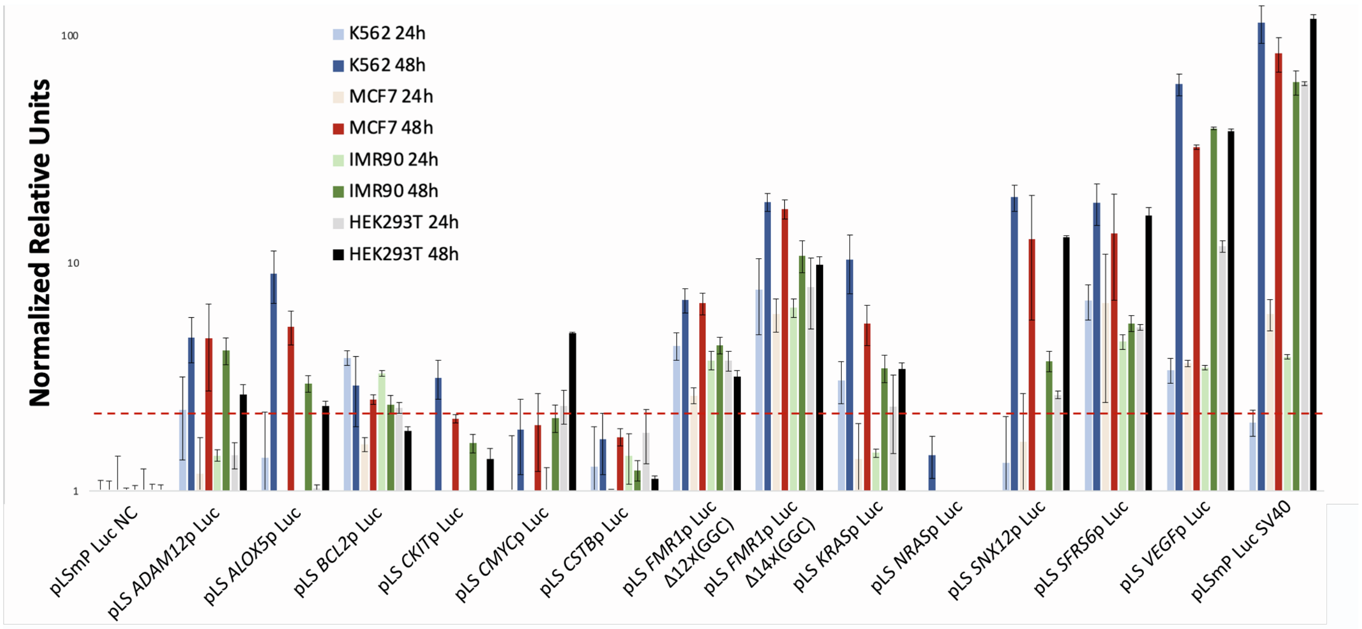

Figure S14, related to figure 5. Assessment of the activity of 12 disease-relevant gene promoters with non-B DNA motifs.

a. Schematic representation of the experimental design behind promoter activity assessment. In this example 200 bp near the TSS of the genes C-MYC and FMR1 are cloned right before a luciferase gene in a promoter-less lentiviral vector.

b. Luciferase assay in 4 different cell lines during 2 time points relative to a negative control (NC). Candidate promoters included two different constructs for the *FMR1* gene promoter, and a positive control (SV40). Results are normalized to the negative control. Red dashed line indicates the 2-fold threshold over the negative control.

A.

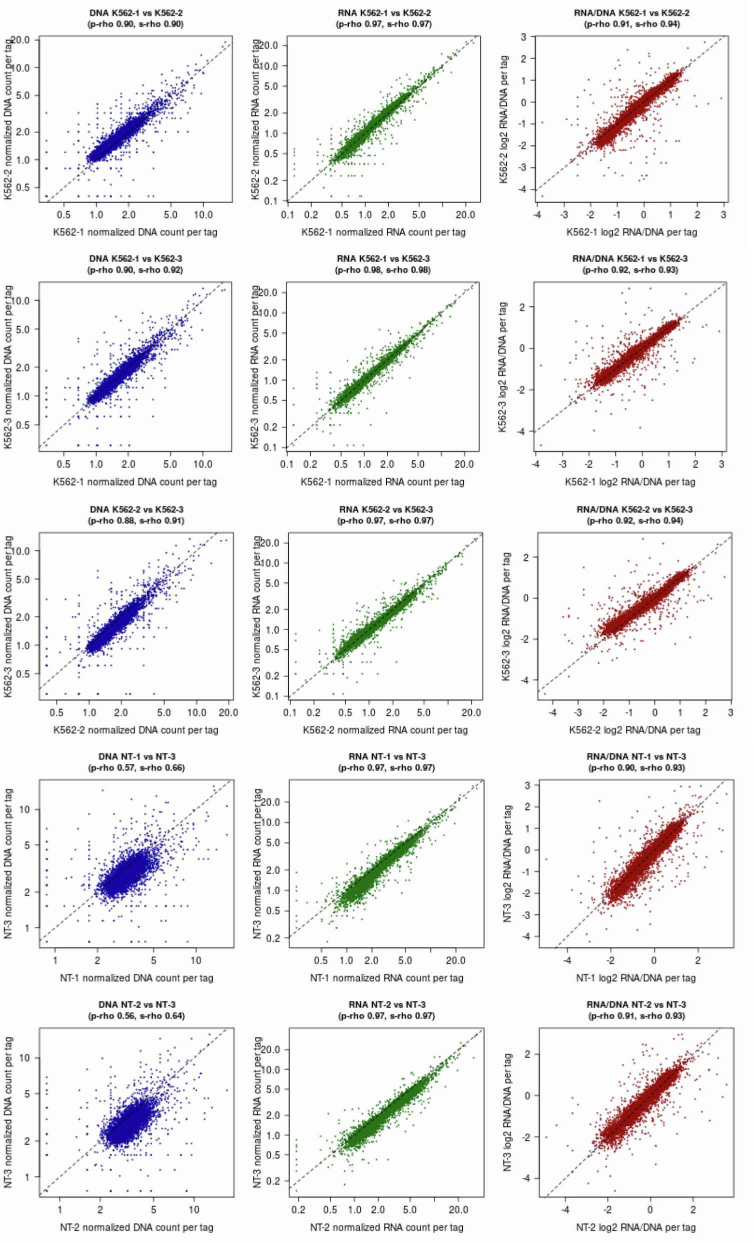

B.

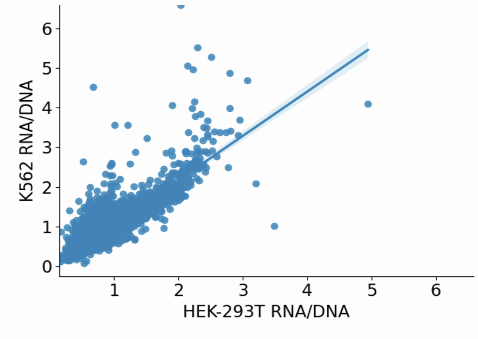

C.

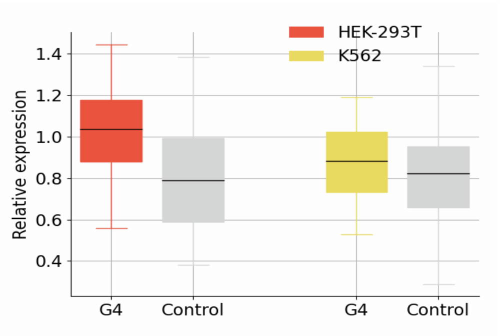

Figure S15, related to figure 5. MPRA quality control evaluation.

a. Quality control between replicates of the MPRA experiment for K562 and HEK-293T cell lines.

b. Comparison of expression levels of sequences between the two cell lines (Pearson r=0.87).
c. Mutations that disrupt G-quadruplexes relative to the original sequences in HEK-293T and K562 cell lines.
