# Supplementary material for: High-throughput characterization of the role of non-B DNA motifs on promoter function
Source: Cell Genom. 2022 Mar 15;2(4):100111. doi: 10.1016/j.xgen.2022.100111 (PMC9105345; doi:10.1016/j.xgen.2022.100111)
Supplement: Document S3. Article plus supplemental information [file mmc3.pdf]

Cell Genomics

Article

High-throughput characterization of the role of non-B DNA motifs on promoter function

Graphical abstract

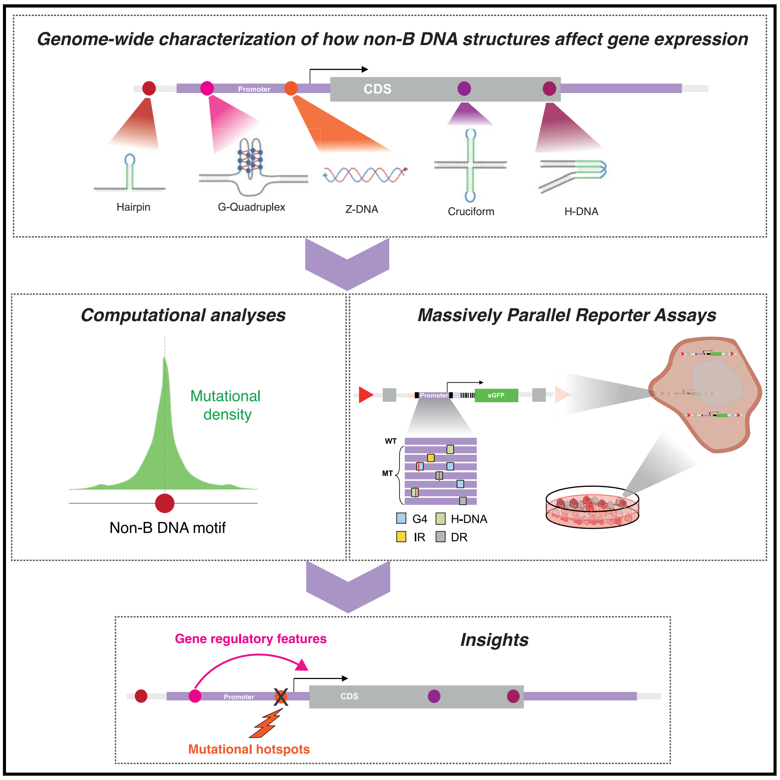

Highlights

- Excess of germline variants at non-B DNA motif loci
- Massively parallel reporter assays measure the impact of non-B DNA motifs on expression
- Z-DNA significantly affects promoter activity across cell types and experiments
- The orientation of G-quadruplexes influences their formation and promoter activity

Authors

Ilias Georgakopoulos-Soares, Jesus Victorino, Guillermo E. Parada, ..., Fumitaka Inoue, Martin Hemberg, Nadav Ahituv

Correspondence

mhemberg@bwh.harvard.edu (M.H.), nadav.ahituv@ucsf.edu (N.A.)

In brief

Georgakopoulos-Soares et al. performed computational analyses of germline mutations and identified increased mutability at non-B DNA motifs. The contribution of non-B DNA motifs on gene expression was investigated using massively parallel reporter assays, identifying Z-DNA as a positive regulator of gene expression and finding that the orientation of G-quadruplexes influences promoter activity.

Georgakopoulos-Soares et al., 2022, Cell Genomics 2, 100111
April 13, 2022 © 2022 The Author(s).
https://doi.org/10.1016/j.xgen.2022.100111

Cell Genomics

CellPress
OPEN ACCESS

Article

High-throughput characterization of the role of non-B DNA motifs on promoter function

Ilias Georgakopoulos-Soares,[1,2] Jesus Victorino,[3,4,14] Guillermo E. Parada,[5,6,14] Vikram Agarwal,[7] Jingjing Zhao,[1,2] Hei Yuen Wong,[8] Mubarak Ishaq Umar,[8] Orry Elor,[1] Allan Muhwezi,[5] Joon-Yong An,[9,10] Stephan J. Sanders,[2,9] Chun Kit Kwok,[8,11] Fumitaka Inoue,[1,2,12] Martin Hemberg,[5,6,13,*] and Nadav Ahituv[1,2,15,*]
[1]Department of Bioengineering and Therapeutic Sciences, University of California San Francisco, San Francisco, CA, USA
[2]Institute for Human Genetics, University of California San Francisco, San Francisco, CA, USA
[3]Centro Nacional de Investigaciones Cardiovasculares Carlos III (CNIC), 28029 Madrid, Spain
[4]Departamento de Bioquímica, Facultad de Medicina, Universidad Autónoma de Madrid (UAM), 28029 Madrid, Spain
[5]Wellcome Sanger Institute, Wellcome Genome Campus, Hinxton CB10 1SA, UK
[6]Wellcome Trust Cancer Research UK Gurdon Institute, University of Cambridge, Tennis Court Road, Cambridge CB2 1QN, UK
[7]Calico Life Sciences LLC, South San Francisco, CA, USA
[8]Department of Chemistry and State Key Laboratory of Marine Pollution, City University of Hong Kong, Kowloon Tong, Hong Kong SAR, China
[9]Department of Psychiatry, UCSF Weill Institute for Neurosciences, University of California San Francisco, San Francisco, CA, USA
[10]School of Biosystem and Biomedical Science, College of Health Science, Korea University, Seoul, Republic of Korea
[11]Shenzhen Research Institute of City University of Hong Kong, Shenzhen, China
[12]Present address: Institute for the Advanced Study of Human Biology (WPI-ASHBi), Kyoto University, Kyoto 606-8501, Japan
[13]Present address: Evergrande Center for Immunologic Diseases, Harvard Medical School and Brigham and Women's Hospital, Boston, MA, USA
[14]These authors contributed equally
[15]Lead contact
*Correspondence: mhemberg@bwh.harvard.edu (M.H.), nadav.ahituv@ucsf.edu (N.A.)
https://doi.org/10.1016/j.xgen.2022.100111

SUMMARY

Alternative DNA conformations, termed non-B DNA structures, can affect transcription, but the underlying mechanisms and their functional impact have not been systematically characterized. Here, we used computational genomic analyses coupled with massively parallel reporter assays (MPRAs) to show that certain non-B DNA structures have a substantial effect on gene expression. Genomic analyses found that non-B DNA structures at promoters harbor an excess of germline variants. Analysis of multiple MPRAs, including a promoter library specifically designed to perturb non-B DNA structures, functionally validated that Z-DNA can significantly affect promoter activity. We also observed that biophysical properties of non-B DNA motifs, such as the length of Z-DNA motifs and the orientation of G-quadruplex structures relative to transcriptional direction, have a significant effect on promoter activity. Combined, their higher mutation rate and functional effect on transcription implicate a subset of non-B DNA motifs as major drivers of human gene-expression-associated phenotypes.

INTRODUCTION

Under physiological conditions, the favored conformation of DNA is a right-handed double helix, also known as B-DNA (Figure 1A). However, alternative DNA conformations, collectively termed non-B DNA structures, have been recognized and shown to affect transcription, replication, recombination, and DNA repair, either transiently or for longer periods.[1] The propensity to form non-canonical structures and their biophysical properties are determined by non-B DNA motifs that can be identified from the primary sequence.[2–5] For example, Z-DNA is a left-handed double-helical structure that is formed by alternating purine-pyrimidine tracts (Figure 1B). G-quadruplexes (G4s) consist of four or more G-runs that are interspersed with loop elements (Figure 1C). Direct and tandem repeats, including mononucleotide repeat tracts, can form slipped structures (Figure 1D); mirror repeats with high A/G content can form triple-stranded DNA structures (Figure 1E); and inverted repeats can form hairpins and cruciforms (Figures 1F and 1G).

Previous studies have shown that non-B DNA structures are mutational hotspots because they are more likely to be exposed as single-stranded DNA, making them vulnerable to damage.[6,7] Their increased mutability results in an excess of population variants overlapping non-B DNA motifs[8,9] and an excess of somatic mutagenesis at those sites in cancer.[10–15] Although variants overlapping non-B DNA motifs are frequently neutral in their effect, it is clear that non-B DNA motifs are a major source of genetic variation in the human genome. They are enriched in regulatory regions[16–19] and likely cause

This is an open access article under the CC BY license (http://creativecommons.org/licenses/by/4.0/).

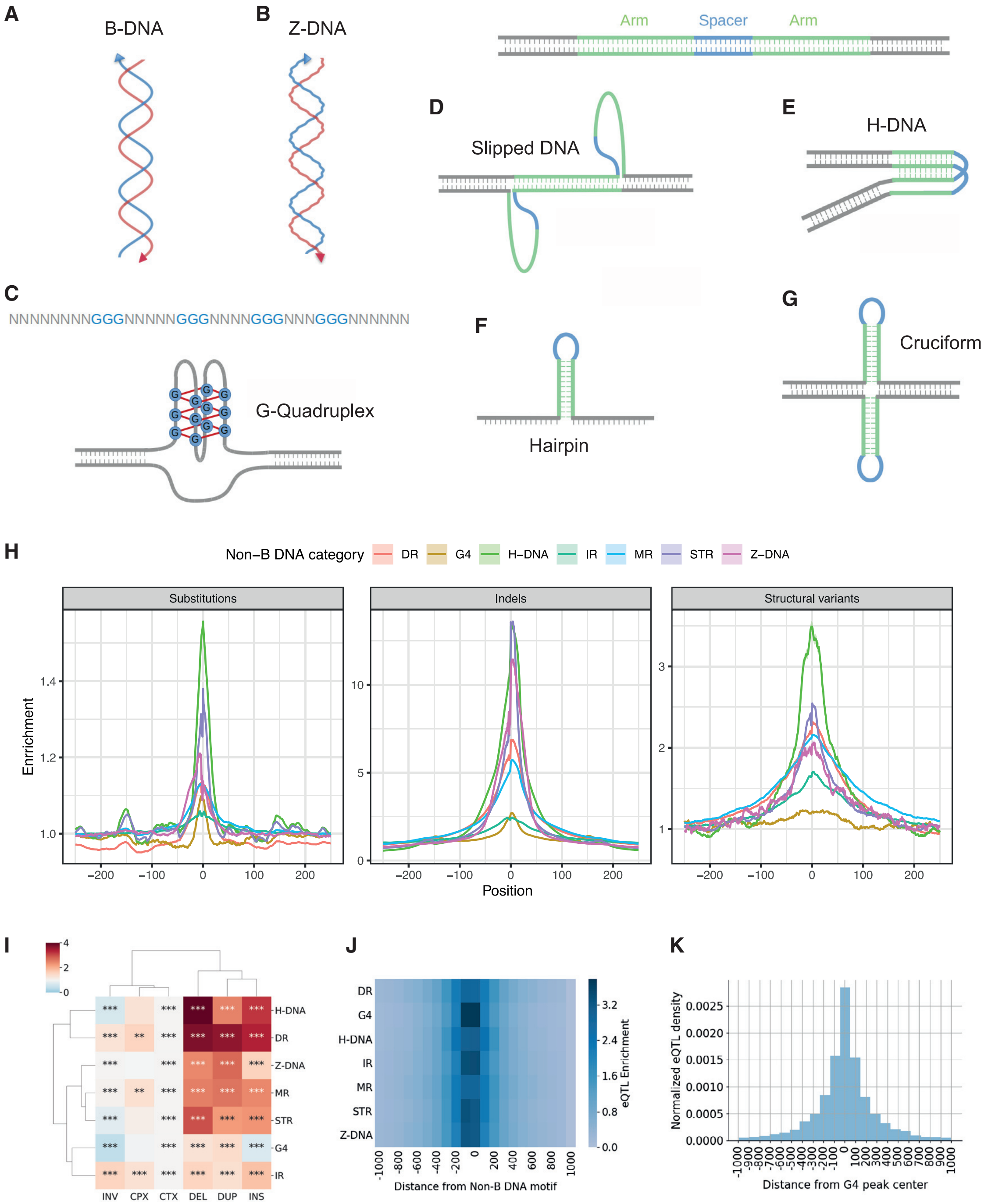

Figure 1. Genomic variants are enriched at non-B DNA motifs

(A–G) Schematic representation of non-B DNA motifs.

(A) Canonical B-DNA structure.

(B) Left-handed double-stranded DNA, known as Z-DNA conformation.

(C) G-quadruplex formation at sites of four G-runs interspersed by looping regions.

(D) Direct and tandem repeats misalign and form slipped DNA structures. The arms are the repeating unit and the spacer the intervening non-repeating part.

(E) A subset of mirror repeats with high AG/TC-content fold into intramolecular DNA structures known as H-DNA. The arms are the repeating unit with mirror symmetry and the spacer the intervening non-repeating part.

(F and G) (F) Inverted repeats fold into hairpin structures, and (G) Inverted repeats can fold into cruciform structures. The arms are the repeating unit with inverted symmetry and the spacer the intervening non-repeating part.

(legend continued on next page)

numerous disorders such as cancer, fragile X syndrome, and Friedreich ataxia.[20–22] As a result, they are likely hotspots for disease and genetic variation.[23] Thus, it is important to take non-B DNA motifs into consideration when modeling mutation rates and pathogenicity.[7,15,24]

In the human genome, non-B DNA motifs are unevenly distributed. They are enriched in certain regulatory regions, including open chromatin, promoters, and 5′ and 3′ UTRs.[16–19] At the base-pair level, specific non-B DNA motifs are over-represented and positioned relative to critical gene features, such as the transcription start and end sites, splice junctions, and translation initiation regions, while their formation is often associated with transcriptionally active loci.[25–31] A number of studies have shown, primarily in cancer when targeting selected loci, that non-B DNA motifs can have an impact on the expression levels of various genes. For example, G4s were shown to modulate the expression of key cancer genes, such as *MYC*, *c-Kit*, *BCL2*, and *KRAS*, with their disruption resulting in pronounced expression changes.[25,32] Furthermore, recurrent mutations across cancer types and patients, including highly recurrent promoter mutations in the *TERT* and *PLEKHS1* genes, overlap non-B DNA motifs[33–35] and likely predispose these regions to increased mutagenesis. However, the functional consequences of non-B DNA motif disruptions, either due to germline or somatic mutations at promoter regions, have not been studied in a systematic manner and remain poorly understood. Additionally, although the impact of promoter non-B DNA structures at individual genes on the regulation of gene expression has been demonstrated at individual loci,[34,36,37] the results are conflicting regarding the role of non-B DNA motifs acting as either activators or repressors.[38]

Here, we set out to systematically identify the role of non-B DNA motifs on promoter transcriptional regulation. We find that non-B DNA motifs harbor an excess of polymorphisms, many of which affect gene expression levels. To gain further insights regarding the impact of non-B DNA motifs on gene expression, we analyzed various lentivirus-based massively parallel reporter assays (lentiMPRAs[39]) to systematically test the effect of non-B DNA motifs on promoter activity. We observed a causal link between specific non-B DNA sequences and gene expression levels. We also show that biophysical properties, which influence the likelihood of secondary-structure formation and stability, are linked to these regulatory effects. Our results demonstrate that non-B DNA motifs are important determinants of promoter activity, and their increased mutability implicates them as major drivers of gene-expression-associated phenotypes.

RESULTS

Non-B DNA motifs harbor an excess of standing genetic variation

As previous studies demonstrated that non-B DNA motifs are enriched for somatic mutations,[11,14,15] we set out to analyze whether this enrichment also exists for germline variation. We took advantage of available whole-genome sequencing (WGS) datasets for thousands of individuals and analyzed them to determine whether non-B DNA sequences are enriched for variants. We measured the genome-wide distribution of 204,063,503 single-nucleotide polymorphisms (SNPs), including both rare and common variants as well as 25,925,202 small insertions and deletions (indels; <50 bp) derived from 15,496 genomes from the gnomAD project[40] relative to seven non-B DNA motifs: inverted repeats (IRs), direct repeats (DRs), mirror repeats (MRs), short tandem repeats (STRs), G4s, Z-DNA, and H-DNA motifs (Figures 1A–1G). To form a null distribution, we generated simulated SNPs, controlling for trinucleotide context and proximity to the original SNP (STAR Methods). We observed an excess of SNPs directly overlapping non-B DNA motifs (Figure S1A; Mann-Whitney U, $p < 0.0001$), but the magnitude of the effect was small, and the highly significant p value was due to the large sample size. Of note, H-DNA motifs and IRs showed the highest (1.56) and lowest (1.05) fold enrichments, respectively (Figures 1H, S1B, and S1C). Similarly, the proportion of indels overlapping non-B DNA motifs was substantially elevated relative to the simulated controls (2.26-fold, Mann-Whitney U, $p < 0.0001$; Figure S1D). The enrichment of genetic variants at individual non-B DNA motifs was higher for small indels than for SNPs, ranging from 2.44-fold for IRs to 13.68-fold for STRs (Figures 1H, S1E, and S1F). We further separated indels into insertions and deletions, finding differences depending on the non-B DNA motif category (Figure S1G). For example, STRs had a higher frequency of deletions, whereas G4s had a higher frequency of insertions.

Extending our analysis to 505,529 structural-variant breakpoints derived from the gnomAD project,[40] we found a strong association with non-B DNA motifs, with 14.61% of structural-variant breakpoints directly overlapping a non-B DNA motif versus 8.83% for simulated controls (Mann-Whitney U, $p < 0.0001$; Figure S1H), representing a 1.66-fold enrichment. For individual non-B DNA motifs, the enrichments ranged from 1.23-fold for G4s to 3.50-fold for H-DNA motifs (Figures 1H and S1I–S1K; Mann-Whitney $p < 0.0001$ for all non-B DNA motifs), consistent with previous reports finding an excess of non-B DNA motifs at structural-variant breakpoints.[41] We separated structural variants into six categories: insertions, deletions,

In schematics (D)–(G), spacer denotes the region of the non-B DNA motif that remains single stranded and exposed, whereas arms hybridize into double-stranded DNA.

(H) Distribution of non-B DNA motifs relative to 204,063,503 SNPs on the left. Distribution of non-B DNA motifs relative to 25,925,202 small indel variants in the center. Distribution of non-B DNA motifs relative to 505,529 structural variants on the right. Enrichment is corrected for trinucleotide context. DR, G4, IR, MR, and STR refer to direct repeats, G-quadruplexes, inverted repeats, mirror repeats, and short tandem repeats, respectively.

(I) Association between structural-variant-breakpoint category and enrichment at non-B DNA motifs. INV, CPX, CTX, DEL, DUP, and INS refer to inversions, complex rearrangements, translocations, deletions, duplications, and insertions, respectively. Adjusted p values displayed as $^{*}p < 0.05$, $^{**}p < 0.01$, and $^{***}p < 0.001$.

(J) Enrichment patterns of eQTLs at non-B DNA motifs relative to proximal regions.

(K) eQTL density at G4 peaks from G4 antibody treatment.

duplications, inversions, translocations, and complex.[40] We found that deletions, insertions, and duplications were the most enriched across non-B DNA motifs (Figure 1I). Taken together, these results suggest that non-B DNA motifs are hotspots of genetic variation in the human population across different categories of population variants.

Non-B DNA motifs are enriched for gene-regulatory-associated variants

To gain further insights regarding the regulatory potential of these variants, we investigated the relative frequency of variants overlapping non-B DNA motifs across six regulatory-element-associated sequences/functions defined by the Ensembl Regulatory Build:[42] promoters, CTCF-binding sites, open chromatin regions, transcription factor binding sites, promoter flanking regions, and enhancers. The analysis was performed across twelve different cell lines (STAR Methods), finding that most non-B DNA motifs were enriched for SNPs, indels, and structural variants across the regulatory elements, but more so for indels than for SNPs and structural variants (Figures S2A–S2C). We also investigated the increase in mutagenicity for non-B DNA motifs across the seven annotated genic sub-compartments: genic, intronic, coding, and 5′ and 3′ UTRs as well as 1 kilobase (kb) upstream of the transcription start site (TSS) and 1 kb downstream of the transcription end site (TES). Most regions had elevated mutation rates, although the magnitude varied by mutation type and genic sub-compartment (Figures S2D–S2F). As expected, coding regions showed the lowest mutagenicity relative to other regions, most likely due to selection constraints and increased DNA repair.[43]

To analyze whether variants in non-B DNA motifs could have a substantial impact on gene expression, we analyzed expression quantitative trait loci (eQTL). We examined the frequency of eQTLs, characterized by the GTEx consortium,[44] at each of the seven non-B DNA motifs genome wide. We found an enrichment of eQTLs across all non-B DNA categories relative to their flanking regions, with the most pronounced effect for G4s (Figure 1J). Although the excess of eQTLs in the vicinity of non-B DNA motifs can be explained by the higher background frequency of substitution and indel SNPs (Figure 1H), our results indicate that a subset of mutations overlapping non-B DNA motifs impact gene expression.

As G4s had the most pronounced effect on gene expression, we next analyzed G4 sequencing (G4-seq) and G4 chromatin immunoprecipitation (ChIP)-seq datasets for their overlap with population variants and eQTLs. We investigated the association between population variants and G4s using previously published G4-seq datasets from the HEK-293T cell line with Pyridostatin (PDS) and K^+ treatments that provide *in vitro* evidence of G4 formation potential[45] and G4 ChIP-seq-derived peaks from the HaCat cell line that provide *in vivo* evidence of sites that form G4 structures.[16] In accordance with the G4 motif analysis, we found that SNPs, indels, and structural variants were enriched at G4-seq and G4 ChIP-seq peaks (Figures S3A–S3F; Mann-Whitney U, $p < 0.001$). We considered the G4 ChIP-seq sites that overlapped both G4-seq K^+ and G4-seq PDS peaks as the highest confidence, experimentally derived G4s (Figure S3G) and found consistent enrichments of 1.14-fold, 1.41-fold, and 1.36-fold for substitutions, small indels, and structural variants (Figures S3H and S3I). Next, we found that eQTLs are found more frequently than expected by chance in the experimentally derived G4 sites. In total, 20,310 eQTLs overlapped with the 8,955 ChIP-seq peaks, with 34% of the peaks having one or more eQTL (Figures 1K and S3J). Interestingly, the enrichment for the experimentally derived G4s was more pronounced than our results derived from the G4 motif analysis. This is likely the result of G4 formation occurring more frequently in open chromatin and transcribed regions.[16]

We also investigated if G4 ChIP-seq peaks overlapping genes display a preference for the template (non-coding) or non-template (coding) strands, using the G4 motif orientation within the peaks as proxy. After correcting for the background bias in the orientation of G4 motifs (Figure S4A), we found that G4 motifs on the non-template strand overlap G4 ChIP-seq peaks 1.71-fold more frequently than motifs on the template strand (binomial test, $p < 1 \times 10^{-12}$) (Figures S4B and S4C), suggesting significant bias in the formation of G4s, dependent on their orientation.

Non-B DNA motifs are enriched in promoter regions

We next investigated the distribution of non-B DNA motifs across the six regulatory elements defined by the Ensembl Regulatory Build (promoters, CTCF-binding sites, open chromatin regions, transcription factor binding sites, promoter flanking regions, and enhancers). For most non-B DNA motifs, we found an enrichment at promoters and CTCF-binding sites relative to other regulatory elements (Figures 2A and S5A), in accordance with previous findings.[46] Next, we separated the gene body into six compartments: a 1 kb window upstream from the TSS, the 5′ and 3′ UTRs, coding exons and introns, and a 1 kb window downstream of the TES. Consistently, promoter regions displayed a higher density of non-B DNA motifs than the gene body for most non-B DNA motifs, with the enrichment ranging from 0.97-fold for IRs to 3.02-fold for G4s (Figures 2B and S5B). We also found a significant enrichment of G4-seq-derived peaks for both PDS and K^+ treatments and for G4 ChIP-seq-derived peaks at promoters relative to other regulatory elements (Figure 2C). Across the gene body, we found the highest enrichments at promoters, coding regions, and 5′ UTRs (Figure 2D).

At promoters, for most non-B DNA motifs, the enrichment was higher upstream of the TSS than in the broader promoter region (Figure 2E). A close investigation of the distribution of non-B DNA motifs relative to the TSS showed an enrichment of peaks $\sim$50 bp upstream of the TSS ranging between 1.28- and 1.89-fold for DRs and G4 motifs, respectively (Figure 2F). Importantly, we observed a 5-fold enrichment approximately 100 bp upstream of the TSS for G4 ChIP-seq peaks, consistent with the literature.[16] Interestingly, the ChIP-seq-derived enrichment was substantially larger than that of the G4 motif and the G4-seq datasets (Figure 2G), reflecting a preference in structure formation at promoters *in vivo*. We also performed a Gene Ontology (GO) term analysis in promoter upstream regions. For G4s, Z-DNA motifs, and MRs, we found multiple terms associated with developmental processes, such as pattern specification process (GO: 0007389), embryonic organ development (GO: 0048568), and positive regulation of neuron differentiation (GO: 0045666) (Figure S6A). As these analyses suggest that some non-B DNA motifs could control tissue-specific gene expression, we used TissueEnrich to calculate the

CellPress
OPEN ACCESS

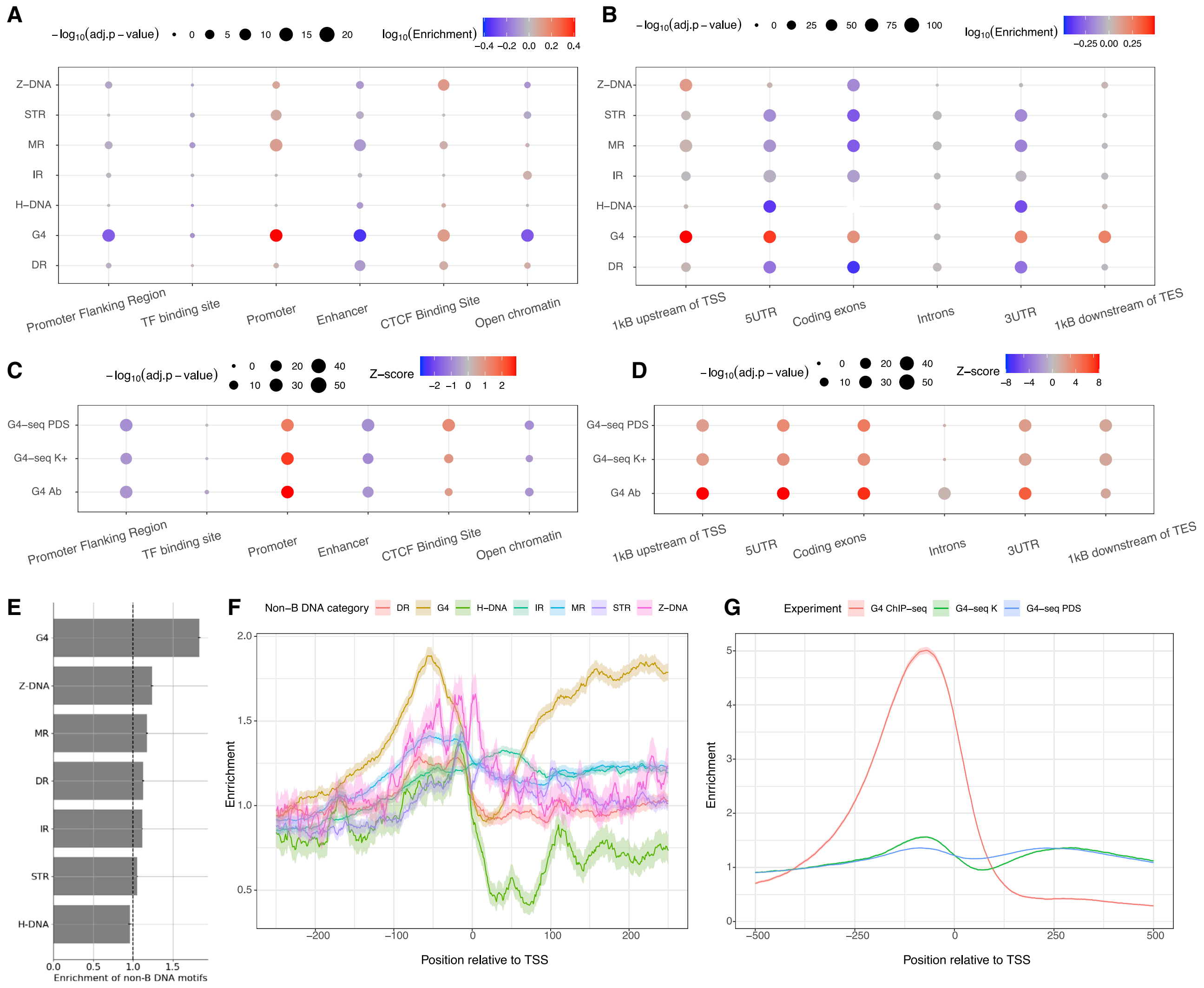

Figure 2. Non-B DNA motifs at functional elements

(A) Median relative enrichment across 12 cell lines for non-B DNA motif enrichment at Ensembl Regulatory Features.

(B) Non-B DNA motif enrichment at functional genomic compartments for each non-B DNA motif. Statistical significance was estimated using Binomial tests with Bonferroni correction.

(C) *Z* score of G4-seq and G4 ChIP-seq peak density across Ensembl Regulatory Features.

(D) *Z* score of G4-seq and G4 ChIP-seq peak density across the gene body.

For (C) and (D), two treatments that stabilize G4s, PDS and K^+, were used in G4-seq.

(E) Enrichment of non-B DNA motifs in the [–250, 0] region relative to the wider promoter region (–1 kB, 0). Error bars represent standard deviation from bootstrapping.

(F) Base-pair resolution of distribution of nucleotide motifs relative to the TSS. IRs, MRs, DRs, STRs, and G4s are abbreviations for inverted repeats, mirror repeats, direct repeats, short tandem repeats, and G-quadruplexes, respectively.

(G) G4 enrichment patterns relative to the TSS for G4 motif, G4-seq peaks in K^+ and PDS treatments, and from G4 ChIP-seq peaks.

enrichment of tissue-specific genes and found sets of tissue-specific genes where a set of neuronal-specific genes were enriched for genes containing G4, MR, DR, and STR at their upstream promoter regions (Figure S6B). Altogether, these results demonstrate that promoters are enriched for non-B DNA motifs relative to other regulatory elements and to other genic compartments and that some non-B DNA motifs are more likely to occur at developmental and neuronal genes. Therefore, the excess of genetic variants at non-B DNA motifs identified earlier could have broad implications on gene regulation expression levels across tissues and developmental stages.

MPRAs identify G4 and Z-DNA to have a substantial effect on gene expression

The enrichment of non-B DNA motifs at promoters and the excess of eQTLs localized within certain non-B DNA motifs

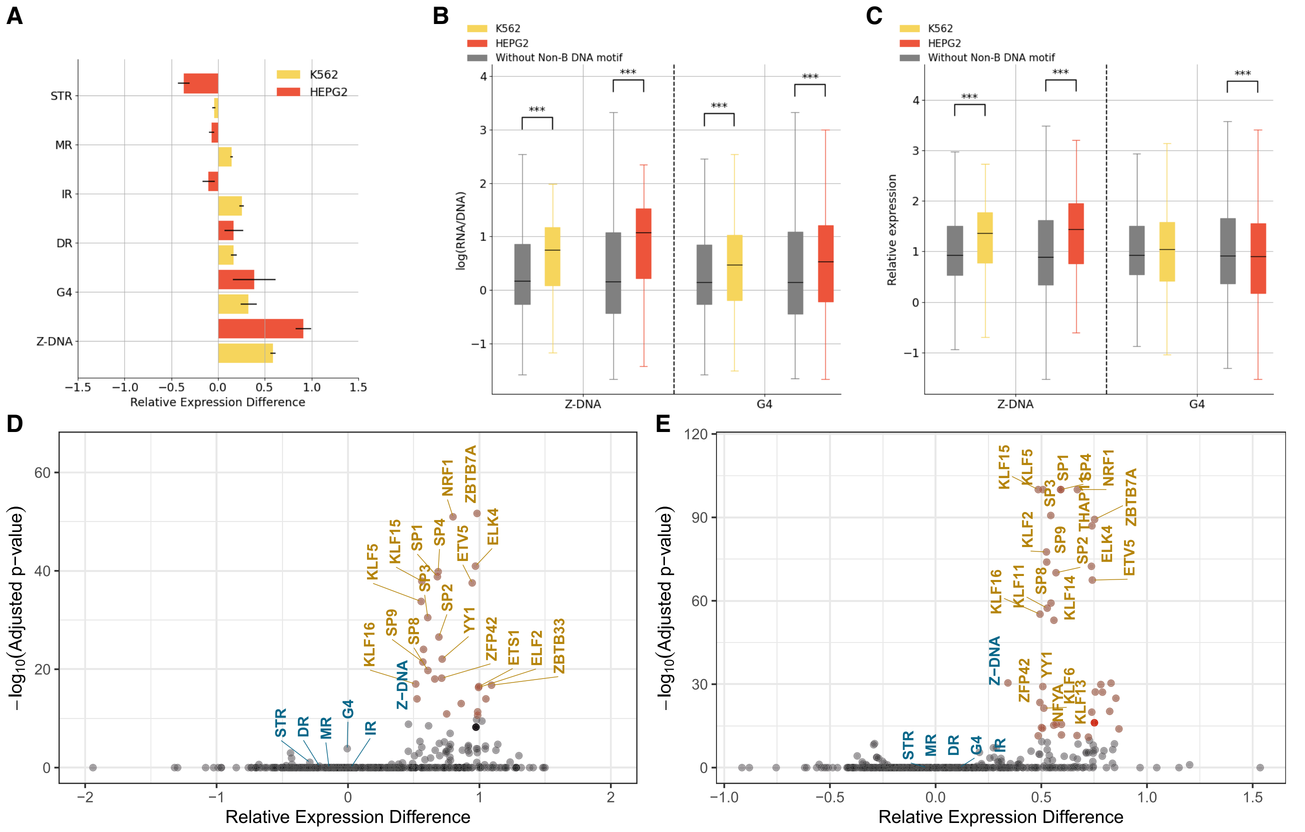

Figure 3. Contribution of sequences with non-B DNA motifs toward gene expression

(A) Association between presence of different non-B DNA motifs and expression. Median differences in expression of sequences with and without each non-B DNA motif are shown. Error bars show standard deviation from bootstrapping.

(B) Comparative analysis of sequences with and without G4s and Z-DNA motifs.

(C) Comparative analysis of sequences with and without G4s and Z-DNA motifs controlling for GC content.

(B and C) Statistical significance was calculated with t tests and Bonferroni correction.

(D and E) Relative expression differences between the median expression for sequences with and without non-B DNA motifs and transcription factor binding sites in (D) HepG2 and (E) K562 lentiMPRA.

Statistical significance is estimated with t tests and Bonferroni correction. In (B) and (C), adjusted p values displayed as *$p < 0.05$, **$p < 0.01$, and ***$p < 0.001$.

prompted us to investigate their functional impact on gene transcription utilizing MPRAs. We first analyzed two lentiMPRA datasets generated by our group as part of the ENCODE consortium,[47] where a total of 14,625 and 7,346 candidate promoter sequences were examined in both orientations in K562 and HepG2 cell lines. We identified non-B DNA motifs across the lentiMPRA tested sequences (STAR Methods) and examined their association with gene expression. We found that sequences with G4 and Z-DNA motifs showed significantly increased expression levels in both cell lines (Figures 3A and 3B; t tests, Bonferroni correction, $p < 0.001$), while for IRs, DRs, STRs, and MRs we did not observe consistent results (Figure S7A). As there is a known positive correlation between expression and guanine-cytosine (GC) content,[48] which was also observed in our lentiMPRA datasets (Pearson r = 0.398 and 0.261 in K562 and HepG2, respectively), we constructed a linear model to account for the contribution of GC content toward expression (Figure S7B). Sequences with Z-DNA motifs had substantially elevated expression levels relative to sequences without them, even after controlling for GC content in both cell lines (t tests, Bonferroni correction $p < 0.001$; Figures 3C and S7C). However, after GC-content correction, G4s were not associated with increased expression, and in HepG2, they were instead significantly associated with reduced expression levels (Figures 3C and S7C). Similar results were obtained after removing outliers from the linear model (absolute Z score >2.5). Also, G4s on the template strand were associated with reduced expression relative to non-template strands in both cell lines, but the difference reached statistical significance only in the HepG2 cell line (Figure S7D). For the other non-B DNA motifs, we could not find consistent effects in both cell lines, suggesting that nucleotide composition contributed to the observed effects before GC-content correction.

Finally, we identified transcription factor binding sites (TFBSs) across the MPRA sequences using the JASPAR vertebrate non-redundant list of transcription factor motifs.[49] We compared the contribution of non-B DNA motifs relative to TFBSs toward expression levels, both before and after GC-content correction.

We found that G4 and Z-DNA motifs had similar contributions to known TFBSs, such as EGR1, YY1, and SP9, resulting in increased expression levels relative to sequences without them (Figure S8). However, only Z-DNA motifs had comparable effects when we accounted for GC content (Figures 3D and 3E), and the results were consistent between HepG2 and K562 lentiMPRAs.

To further validate our findings, we analyzed lentiMPRA results from a library that characterized the effect of 3,623 *de novo* promoter mutations that were identified in the Simons Simplex Collection.[50] This library tested both alleles, centered around the variant, totaling 7,246 sequences along with 150 positive and 150 negative controls for their effect on promoter activity in neural progenitor cells (NPCs) (Figures S9A–S9C). This library had 1,234 sequences harboring one or more non-B DNA motifs (Figure S9D). We observed that sequences harboring G4, DR, and Z-DNA motifs displayed a significantly higher expression than sequences without them (t tests, Bonferroni corrected p values, G4s, DRs, and Z-DNA $p < 0.001$), whereas sequences with IRs, MRs, and STRs did not show a significant association ($p > 0.05$) (Figure 4A).

Similar to the analysis of the ENCODE MPRA libraries, we observed a significant contribution of the GC content toward the effects on expression of certain non-B DNA motifs. After constructing a linear model to adjust for GC content, we observed that G4 motifs are associated with decreased expression, while only Z-DNA sequences remained associated with higher expression (Figure 4B), consistent with previous results. In this case, removing outliers maintained a positive association with G4s and gene expression. We also observed a substantial difference in the expression dependent on the orientation of G4 motifs, with G4s on the template strand having lower expression than those on the non-template strand before and after GC-content adjustment (Figures 4C and S9E; Mann-Whitney U, $p < 0.001$). The primary sequence comprising consecutive G-runs that are interspersed by loop elements can form G4 structures (Figure 1). The association between G-runs and gene expression was further investigated, finding that consecutive G-runs result in decreased expression when accounting for their GC-content contribution (Figure S9F). Furthermore, we found that the length of the Z-DNA motif was positively associated with the expression levels (Kruskal-Wallis H test, $p < 0.001$; Figure 4D).

Similar to the previous MPRAs, we identified TFBSs across the MPRA sequences and compared the contribution of non-B DNA motifs relative to TFBSs toward expression levels before and after GC-content correction. We found that G4 and Z-DNA motifs had comparable contributions to TFBSs toward increasing expression levels with increases of 1.27- and 1.51-fold over sequences without them (Figure S10A). However, when we accounted for GC content, the effect of non-B DNA motifs was not comparable to the best TFBS motifs (Figure S10B). Therefore, we find substantial differences in the results in NPCs relative to HepG2 and K562 cell lines, with a lower contribution of Z-DNA motifs in NPCs, which might be due to the selection of loci that were not necessarily proximal to the TSS or due to the lower number of Z-DNA-containing sequences, with only 311 sequences having them.

To validate if the G4s we observed in this NPC lentiMPRA form these structures, we selected ten candidate promoter-proximal sequences with the lowest and highest expression among sequences with G4s (Table S1) and performed multiple spectroscopic assays to characterize their structures (Figures 4E and 4F), as G4 structures possess distinct spectroscopic features.[51,52] We first used circular dichroism spectroscopy measurements of the G4-containing DNA oligonucleotides, in the presence of lithium ions (non-G4 stabilizing) or potassium ions (G4 stabilizing), to examine the formation potential of DNA G4s, which indicated that our candidate sequences can fold into G4 structures (Figures 4E, 4F, S11A, and S11B). In addition, we conducted UV melting and found a hypochromic shift at 295 nm for the potassium-ion condition, which supported the formation of the G4 structure, with a melting temperature above physiological temperature (Figures 4E, 4F, S11A, and S11B).

To confirm the results from the circular dichroism and UV-melting experiments, we used fluorescent-based arrays, including N-methyl mesoporphyrin IX (NMM)-ligand-enhanced fluorescence and intrinsic fluorescence experiments (Figures 4G, 4H,S12, and S12B). In the absence of NMM ligand, no fluorescence was observed at ~610 nm. Upon NMM addition, weak fluorescence was observed under Li^+, which was substantially enhanced when substituted with K^+, supporting the formation of G4 that allows recognition of NMM and enhances its fluorescence (Figure 4G). Similarly, the intrinsic fluorescence of G4s was increased when replacing Li^+ with K^+, highlighting the formation of DNA G4s (Figure 4H). Corroborating our results, we observed increased fluorescence intensity under conditions that promote G4 formation for all candidates. We also carried out two positive G4 controls and a negative B-DNA control to verify our findings above (Figure S13). Combined, these results validate that these sequences form G4 structures *in vitro*.

Non-B DNA motifs have a significant effect on promoter activity

To directly test the effect of non-B DNA structures on promoter activity, we generated an MPRA library that introduces various non-B DNA perturbations to ten disease-associated genes. This set of genes included cancer oncogenes (*CMYC*, *CKIT*, *BCL2*, *KRAS*) and genes associated with different cancer types (*ADAM12*, *ALOX5*, *SRSF6*, *VEGF12*) as well as *FMR1*, associated with fragile X syndrome (OMIM: 300624), and *SNX12*, which is associated with neurodegenerative diseases (Table S2). As our MPRA-tested sequences are 200 bp in length, we first validated whether our selected 200 bp sequences could drive promoter activity using luciferase assays in K562, MCF7, IMR90, and HEK293T cells, finding the majority to be active in most cell lines (Figures S14A and S14B).

Following validation of these 200 bp sequences, we next generated an MPRA library that included the following manipulations: (1) disruption of existing non-B DNA motifs and (2) introduction of different non-B DNA motifs with varied biophysical properties, including spacer- and arm-length changes in IRs, DRs, and MRs, orientation and loop length in G4s, and length in Z-DNA motifs. lentiMPRAs and subsequent computational analyses were carried out as previously described.[53] Briefly, oligonucleotides were synthesized and cloned into a lentiviral MPRA promoter vector (Figure 5A; Table S2), and lentivirus libraries were generated. Libraries were used to infect both K562 and

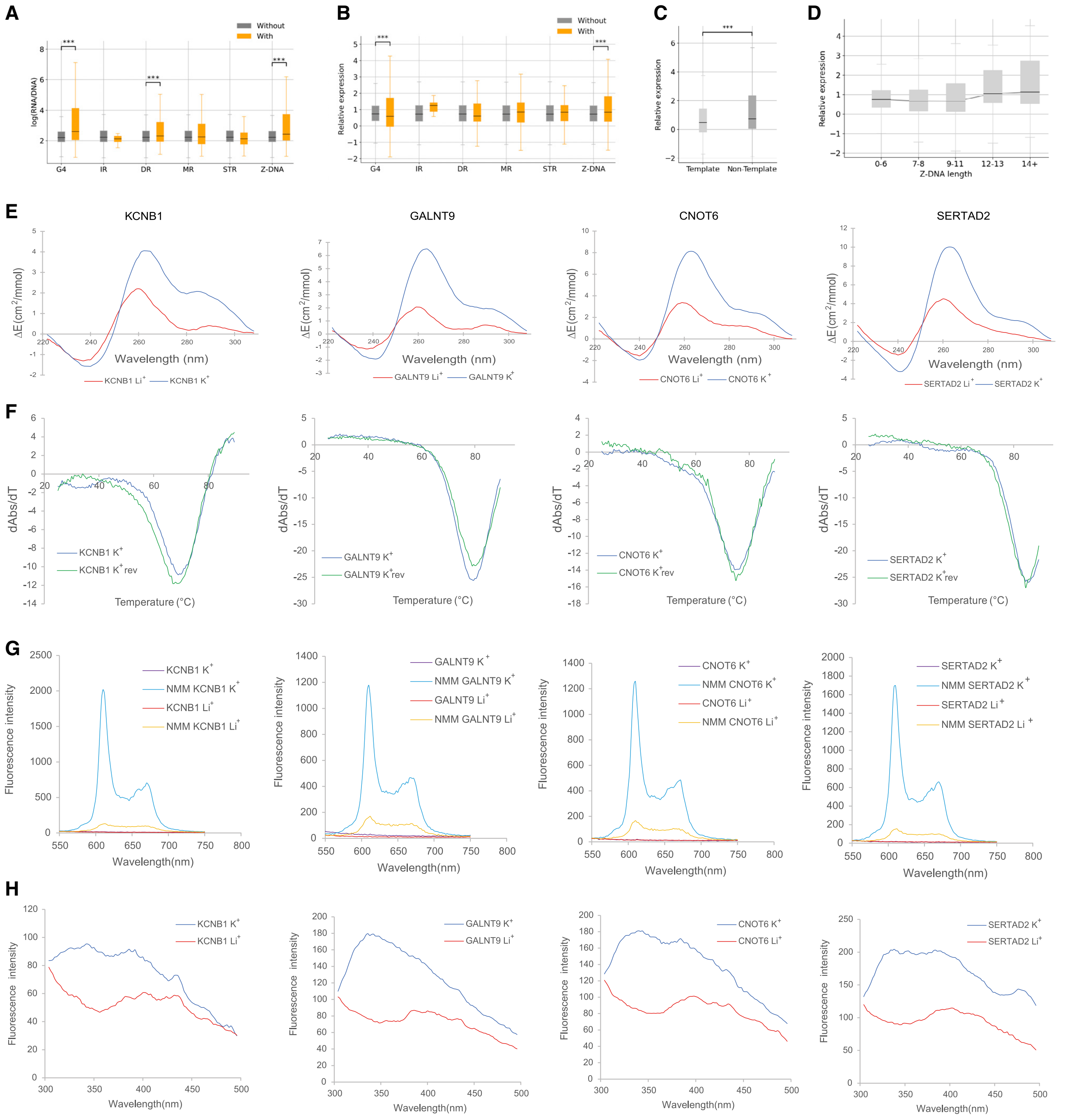

Figure 4. Expression-associated variants relative to non-B DNA motifs

(A and B) Expression of sequences with and without each of the non-B DNA motifs: (A) without adjusting for GC content and (B) adjusting for GC content. t tests with Bonferroni correction were performed.

(C) Expression is associated with the orientation of G4s at promoters.

(D) The length of Z-DNA motifs was associated with increased gene expression (Kruskal-Wallis H test, $p < 0.001$).

(E) Circular dichroism (CD) spectra of the four candidate targets for G4 formation potential in presence of two cations.

(F) UV-melting profiles of the four G4 candidates in presence of K^+. The reverse melting profile (K^+_{rev}) is also shown and matched well with the forward melting profile (K^+). Hypochromic shift at 295 nm is a hallmark for G4 formation, which can be transformed into a negative peak in derivative plot (dAbs/dT) for G4 stability analysis. The melting temperature (Tm) of a G4 can be identified at the maximum negative value.

(G) Fluorescence emission associated with NMM ligand binding to G4 candidates in the presence of Li^+ or K^+ ions.

(H) Intrinsic fluorescence of four candidate DNA oligonucleotides under Li^+ or K^+ conditions.

In (A)–(C), adjusted p values displayed as $^*p < 0.05$, $^{**}p < 0.01$, and $^{***}p < 0.001$.

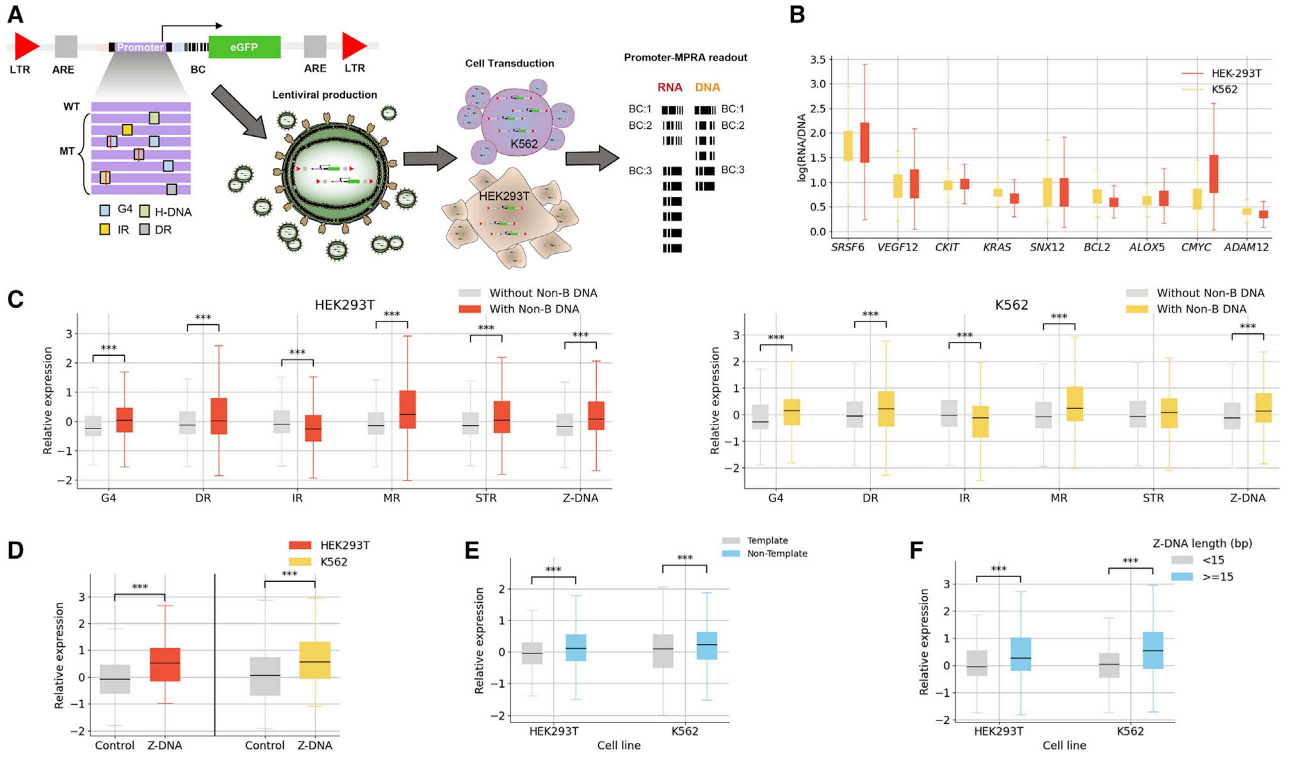

Figure 5. Characterization of non-B DNA motifs across nine promoter templates

(A) Schematic summary of the experimental design for the promoter lentiMPRA. An example of one of the promoters is depicted at the top left with several non-B DNA motifs, and several mutations are shown at the bottom (site mutations) and on the right (duplication/substitution) for G4s. The collection of all promoters is ordered as an oligonucleotide library of 230-mer. The oligonucleotide library is PCR amplified and barcoded at the 5′ UTR using a degenerate reverse primer. Cloning of PCR products into a lentiviral promoter assay vector was performed next. Cloning of PCR products into a promoter-less lentiviral vector was then performed. This plasmid library was sequenced to assign every barcode to one of the promoters in the library (left) and used to produce the lentiviral library (bottom), which was then used to infect the cell lines of interest (K562 and HEK293T). RNA and DNA were collected after 3 days post-infection, and the barcodes were sequenced. Promoter activity was calculated as the log(RNA/DNA). LTR, long terminal repeats; ARE, antirepressor element.

(B) Expression levels of nine genes and their sequence variants for K562 and HEK-293T cell lines.

(C) Boxplot displaying the *Z* score, for sequences with and without each non-B DNA motif, calculated separately for each gene, in K562 and HEK293T cell lines.

(D) Sequences with Z-DNA motifs display higher expression than sequences with Z-DNA disruptions for *SNX12* and *SRSF6* genes.

(E) Sequences with G4 motifs on the non-template strand have a higher expression than sequences with G4 motifs at the template strand.

(F) Sequences with longer Z-DNA motifs display higher expression.

(A–F) Mann-Whitney U tests with Bonferroni correction were performed, showing significant difference in sequences with and without the displayed non-B DNA motifs, $p < 0.05$ in all cases.

HEK293T cells for 3 days, to allow non-integrating lentivirus to degenerate, and DNA and RNA barcodes were sequenced. Since previous work in our lab showed that lower basal activity can have a significant effect on MPRA results,[54] these two cell lines were chosen as almost all the selected promoters showed $\geq$2-fold activity compared with empty vectors (except for *CKIT* in HEK293T). All experiments were done in triplicate, and computational analyses were carried out using MPRAflow[53] and MPRAnalyze.[55] We observed a strong correlation between all three replicates (Pearson r $\geq$ 0.9 in all cases; Figure S15A) and between the two cell lines (Pearson r = 0.87; Figure S15B).

The promoters in our MPRAs showed variable expression, with the highest levels observed for *SRSF6* and the lowest for *ADAM12* (Figure 5B). We investigated the contribution of each non-B DNA motif toward expression in both cell lines across the promoters, adjusting across genes using *Z* score normalization. Specifically, for each gene we calculated the *Z* score of each sequence, which was calculated by subtracting the expression levels of that sequence from the mean across all sequences of that gene and dividing by the standard deviation. In concordance with our previous MPRA analyses, we observed that sequences with Z-DNA and G4 motifs had significantly higher expression (Figures 5C and 5D). Interestingly, while we did not observe consistent results in our previous MPRA analyses for MRs, DRs, and IRs, here, we observed significantly higher expression levels when MRs and DRs were present, whereas for IRs we found significantly lower expression (Figure 5C). For STRs, we did not find consistent patterns in the two cell lines. The above results across non-B DNA motifs did not change when we accounted for GC content; however, this was most likely due to our experimental design having only a small number of loci targeted, which, as a result, had a narrow and uninformative GC-content range.

For G4s, we introduced a single, two, or three mutations in one, two, three, or every G-run at the original G4 genomic sites. We compared the mutated sequences with the original sequence and found that sequences with disruptions in the G-runs did not display significant expression differences from the original sequences (Figure S15C). We designed MPRA sequences with scrambled Z-DNA motifs or with disruptions of purines to pyrimidines in the alternating purine-pyrimidine tract, which served as Z-DNA controls. We found that there was a statistically significant reduction in expression following the disruption of Z-DNA motifs (Figure 5D), supporting the notion that they are activating sequences. We also observed that non-template G4s had higher expression than those at the template strand in both cell lines and both before and after GC-content correction (Mann-Whitney U, Bonferroni corrected; Figure 5E), consistent with our earlier results. For Z-DNA, longer motifs resulted in higher expression (Figure 5F). These results suggest that the non-B DNA motifs and their biophysical properties contribute to expression across promoter templates.

DISCUSSION

By analyzing thousands of WGS datasets, we found that non-B DNA motifs are hotspots for genetic variation, fitting with their known increased mutability properties. Their increased mutability is consistently observed across mutation types, including substitutions but also larger and more disruptive indels and structural variants. The increased likelihood of mutagenesis at non-B DNA motifs is also consistent with previous analyses of somatic mutations in cancer genomes.[15] Different mechanisms underlying the higher mutation rate at individual non-B DNA motifs have been previously identified, such as DNA polymerase slippage errors at microsatellites causing deletions,[20] which was also observed in this study. We also observed an excess of eQTLs in the vicinity of non-B DNA motifs. In particular, at experimentally identified G4s, the eQTL enrichment was even larger than that observed across G4 motifs (Figures 1J and 1K), which is likely due to the formation of G4 motifs being more frequent in open chromatin regions and nucleosome-depleted regions.[16] We further show that non-B DNA motifs are enriched in promoters where they can directly influence downstream gene expression levels. Specifically, we observed that Z-DNA motifs increase expression, whereas the effect of G4s is dependent on the gene studied. Combined, these results suggest that gene-regulatory variants are more likely to occur at non-B DNA structures and that they have a substantial impact on gene expression.

The promoter effects of G4s have previously been shown to be inhibitory or activating depending on the target gene.[56–58] Similarly, previous work has suggested that Z-DNA sequences can act as both activating and repressing elements in promoters.[29,59,60] Here, we found that in the absence of chemical perturbations, Z-DNA sequences are more likely to be activating, while G4s are more likely to be inhibitory and promoter dependent. One of the mechanisms by which Z-DNA motifs might increase gene expression might be the reduction of nucleosome occupancy that they elicit.[60] The reduction of expression at promoters with G4 motifs could be due to interference with transcription factor or RNA polymerase II binding. In addition, template G4s have a more inhibitory effect than non-template ones. The stronger inhibitory effect at the template strand is also aligned with potentially interfering with RNA polymerase II binding. These results are suggestive of inhibitory effects of G4s in promoters, which can be mischaracterized if the effect of GC content is not taken into consideration, as well as orientation-dependent regulatory effects.

Non-B DNA structure formation depends on a plethora of factors, including DNA superhelicity as well as the activity of multiple enzymes such as topoisomerases and helicases.[61,62] Small molecules that stabilize G4s can substantially alter the thermodynamic equilibrium of structure formation, resulting in dramatic changes in gene expression.[63,64] Thus, targeting these sequences in key regulatory sites could be a potential novel therapeutic path.[65] Although the selectivity of such compounds is usually limited, molecules that discriminate among G4s have also been characterized.[66] These can modulate the activity of clinically important genes, as recently shown for the telomerase gene (*TERT*), where promoter mutations have been associated with a variety of cancers.[67] By targeting a G4 in the *TERT* promoter with a small molecule, the expression of telomerase was down-regulated in cancer cells.[34] However, small molecules targeting G4s could cause concomitant DNA damage and telomere dysfunction, influence telomere length, and interfere with other biological processes.[63] Targeting these non-B DNA structures via *cis*-regulation therapy could be an alternate approach to alter target gene expression.[68]

It is increasingly recognized that non-B DNA motifs are involved in a plethora of cellular processes, such as transcription and translation initiation, splicing, and transcription termination.[26–29,69–81] Therefore, future work is required to explore the regulatory effects of mutations at non-B DNA motifs genome wide and to estimate their overall pathogenicity by integrating the topology of non-B DNA motifs and the downstream biological effects of their disruption. In addition, measuring the likelihood of mutagenesis for individual non-B DNA motifs per cell division in somatic and cancer cells could have important implications relevant to modeling cancer evolution and aging. Further systematic and high-throughput functional assays could extend our understanding of the functional diversity and clinical evaluation of particular non-B DNA motifs and the variants within them.

Limitations of the study

Our study has multiple limitations. First, the examination of the regulatory roles of non-B DNA motifs through MPRA experiments did not investigate how molecules that stabilize their formation affect the conclusions reached. Secondly, the MPRA results are based on specific cell lines, and it would be of interest to examine which of these findings can be generalized across cell types and which effects are cell-type specific. We also cannot exclude the influence of the experimental design in our findings. Furthermore, additional experiments and mechanistic work are required to further our understanding, including biophysical and molecular experiments. Lastly, future work would be needed to resolve the relevance of mutations at non-B DNA motifs in the development and progression of

human diseases. The aforementioned limitations could be of high interest for future work.

STAR★METHODS

Detailed methods are provided in the online version of this paper and include the following:

- KEY RESOURCES TABLE
- RESOURCE AVAILABILITY
  - Lead contact
  - Materials availability
  - Data and code availability
- EXPERIMENTAL MODEL AND SUBJECT DETAILS
- METHOD DETAILS
  - Genomic elements
  - Ensembl regulatory build
  - Non-B DNA motif identification
  - G4-seq and G4 ChIP-seq maps
  - Transcription factor binding site maps
  - Population variant analysis
  - eQTL analysis
  - Gene set enrichment analysis
  - Luciferase assay
  - lentiMPRA of promoters
  - MPRA analysis pipeline
  - NMM ligand enhanced fluorescence
  - Circular dichroism (CD) spectroscopy
  - Thermal melting monitored by UV spectroscopy
  - Intrinsic fluorescence spectroscopy
- QUANTIFICATION AND STATISTICAL ANALYSIS
  - Population variant analysis
  - Transcription factor binding
  - MPRA analysis

SUPPLEMENTAL INFORMATION

Supplemental information can be found online at https://doi.org/10.1016/j.xgen.2022.100111.

ACKNOWLEDGMENTS

This work is supported by the National Human Genome Research Institute (1UM1HG009408, R01HG010333, 1R21HG010065, UM1HG011966, and 1R21HG010683 to N.A.), the National Institute of Mental Health (1R01MH109907 and 1U01MH116438 to N.A.), and the National Heart, Lung, and Blood Institute (R35HL145235 to N.A.). G.E.P. and M.H. were supported by a core grant from the Wellcome Trust. Funding for open access charge was provided by NHGRI. The C.K.K. lab is supported by the Shenzhen Basic Research Project (JCYJ20180507181642811); the Research Grants Council of the Hong Kong SAR, China Projects (CityU 11100421, CityU 11101519, CityU 11100218, and N_CityU110/17); the Croucher Foundation Project (9509003); the State Key Laboratory of Marine Pollution Director Discretionary Fund; City University of Hong Kong projects (7005503, 9667222, and 9680261) to C.K.K.; and National Research Foundation of Korea grant 2020R1C1C1003426 (to J.-Y.A.). O.E. is supported by a California Institute of Regenerative Medicine fellowship (SFSU EDUC2-08391). The sequencing was carried by the DNA Technologies and Expression Analysis Core at the UC Davis Genome Center, supported by NIH Shared Instrumentation grant 1S10OD010786-01.

AUTHOR CONTRIBUTIONS

I.G.-S., M.H., and N.A. conceived the study. I.G.-S., G.E.P., V.A., A.M., and J.Z. wrote the code and performed the analyses. I.G.-S., G.E.P., J.Z., and J.V. generated the visualizations. J.V., O.E., and F.I. performed the MPRA experiments. H.Y.W. and M.I.U. performed the circular dichroism (CD) titration, UV melting, and fluorescence assays, and H.Y.W., M.I.U., and C.K.K. analyzed and interpreted the spectroscopic data. J.-Y.A. and S.J.S worked on the NPC MPRA design. M.H. and N.A. supervised the research. I.G.-S., M.H., and N.A. wrote the manuscript with input from all authors.

DECLARATION OF INTERESTS

The authors declare no competing interests.

Received: March 23, 2021
Revised: October 21, 2021
Accepted: February 18, 2022
Published: March 15, 2022

REFERENCES

1. Ghosh, A., and Bansal, M. (2003). A glossary of DNA structures from A to Z. Acta Crystallogr. D Biol. Crystallogr. *59*, 620–626.
2. Nag, D.K., and Petes, T.D. (1991). Seven-base-pair inverted repeats in DNA form stable hairpins in vivo in Saccharomyces cerevisiae. Genetics *129*, 669–673.
3. Leach, D.R. (1994). Long DNA palindromes, cruciform structures, genetic instability and secondary structure repair. Bioessays *16*, 893–900.
4. Lobachev, K.S., Shor, B.M., Tran, H.T., Taylor, W., Keen, J.D., Resnick, M.A., and Gordenin, D.A. (1998). Factors affecting inverted repeat stimulation of recombination and deletion in Saccharomyces cerevisiae. Genetics *148*, 1507–1524.
5. Tippana, R., Xiao, W., and Myong, S. (2014). G-quadruplex conformation and dynamics are determined by loop length and sequence. Nucleic Acids Res. *42*, 8106–8114.
6. Pannunzio, N.R., and Lieber, M.R. (2018). Concept of DNA lesion longevity and chromosomal translocations. Trends Biochem. Sci. *43*, 490–498.
7. Gonzalez-Perez, A., Sabarinathan, R., and Lopez-Bigas, N. (2019). Local determinants of the mutational landscape of the human genome. Cell *177*, 101–114.
8. Du, X., Gertz, E.M., Wojtowicz, D., Zhabinskaya, D., Levens, D., Benham, C.J., Schäffer, A.A., and Przytycka, T.M. (2014). Potential non-B DNA regions in the human genome are associated with higher rates of nucleotide mutation and expression variation. Nucleic Acids Res. *42*, 12367–12379.
9. Guiblet, W.M., Cremona, M.A., Harris, R.S., Chen, D., Eckert, K.A., Chiaromonte, F., Huang, Y.-F., and Makova, K.D. (2021). Non-B DNA: a major contributor to small- and large-scale variation in nucleotide substitution frequencies across the genome. Nucleic Acids Res. https://doi.org/10.1093/nar/gkaa1269.9.
10. Wang, G., and Vasquez, K.M. (2006). Non-B DNA structure-induced genetic instability. Mutat. Res. *598*, 103–119.
11. Wang, G., Christensen, L.A., and Vasquez, K.M. (2006). Z-DNA-forming sequences generate large-scale deletions in mammalian cells. Proc. Natl. Acad. Sci. U S A *103*, 2677–2682.
12. Lu, S., Wang, G., Bacolla, A., Zhao, J., Spitser, S., and Vasquez, K.M. (2015). Short inverted repeats are hotspots for genetic instability: relevance to cancer genomes. Cell Rep. *10*, 1674–1680.
13. Bacolla, A., Tainer, J.A., Vasquez, K.M., and Cooper, D.N. (2016). Translocation and deletion breakpoints in cancer genomes are associated with potential non-B DNA-forming sequences. Nucleic Acids Res. *44*, 5673–5688.

14. Kamat, M.A., Bacolla, A., Cooper, D.N., and Chuzhanova, N. (2016). A role for non-B DNA forming sequences in mediating microlesions causing human inherited disease. Hum. Mutat. *37*, 65–73.
15. Georgakopoulos-Soares, I., Morganella, S., Jain, N., Hemberg, M., and Nik-Zainal, S. (2018). Noncanonical secondary structures arising from non-B DNA motifs are determinants of mutagenesis. Genome Res. *28*, 1264–1271.
16. Hänsel-Hertsch, R., Beraldi, D., Lensing, S.V., Marsico, G., Zyner, K., Parry, A., Di Antonio, M., Pike, J., Kimura, H., Narita, M., et al. (2016). G-quadruplex structures mark human regulatory chromatin. Nat. Genet. *48*, 1267–1272.
17. Bogard, N., Linder, J., Rosenberg, A.B., and Seelig, G. (2019). A deep neural network for predicting and engineering alternative polyadenylation. Cell *178*, 91–106.e23.
18. Shin, S.-I., Ham, S., Park, J., Seo, S.H., Lim, C.H., Jeon, H., Huh, J., and Roh, T.-Y. (2016). Z-DNA-forming sites identified by ChIP-Seq are associated with actively transcribed regions in the human genome. DNA Res. *23*, 477–486.
19. Gymrek, M., Willems, T., Guilmatre, A., Zeng, H., Markus, B., Georgiev, S., Daly, M.J., Price, A.L., Pritchard, J.K., Sharp, A.J., et al. (2016). Abundant contribution of short tandem repeats to gene expression variation in humans. Nat. Genet. *48*, 22–29.
20. Bacolla, A., and Wells, R.D. (2004). Non-B DNA conformations, genomic rearrangements, and human disease. J. Biol. Chem. *279*, 47411–47414.
21. Wells, R.D. (2007). Non-B DNA conformations, mutagenesis and disease. Trends Biochem. Sci. *32*, 271–278.
22. Bacolla, A., and Wells, R.D. (2009). Non-B DNA conformations as determinants of mutagenesis and human disease. Mol. Carcinog. *48*, 273–285.
23. Xie, K.T., Wang, G., Thompson, A.C., Wucherpfennig, J.I., Reimchen, T.E., MacColl, A.D.C., Schluter, D., Bell, M.A., Vasquez, K.M., and Kingsley, D.M. (2019). DNA fragility in the parallel evolution of pelvic reduction in stickleback fish. Science *363*, 81–84.
24. Buisson, R., Langenbucher, A., Bowen, D., Kwan, E.E., Benes, C.H., Zou, L., and Lawrence, M.S. (2019). Passenger hotspot mutations in cancer driven by APOBEC3A and mesoscale genomic features. Science *364*. https://doi.org/10.1126/science.aaw2872.24.
25. Siddiqui-Jain, A., Grand, C.L., Bearss, D.J., and Hurley, L.H. (2002). Direct evidence for a G-quadruplex in a promoter region and its targeting with a small molecule to repress c-MYC transcription. Proc. Natl. Acad. Sci. U S A *99*, 11593–11598.
26. Belotserkovskii, B.P., De Silva, E., Tornaletti, S., Wang, G., Vasquez, K.M., and Hanawalt, P.C. (2007). A triplex-forming sequence from the human c-MYC promoter interferes with DNA transcription. J. Biol. Chem. *282*, 32433–32441.
27. Ditlevson, J.V., Tornaletti, S., Belotserkovskii, B.P., Teijeiro, V., Wang, G., Vasquez, K.M., and Hanawalt, P.C. (2008). Inhibitory effect of a short Z-DNA forming sequence on transcription elongation by T7 RNA polymerase. Nucleic Acids Res. *36*, 3163–3170. https://doi.org/10.1093/nar/gkn136.27.
28. Kumari, S., Bugaut, A., Huppert, J.L., and Balasubramanian, S. (2007). An RNA G-quadruplex in the 5′ UTR of the NRAS proto-oncogene modulates translation. Nat. Chem. Biol. *3*, 218–221. https://doi.org/10.1038/nchembio864.28.
29. Ray, B.K., Dhar, S., Shakya, A., and Ray, A. (2011). Z-DNA-forming silencer in the first exon regulates human ADAM-12 gene expression. Proc. Natl. Acad. Sci. U S A *108*, 103–108.
30. Agarwala, P., Pandey, S., Mapa, K., and Maiti, S. (2013). The G-quadruplex augments translation in the 5′ untranslated region of transforming growth factor β2. Biochemistry *52*, 1528–1538. https://doi.org/10.1021/bi301365g.30.
31. Georgakopoulos-Soares, I., Parada, G.E., Wong, H.Y., Miska, E.A., Kwok, C.K., and Hemberg, M.. Alternative splicing modulation by G-quadruplexes. https://doi.org/10.1101/700575.31.
32. Shirude, P.S., Okumus, B., Ying, L., Ha, T., and Balasubramanian, S. (2007). Single-molecule conformational analysis of G-quadruplex formation in the promoter DNA duplex of the proto-oncogene c-kit. J. Am. Chem. Soc. *129*, 7484–7485.
33. Weinhold, N., Jacobsen, A., Schultz, N., Sander, C., and Lee, W. (2014). Genome-wide analysis of noncoding regulatory mutations in cancer. Nat. Genet. *46*, 1160–1165.
34. Song, J.H., Kang, H.-J., Luevano, L.A., Gokhale, V., Wu, K., Pandey, R., Sherry Chow, H.-H., Hurley, L.H., and Kraft, A.S. (2019). Small-molecule-targeting hairpin loop of hTERT promoter G-quadruplex induces cancer cell death. Cell Chem. Biol. *26*, 1110–1121.e4.
35. Monsen, R.C., DeLeeuw, L., Dean, W.L., Gray, R.D., Sabo, T.M., Chakravarthy, S., Chaires, J.B., and Trent, J.O. (2020). The hTERT core promoter forms three parallel G-quadruplexes. Nucleic Acids Res. *48*, 5720–5734.
36. Seenisamy, J., Rezler, E.M., Powell, T.J., Tye, D., Gokhale, V., Joshi, C.S., Siddiqui-Jain, A., and Hurley, L.H. (2004). The dynamic character of the G-quadruplex element in the c-MYC promoter and modification by TMPyP4. J. Am. Chem. Soc. *126*, 8702–8709.
37. Kaiser, C.E., Van Ert, N.A., Agrawal, P., Chawla, R., Yang, D., and Hurley, L.H. (2017). Insight into the complexity of the i-motif and G-quadruplex DNA structures formed in the KRAS promoter and subsequent drug-induced gene repression. J. Am. Chem. Soc. *139*, 8522–8536.
38. Kim, N. (2019). The interplay between G-quadruplex and transcription. Curr. Med. Chem. *26*, 2898–2917.
39. Inoue, F., Kircher, M., Martin, B., Cooper, G.M., Witten, D.M., McManus, M.T., Ahituv, N., and Shendure, J. (2017). A systematic comparison reveals substantial differences in chromosomal versus episomal encoding of enhancer activity. Genome Res. *27*, 38–52.
40. Karczewski, K.J., Francioli, L.C., Tiao, G., Cummings, B.B., Alföldi, J., Wang, Q., Collins, R.L., Laricchia, K.M., Ganna, A., Birnbaum, D.P., et al. (2020). The mutational constraint spectrum quantified from variation in 141,456 humans. Nature *581*, 434–443.
41. Bacolla, A., Jaworski, A., Larson, J.E., Jakupciak, J.P., Chuzhanova, N., Abeysinghe, S.S., O'Connell, C.D., Cooper, D.N., and Wells, R.D. (2004). Breakpoints of gross deletions coincide with non-B DNA conformations. Proc. Natl. Acad. Sci. U S A *101*, 14162–14167.
42. Zerbino, D.R., Wilder, S.P., Johnson, N., Juettemann, T., and Flicek, P.R. (2015). The ensembl regulatory build. Genome Biol. *16*, 56.
43. Frigola, J., Sabarinathan, R., Mularoni, L., Muiños, F., Gonzalez-Perez, A., and López-Bigas, N. (2017). Reduced mutation rate in exons due to differential mismatch repair. Nat. Genet. *49*, 1684–1692.
44. GTEx Consortium; Laboratory, Data Analysis &Coordinating Center (LDACC)—Analysis Working Group; Statistical Methods groups—analysis Working Group; Enhancing GTEx (eGTEx) groups; NIH Common Fund; NIH/NCI; NIH/NHGRI; NIH/NIMH; NIH/NIDA; Biospecimen Collection Source Site—NDRI; et al. (2017). Genetic effects on gene expression across human tissues. Nature *550*, 204–213.
45. Marsico, G., Chambers, V.S., Sahakyan, A.B., McCauley, P., Boutell, J.M., Antonio, M.D., and Balasubramanian, S. (2019). Whole genome experimental maps of DNA G-quadruplexes in multiple species. Nucleic Acids Res. *47*, 3862–3874.
46. Hou, Y., Li, F., Zhang, R., Li, S., Liu, H., Qin, Z.S., and Sun, X. (2019). Integrative characterization of G-Quadruplexes in the three-dimensional chromatin structure. Epigenetics *14*, 894–911.
47. Consortium, Encode Project; Dunham, I., Kundaje, A., Aldred, S.F., Collins, P.J., Davis, C.A., Doyle, F., Epstein, C.B., Frietze, S., Harrow, J., et al. (2012). An integrated encyclopedia of DNA elements in the human genome. Nature *489*, 57–74.
48. Sémon, M., Mouchiroud, D., and Duret, L. (2005). Relationship between gene expression and GC-content in mammals: statistical significance and biological relevance. Hum. Mol. Genet. *14*, 421–427.
49. Fornes, O., Castro-Mondragon, J.A., Khan, A., van der Lee, R., Zhang, X., Richmond, P.A., Modi, B.P., Correard, S., Gheorghe, M., Baranašić, D.,

et al. (2020). JASPAR 2020: update of the open-access database of transcription factor binding profiles. Nucleic Acids Res. *48*, D87–D92.

50. An, J.-Y., Lin, K., Zhu, L., Werling, D.M., Dong, S., Brand, H., Wang, H.Z., Zhao, X., Schwartz, G.B., Collins, R.L., et al. (2018). Genome-wide de novo risk score implicates promoter variation in autism spectrum disorder. Science *362*. https://doi.org/10.1126/science.aat6576.50.
51. Kwok, C.K., and Merrick, C.J. (2017). G-quadruplexes: prediction, characterization, and biological application. Trends Biotechnol. *35*, 997–1013. https://doi.org/10.1016/j.tibtech.2017.06.012.51.
52. Umar, M.I., Ji, D., Chan, C.-Y., and Kwok, C.K. (2019). G-quadruplex-based fluorescent turn-on ligands and aptamers: from development to applications. Molecules *24*. https://doi.org/10.3390/molecules24132416.52.
53. Gordon, M.G., Inoue, F., Martin, B., Schubach, M., Agarwal, V., Whalen, S., Feng, S., Zhao, J., Ashuach, T., Ziffra, R., et al. (2020). lentiMPRA and MPRAflow for high-throughput functional characterization of gene regulatory elements. Nat. Protoc. *15*, 2387–2412.
54. Kircher, M., Xiong, C., Martin, B., Schubach, M., Inoue, F., Bell, R.J.A., Costello, J.F., Shendure, J., and Ahituv, N. (2019). Saturation mutagenesis of twenty disease-associated regulatory elements at single base-pair resolution. Nat. Commun. *10*, 3583. https://doi.org/10.1038/s41467–019–11526–w.
55. Ashuach, T., Fischer, D.S., Kreimer, A., Ahituv, N., Theis, F.J., and Yosef, N. (2019). MPRAnalyze: statistical framework for massively parallel reporter assays. Genome Biol. *20*, 183. https://doi.org/10.1186/s13059–019–1787–z.
56. Brooks, T.A., and Hurley, L.H. (2010). Targeting MYC expression through G-quadruplexes. Genes Cancer *1*, 641–649. https://doi.org/10.1177/1947601910377493.56.
57. Lam, E.Y.N., Beraldi, D., Tannahill, D., and Balasubramanian, S. (2013). G-quadruplex structures are stable and detectable in human genomic DNA. Nat. Commun. *4*, 1796.
58. Armas, P., David, A., and Calcaterra, N.B. (2017). Transcriptional control by G-quadruplexes: in vivo roles and perspectives for specific intervention. Transcription *8*, 21–25.
59. Wittig, B., Wölfl, S., Dorbic, T., Vahrson, W., and Rich, A. (1992). Transcription of human c-myc in permeabilized nuclei is associated with formation of Z-DNA in three discrete regions of the gene. EMBO J. *11*, 4653–4663.
60. Maruyama, A., Mimura, J., Harada, N., and Itoh, K. (2013). Nrf2 activation is associated with Z-DNA formation in the human HO-1 promoter. Nucleic Acids Res. *41*, 5223–5234.
61. Mendoza, O., Bourdoncle, A., Boulé, J.-B., Brosh, R.M., Jr., and Mergny, J.-L. (2016). G-quadruplexes and helicases. Nucleic Acids Res. *44*, 1989–2006.
62. Sharma, S. (2011). Non-B DNA secondary structures and their resolution by RecQ helicases. J. Nucleic Acids *2011*, 724215.
63. Neidle, S. (2017). Quadruplex nucleic acids as targets for anticancer therapeutics. Nat. Rev. Chem. *1*. https://doi.org/10.1038/s41570-017-0041.63.
64. Hänsel-Hertsch, R., Di Antonio, M., and Balasubramanian, S. (2017). DNA G-quadruplexes in the human genome: detection, functions and therapeutic potential. Nat. Rev. Mol. Cell Biol. *18*, 279–284.
65. Balasubramanian, S., Hurley, L.H., and Neidle, S. (2011). Targeting G-quadruplexes in gene promoters: a novel anticancer strategy? Nat. Rev. Drug Discov. *10*, 261–275.
66. Sun, Z.-Y., Wang, X.-N., Cheng, S.-Q., Su, X.-X., and Ou, T.-M. (2019). Developing novel G-quadruplex ligands: from interaction with nucleic acids to interfering with nucleic acid–protein interaction. Molecules *24*, 396. https://doi.org/10.3390/molecules24030396.66.
67. Yuan, X., Larsson, C., and Xu, D. (2019). Mechanisms underlying the activation of TERT transcription and telomerase activity in human cancer: old actors and new players. Oncogene *38*, 6172–6183.
68. Matharu, N., and Ahituv, N. (2020). Modulating gene regulation to treat genetic disorders. Nat. Rev. Drug Discov. *19*, 757–775.
69. Varshney, D., Spiegel, J., Zyner, K., Tannahill, D., and Balasubramanian, S. (2020). The regulation and functions of DNA and RNA G-quadruplexes. Nat. Rev. Mol. Cell Biol. *21*, 459–474.
70. Beaudoin, J.-D., and Perreault, J.-P. (2013). Exploring mRNA 3′-UTR G-quadruplexes: evidence of roles in both alternative polyadenylation and mRNA shortening. Nucleic Acids Res. *41*, 5898–5911. https://doi.org/10.1093/nar/gkt265.70.
71. Yang, F., Sun, X., Wang, L., Li, Q., Guan, A., Shen, G., and Tang, Y. (2017). Selective recognition of c-myc promoter G-quadruplex and down-regulation of oncogene c-myc transcription in human cancer cells by 3,8a-disubstituted indolizinone. RSC Adv. *7*, 51965–51969. https://doi.org/10.1039/c7ra09870g.71.
72. Nagesh, N., Buscaglia, R., Dettler, J.M., and Lewis, E.A. (2010). Studies on the site and mode of TMPyP4 interactions with Bcl-2 promoter sequence G-quadruplexes. Biophys. J. *98*, 2628–2633.
73. Agrawal, P., Lin, C., Mathad, R.I., Carver, M., and Yang, D. (2014). The major G-quadruplex formed in the human BCL-2 proximal promoter adopts a parallel structure with a 13-nt loop in K solution. J. Am. Chem. Soc. *136*, 1750–1753. https://doi.org/10.1021/ja4118945.73.
74. Phan, A.T., Kuryavyi, V., Burge, S., Neidle, S., and Patel, D.J. (2007). Structure of an unprecedented G-quadruplex scaffold in the human c-kit promoter. J. Am. Chem. Soc. *129*, 4386–4392.
75. Crawford, D.C., Acuña, J.M., and Sherman, S.L. (2001). FMR1 and the fragile X syndrome: human genome epidemiology review. Genet. Med. *3*, 359–371.
76. Khateb, S., Weisman-Shomer, P., Hershco-Shani, I., Ludwig, A.L., and Fry, M. (2007). The tetraplex (CGG)n destabilizing proteins hnRNP A2 and CBF-A enhance the in vivo translation of fragile X premutation mRNA. Nucleic Acids Res. *35*, 5775–5788. https://doi.org/10.1093/nar/gkm636.76.
77. Cogoi, S., and Xodo, L.E. (2006). G-quadruplex formation within the promoter of the KRAS proto-oncogene and its effect on transcription. Nucleic Acids Res. *34*, 2536–2549.
78. Cogoi, S., Paramasivam, M., Spolaore, B., and Xodo, L.E. (2008). Structural polymorphism within a regulatory element of the human KRAS promoter: formation of G4-DNA recognized by nuclear proteins. Nucleic Acids Res. *36*, 3765–3780.
79. Zhang, H., Huang, T., Hong, Y., Yang, W., Zhang, X., Luo, H., Xu, H., and Wang, X. (2018). The retromer complex and sorting nexins in neurodegenerative diseases. Front. Aging Neurosci. *10*, 79.
80. Agrawal, P., Hatzakis, E., Guo, K., Carver, M., and Yang, D. (2013). Solution structure of the major G-quadruplex formed in the human VEGF promoter in K : insights into loop interactions of the parallel G-quadruplexes. Nucleic Acids Res. *41*, 10584–10592. https://doi.org/10.1093/nar/gkt784.80.
81. Schlag, K., Steinhilber, D., Karas, M., and Sorg, B.L. (2020). Analysis of proximal ALOX5 promoter binding proteins by quantitative proteomics. FEBS J. https://doi.org/10.1111/febs.15259.81.
82. Cer, R.Z., Donohue, D.E., Mudunuri, U.S., Temiz, N.A., Loss, M.A., Starner, N.J., Halusa, G.N., Volfovsky, N., Yi, M., Luke, B.T., et al. (2013). Non-B DB v2.0: a database of predicted non-B DNA-forming motifs and its associated tools. Nucleic Acids Res. *41*, D94–D100.
83. Hänsel-Hertsch, R., Spiegel, J., Marsico, G., Tannahill, D., and Balasubramanian, S. (2018). Genome-wide mapping of endogenous G-quadruplex DNA structures by chromatin immunoprecipitation and high-throughput sequencing. Nat. Protoc. *13*, 551–564.
84. Georgakopoulos-Soares, I., Jain, N., Gray, J.M., and Hemberg, M. (2017). MPRAnator: a web-based tool for the design of massively parallel reporter assay experiments. Bioinformatics *33*, 137–138.
85. Quinlan, A.R., and Hall, I.M. (2010). BEDTools: a flexible suite of utilities for comparing genomic features. Bioinformatics *26*, 841–842.
86. Li, H. (2013). Aligning sequence reads, clone sequences and assembly contigs with BWA-MEM. Preprint at arXiv, 1303.3997v2.90.

87. Grant, C.E., Bailey, T.L., and Noble, W.S. (2011). FIMO: scanning for occurrences of a given motif. Bioinformatics *27*, 1017–1018.

88. Yu, G., Wang, L.-G., Han, Y., and He, Q.-Y. (2012). clusterProfiler: an R package for comparing biological themes among gene clusters. OMICS *16*, 284–287. https://doi.org/10.1089/omi.2011.0118.87.

89. Jain, A., and Tuteja, G. (2019). TissueEnrich: tissue-specific gene enrichment analysis. Bioinformatics *35*, 1966–1967.

90. Virtanen, P., Gommers, R., Oliphant, T.E., Haberland, M., Reddy, T., Cournapeau, D., Burovski, E., Peterson, P., Weckesser, W., Bright, J., et al. (2020). SciPy 1.0: fundamental algorithms for scientific computing in Python. Nat. Methods *17*, 261–272.

91. Inoue, F., Kreimer, A., Ashuach, T., Ahituv, N., and Yosef, N. (2019). Identification and massively parallel characterization of regulatory elements driving neural induction. Cell Stem Cell *25*, 713–727.e10.

92. Chan, K.L., Peng, B., Umar, M.I., Chan, C.-Y., Sahakyan, A.B., Le, M.T.N., and Kwok, C.K. (2018). Structural analysis reveals the formation and role of RNA G-quadruplex structures in human mature microRNAs. Chem. Commun. *54*, 10878–10881.

93. Chan, C.-Y., Umar, M.I., and Kwok, C.K. (2019). Spectroscopic analysis reveals the effect of a single nucleotide bulge on G-quadruplex structures. Chem. Commun. *55*, 2616–2619.

STAR★METHODS

KEY RESOURCES TABLE

REAGENT or RESOURCE	SOURCE	IDENTIFIER
**Bacterial and virus strains**		
ElectroMAX Stbl4	ThermoFisher Scientific	Cat#11635018
**Chemicals, peptides, and recombinant proteins**		
Polybrene	Sigma	Cat#TR-1003-G
Lithium Hydroxide	Acros Organics	Cat# 413325000
Cacodylic Acid	Acros Organics	Cat# 318150100
Lithium Chloride	Sigma	t# L7026-1L
Potassium Chloride	Thermo Fisher	Cat# J/2892/15
N-Methyl Mesoporphyrin IX (NMM)	Frontier Specialty Chemicals	Cat# NMM580-5mg
Dimethyl sulfoxide	J&K Scientific	Cat# 292271
**Critical commercial assays**		
Lenti-Pac HIV Expression Packaging Kit	Genecopoeia	Cat#LT001
Lenti-X concentrator	Takara	Cat#631231
Dual-Luciferase Reporter Assay System	Promega	Cat#E1910
NEBuilder HiFi DNA Assembly Master Mix	New England Biolabs	Cat#E2621S
NEBNext High-Fidelity 2X PCR Master Mix	New England Biolabs	Cat#M0541S
QIAquick gel extraction kit	Qiagen	Cat#28704
MinElute Reaction Cleanup Kit	Qiagen	Cat#28204
Allprep DNA/RNA mini kit	Qiagen	Cat#80204
Oligotex mRNA mini kit	Qiagen	Cat#70022
SuperScriptII	Life Technologies	Cat#18064-071
**Deposited data**		
ENCODE MPRA for K562 and HEPG2 cell lines	Consortium, Encode Project[47]	https://www.encodeproject.org/
HEK-293T and K562 MPRPA	This paper	PRJNA763774
NPC MPRA	This paper	PRJNA763774
Non-B DNA motif maps	Cer et al.[82]	https://nonb-abcc.ncifcrf.gov/
Ensembl Regulatory Build	Zerbino et al.[42]	https://m.ensembl.org/info/genome/funcgen/regulatory_build.html
G4-seq and G4-ChIP-seq data	Marsico et al.,[45] Hänsel-Hertsch et al.[83]	GSE63874; GSE107690
eQTLs from GTEx consortium	GTEx Consortium[44]	https://gtexportal.org/home/
Population variants	Karczewski et al.[40]	https://gnomad.broadinstitute.org/
Transcription factor binding profiles	Fornes et al.[49]	https://jaspar.genereg.net/
**Experimental models: Cell lines**		
293T	ATCC	Cat#CRL-3216, RRID:CVCL_0063
K562	ATCC	Cat#CCL-243, RRID:CVCL_0004
MCF-7	ATCC	Cat#HTB-22, RRID:CVCL_0031
IMR-90	ATCC	Cat#CCL-186, RRID:CVCL_0347
**Software and algorithms**		
Code associated with this manuscript	This paper	https://doi.org/10.5281/zenodo.6098968
MPRAnator	MPRAnator et al.[84]	https://genomegeek.com/
BEDTools utilities v2.21.0	Quinlan et al.[85]	https://github.com/arq5x/bedtools2
BWA MEM	Li[86]	http://bio-bwa.sourceforge.net/
FIMO	Grant et al.[87]	https://meme-suite.org/meme/doc/fimo.html

(Continued on next page)

Continued

REAGENT or RESOURCE	SOURCE	IDENTIFIER
MPRAflow	Gordon et al.[53]	https://github.com/shendurelab/MPRAflow
clusterProfiler	Yu et al.[88]	https://bioconductor.org/packages/release/bioc/html/clusterProfiler.html
TissueEnrich	Jain et al.[89]	https://bioconductor.org/packages/release/bioc/html/TissueEnrich.html
SciPy	Virtanen et al.[90]	https://scipy.org/
FluorEssence™	HORIBA	https://www.horiba.com/int/products/detail/action/show/Product/fluoressence-1378/
Spectra Manager™	JASCO	https://jascoinc.com/products/spectroscopy/circular-dichroism/software/spectra-manager/
Cary WinUV Software	Agilent	https://www.agilent.com/en/product/molecular-spectroscopy/uv-vis-uv-vis-nir-spectroscopy/uv-vis-uv-vis-nir-software/cary-winuv-software

RESOURCE AVAILABILITY

Lead contact

Further information and requests for resources should be directed to and will be fulfilled by the lead contact, Nadav Ahituv (nadav.ahituv@ucsf.edu).

Materials availability

This study did not generate new unique reagents.

Data and code availability

The MPRA data for the NPC cell line targeting autism-related loci and the MPRA data for the non-B DNA associated loci in HEK-293T and K562 cell lines are deposited in NCBI BioProject with accession number PRJNA763774. The MPRA data for HEPG2 and K562 cell lines (Figure 3) have been deposited in the ENCODE portal with IDs ENCSR463IRX and ENCSR460LZI.

All original code and data tables to perform the analyses can be found on the GitHub page (https://github.com/IliasGeoSo/High_Throughput_MPRA_Non_B_DNA) and are publicly available.

Any additional information required to reanalyze the data reported in this paper is available from the lead contact upon request.

EXPERIMENTAL MODEL AND SUBJECT DETAILS

Cell culturing was performed for HEK293T (RRID CVCL_0063), K562 (RRID CVCL_0004), MCF-7 (RRID: CVCL_0031) and IMR-90 (RRID: CVCL_0347) cell lines. Human HEK293T embryonic kidney cells were cultured in Dulbecco's modified Eagle's medium (DMEM, Sigma) supplemented with 10% FBS and 2mmol/ L L-glutamine. Human K562 erythroleukemia cells were cultured in Iscove's modified Dulbecco's medium (IMDM, Sigma-Aldrich) supplemented with 10% FBS. Human MCF-7 breast cancer cells were cultured in Eagle's minimal essential medium (MEM, Sigma-Aldrich) supplemented with 10% FBS, 10μg/ml insulin, 1mM sodium pyruvate and 0.1 mM non-essential amino acids. Human IMR-90 fibroblasts were cultured in MEM supplemented with 10% FBS and 0.1 mM non-essential amino acids. Neural progenitor cells were differentiated from H1 hESCs following the dual-Smad inhibition protocol as described in [91]. All cell lines were grown at 37°C and 5% CO_2.

METHOD DETAILS

Genomic elements

Gene annotation from Ensembl was followed throughout. Genic regions were separated into introns, coding exons, 5′ UTRs and 3′ UTRs, 1kb upstream of the TSS, 1kb downstream of the TES based on UCSC Table Browser using browser extensive data selection files. BEDTools utilities v2.21.0 were used to manipulate genomic files and intervals.[85]

Ensembl regulatory build

Regulatory features were derived from the Ensembl regulatory build for twelve commonly used cell lines across human tissues, namely A549, HMEC, HUVEC, IMR-90, K562, HepG2, HSMM, MCF-7, NHEK, H1-ESC, GM12878 and HCT116.[42] The enrichment in Figures 2A and 2C were calculated from the median enrichment across the cell lines.

Non-B DNA motif identification

The genome-wide analysis of non-B DNA motifs was performed using the positions derived from [82]. Custom scripts were developed in Python to identify STRs, DRs, IRs, MRs, Z-DNA and G4s across the MPRA sequences. Consensus G4 motifs were derived using the regular expression ([gG]{3,}\w{1,7}){3,}[gG]{3,}. IR, DR and MRs with arm lengths of 10bp and spacer sequences of up to 4bps were identified, unless otherwise defined in the particular figure. Z-DNA sequences were defined as alternating purine-pyrimidine tracts of at least 10bp length. The subset of MRs that have high AG content (>90%) and which are more likely to form H-DNA structures. Here, H-DNA motifs were defined as the subset of MRs that have a high (>90%) AG content, arm lengths of >=10bp and spacer size of less than 8bp. Custom scripts were developed in Python to identify the size and positions of the non-B DNA motif sub-components. For DR motif identification, the STR repeat threshold within the arm was set to 80%, in order to separate them from STR motifs. Enrichment of mutations at non-B DNA motifs was estimated as described in [15].

G4-seq and G4 ChIP-seq maps

G4-seq BedGraph data were derived from GEO accession code GSE63874 for the human genome, in two conditions, PDS and K^+ treatments.[45] G4 ChIP-seq data were derived from GEO accession code GSE107690 for K562 cell line.[83]

G4 motifs were oriented as template and non-template based on their orientation relative to gene direction, across genic regions. Strand orientation of G4 motifs at G4-seq and G4 ChIP-seq peaks was performed by subsetting the strand of G4 motifs overlapping the peaks.

Transcription factor binding site maps

Position frequency matrices (PFMs) of transcription factors were derived from JASPAR (release 2020)[49] for the non-redundant CORE collection (http://jaspar.genereg.net/download/CORE/JASPAR2020_CORE_vertebrates_non-redundant_pfms_meme.zip) and motif scanning was performed with FIMO.[87]

Population variant analysis

Nucleotide variants, indels as well as structural variants were derived from the GnomAD project for whole genome sequenced datasets.[40] Only variants with the filter flag PASS were analyzed.

eQTL analysis

eQTLs were derived from the GTEx consortium[44] and analyzed with the commands "intersect" and "closest" from BEDTools to investigate their intersection and distribution patterns with motifs from each non-B DNA category as well as with G4-seq and G4 ChIP-seq peaks.

Gene set enrichment analysis

For each type of non-B DNA motif, we extracted a group of genes that contain a non-B DNA motif within a 200 bp upstream window from their TSS and these were used to perform gene set enrichment analyses. GO analyses were performed using clusterProfiler,[88] where GO terms with at least 20 genes and gene ratio greater than 0.01 for at least one of the non-B DNA sets were considered. For visualization purposes, we only displayed a maximum of 10 GO terms with the highest gene ratio per non-B DNA set. Finally, we calculated the enrichment of each non-B DNA group across sets of tissue-specific genes using TissueEnrich[89] using default arguments.

Luciferase assay

Candidate promoters of 200 bp were PCR amplified using AccuPrime™ GC-Rich DNA Polymerase (ThermoFisher Scientific 12337016) and cloned into the pLSmP-Luciferase vector after digestion with SbfI and AgeI restriction enzymes (remove minimal promoter). Primers with 20bp homology to the vector cloning site were designed and PCR products were assembled to the lentiviral vector using NEBuilder® HiFi DNA Assembly Master Mix (E2621S). Lentiviruses were produced using Lenti-Pac HIV Expression Packaging Kit (Genecopoeia, LT001) according to manufacturer's instructions. Small scale viral productions on HEK293T cells (2x800,000 cells seeded on a p6 well 24h prior to transfection; virus-containing culture media was collected 48h post-transfection and was used to infect desired cells) were performed of all different constructs to test luciferase activity in four different cell lines (MCF-7, IMR-90, K562, HEK293T). 50,000 cells were seeded on 96-well plates in a volume of 50 μL and another 50 μL of virus-containing medium was added in order to transduce them. Luminescence was measured 24h or 48h post-infection using Dual-Luciferase® Reporter Assay System (Promega, E1910).

lentiMPRA of promoters

Each of the sequences was synthesized on a 7,500-feature microarray (Agilent OLS; 15 bp primer + 200 bp promoter + 15 bp primer = 230 mers). For the G4s that we studied across these genes, all selected loci overlapped G4-seq or G4 ChIP-seq peaks. lentiMPRA was performed as described previously with modifications.[53]

In brief, PCR amplification of OLS library was performed using NEBNext® High-Fidelity 2X PCR Master Mix (New England Biolabs, M0541S)(4x50 μl reactions using 20 ng of template OLS library and primers L1.Amp.F and L1.Amp.R; PCR program: 95°C, 2 min; (95°C, 15 sec; 65°C, 20 sec; 72°C, 1 min) x12 cycles; 72°C, 5min). Barcodes were added by PCR in the library amplification step in the 5′ UTR of the GFP gene. This PCR 5′-tagging strategy allowed us to eliminate the confounding effect that lentiviral genome recombination might have on 3′-tagged libraries. Additionally, tagging barcodes in the PCR amplification step via primers harboring degenerate nucleotides enabled us to assay larger promoter sequences (200 bp instead of 171 bp of previous MPRA designs) and the cost-effective use of an oligonucleotide library 100 times smaller in size to obtain 100 barcodes per promoter (we ordered 7,500 different sequences instead of 750,000). 20 μg of lentiviral vector (pLSmP-GFP) were digested with *Sbf*I and *Age*I restriction enzymes. Linearized vector and PCR products were run on a 1% agarose gel and purified using QIAquick gel extraction kit (QIAGEN, 28704). 5x20 μl ligations containing 1:10 molar ratio between vector and inserts were performed using NEBuilder® HiFi DNA Assembly Master Mix (E2621S). Ligations were pooled and purified using MinElute Reaction Cleanup Kit (QIAGEN 28204) and electroporated into ElectroMAX™ Stbl4™ Competent Cells (ThermoFisher Scientific 11635018). 50 μl of electrocompetent bacteria and 60 ng of DNA were used per reaction in a 0.1 cm cuvette (Program: 1.2kV; 200 ohms; 25 μF; 1 pulse). 1:1,000 and 1:10,000 dilutions were seeded on LB plates with ampicillin in order to estimate the number of clones. Approximately 800,000 different clones were obtained and, thus, the complexity of the plasmid library with an estimated of 100 barcodes per insert. Insert-barcode fragment was amplified from the plasmid library and sequenced using NextSeq PE150 for the insert-barcode association.

Lentiviral particles were produced from the plasmid library as in the luciferase assay but scaling the process to 6x150 mm plates. In summary, $6x10^6$ HEK293T cells were seeded per plate 48 hour before transfection, 5 μg of plasmid library and 5 μg of HIV packaging mix were co-transfected using 30 μl of EndoFectin. Media were collected 48h post-transfection and lentiviral particles were concentrated using Lenti-X™ Concentrator (Takara 631231). Lentiviral library was tested in a small scale experiment with HEK293T and K562 cell lines in order to titrate the number of desired integrations. Three million HEK293T and 4 million K562 cells were infected with the library with the multiplicity of infection (MOI) of 400 and 40, respectively, as calculated in small scale titration experiments. In order to improve infection, polybrene was added together with the lentiviral library at a final concentration of 8 μg/ml. After three days of culture, barcoded DNA and RNA were extracted from the cells using Allprep DNA/RNA mini kit (QIAGEN 80204). mRNA was purified using Oligotex mRNA mini kit (QIAGEN 70022), and reverse-transcribed using SuperScriptII (Life Technologies, 18064-071), according to manufacturer's instructions. Barcodes were amplified and sequenced using NextSeq PE15, as described previously.[53] We performed three independent replicates of infection for each cell line.

MPRA analysis pipeline

The design of MPRA sequences was performed with algorithms adjusted from [84]. For barcode insert mapping and filtering, we called a consensus sequence from the paired-end reads associating with barcode sequence from the index read. We aligned all consensus sequences back to all designed sequences (inserts) using BWA MEM (version 0.7.17-r1188).[86] As many of the designed sequences are either only 1bp mutation from each other, or the inverted orientation, we use CIGAR string with perfect sequence match and 0 mismatches as a strict filter. For RNA/DNA barcode counting and ratio normalization, RNA and DNA barcodes for each of three replicates were sequenced on an Illumina NextSeq instrument, UMI is used to remove PCR duplicates and the inserts with associated barcode counts lower than 3 are removed. Evaluating the effect of GC-content in the contribution to expression across the MPRA was performed by fitting a linear model and subtracting from each sequence the expected score due to GC-content.

NMM ligand enhanced fluorescence

Experiments were carried out as previously reported with slight modification.[92] Sample solutions of 100 μL total volume were prepared containing 1 μM DNA, 10 mM lithium cacodylate (LiCac) buffer (pH 7.0), 150 mM LiCl or KCl solution and 1 μM NMM ligand. HORIBA FluoroMax-4 Fluorometer was used to measure the fluorescence spectra. Before sample measurement, samples were first prepared without ligand and heated for denaturation at 95°C for 3 minutes followed by cooling down for 15 minutes by placing the sample solution at room temperature so as to undergo renaturation. The samples were then transferred into a quartz cuvette which had a path length of 1-cm and excited at 394 nm. The range from 550 to 750 nm of emission spectra were needed. All data were measured at 25°C in every 2 nm and the exit and entrance slit widths were 5 nm. The enhanced fluorescence spectra of samples in the absence of ligand were used for normalization. All of the above calculations were analyzed in Microsoft Excel.

Circular dichroism (CD) spectroscopy

Experiments were carried out as previously reported with slight modification.[93] Jasco J-1500 CD spectrophotometer was used to carry out the CD spectroscopy. A total of 2 mL sample solution was contained with a quartz cuvette which had a path length of 1-cm. Sample reactions consisting of 5 μM DNA, 150 mM KCl or LiCl and 10 mM LiCac (pH 7.0) were prepared. Then mixed thoroughly and denatured the sample solution for 5 minutes at 95°C and then incubated for 15 minutes by placing the sample solution at room temperature to undergo renaturation. All samples were measured at 25°C in a range from 220 to 310 nm. The spectra were

needed every 1 nm. The time for responding was 0.5 s/nm and all of the spectra stated were 2 scans in average. By normalizing the data collected, the molar residue ellipticity was obtained and then smoothed over 5 nm. Spectra Manager Suite (Jasco Software) was used to analyze the collected data.

Thermal melting monitored by UV spectroscopy

Experiments were carried out as previously reported with slight modification.[93] Sample reactions of 2 mL consisting of 5 μM DNA (except for concentration dependent melting that ranged from 1 – 10 μM), 150 mM KCl and 10 mM LiCac (pH 7.0) were prepared. Samples were then mixed completely and heated for 3 minutes at 95°C for DNA denaturation and followed by renaturation for 15 minutes by placing the sample solution at room temperature. Samples were then transferred into a quartz cuvette which had a path length of 1-cm then sealed with 2 layers of Teflon tape in order to lower the chance of evaporation of the sample when the measurement reached high temperature. Measurements were conducted using Agilent Cary 100 UV-Vis Spectrophotometer with sample block initially set at 20°C for 5 minutes.

The samples were measured from 20 to 95°C (forward scan) with a 0.5°C/min temperature increment rate. There was a reverse scan measurement (95 to 20°C) that also had a 0.5°C/min increment rate after holding for 5 minutes at 95°C. At 295 nm (or 260nm for the B-DNA oligonucleotide), both of the forward and reverse scans were recorded for the folding and unfolding transitions.

The collected data were deducted by the blanked solutions which had the identical concentrations of the KCl and LiCac buffer (pH 7.0) only. The data's first derivatives were obtained by smoothing the data over 11 nm where all the processes and results were marked in Microsoft Excel. By taking average of the melting temperatures in both of the reversed and forward measurements, the final melting temperature was determined.

Intrinsic fluorescence spectroscopy

Experiments were carried out as previously reported with slight modification.[93] Samples were prepared as done for UV-melting and CD-spectroscopy. HORIBA FluoroMax-4 Fluorometer was used to measure the intrinsic fluorescence spectra. After denaturation and renaturation of samples, the samples were transferred into a quartz cuvette which had a path length of 1-cm and excited at 260 nm. The range from 300 to 500 nm of the emission spectra were needed. All data were measured at 25°C of every 2 nm and the exit and entrance slit widths were 5 nm. The collected data were smoothed over 5 nm using Microsoft Excel.

QUANTIFICATION AND STATISTICAL ANALYSIS

Population variant analysis

For SNP variants, simulated controls were generated within 10kb from the original variant, controlling for trinucleotide content. To achieve this, the base-pair at the randomly selected simulated position, within 10kb from the original mutation, and both the 5' and the 3' adjacent base-pairs had to match those at the mutated sites, and the mutation and simulation sites had to be different from one another. In addition, in the simulations regions of the human genome for which mutation calling by GnomAD was not performed were excluded. For indels, we generated simulated indels within 10kb of the original indel site, correcting for indel length and with local GC content at a 100bp window each side of the indel site within 2.5% difference from the original. For structural variants, we simulated an equal number of breakpoints at random locations within 10kb of the original breakpoints, correcting for local GC content, with 2.5% maximum difference from the original GC content. Statistical significance was estimated with non-parametric Mann-Whitney U tests in Python using the SciPy library.[90] Across regulatory elements, z-scores were calculated from the density of mutations at non-B DNA motifs at that element, relative to the mean mutational density at that element, divided by the standard deviation.

Transcription factor binding

PFMs were used to identify transcription factor binding sites with FIMO,[87] which was used with background model the nucleotide frequencies across the human genome and requiring a minimum p-value $<10-6$.

MPRA analysis

Statistical significance of expression difference between sequences with and without a non-B DNA motif was estimated with Mann-Whitney U tests with Bonferroni correction in Python using the SciPy library.[90]

Cell Genomics, Volume *2*

Supplemental information

High-throughput characterization of the role of non-B DNA motifs on promoter function

Ilias Georgakopoulos-Soares, Jesus Victorino, Guillermo E. Parada, Vikram Agarwal, Jingjing Zhao, Hei Yuen Wong, Mubarak Ishaq Umar, Orry Elor, Allan Muhwezi, Joon-Yong An, Stephan J. Sanders, Chun Kit Kwok, Fumitaka Inoue, Martin Hemberg, and Nadav Ahituv

Supplemental Information

Table S1, related to figure 4. Selected putative G-quadruplexes for validation experiments. G-runs are marked in bold. Coordinates shown in hg19.

Gene Name	Sequence (5′-3′)	MPRA Coordinates
KCNB1	**GGG**T**GGGG**CCTCCC**GGG**CTCCA**GGGG**	chr20:48099339-48099539
DFF3	**GGGG**C**GGGGG**CCGC**GGG**CTC**GGGGG** CGC**GGGG**	chr14:73360145-73360345
DFF3	**GGGGG**AAGCAGC**GGG**TCCC**GGG**CGT GCT**GGGG**	chr14:73360145-73360345
GALNT9	**GGG**CT**GGGGG**T**GGGGG**CAGCC**GGGG**	chr12:132906730-132906930
AKT1	**GGG**CCGT**GGGG**CTCCCC**GGG**CGCT**GG G**	chr14:105261369-105261569
PRSS27	**GGGG**CGGCAC**GGGG**C**GGGG**CTGCGC C**GGGGG**AA**GGG**	chr16:2827279-2827479
CNOT6	**GGGGG**TAA**GGGGG**C**GGGG**CCT**GGG**	chr5:179921169-179921369
SERTAD 2	**GGGG**AC**GGG**C**GGGG**TAA**GGGGG**	chr2:64978074-64978274
ARF5	**GGGGG**C**GGGG**CCCGGAC**GGGGG**C**GG G**	chr7:127228267-127228467
TNX2	**GGG**CGAAGGC**GGGGG**C**GGGG**C**GGGG**	chr22:36878177-36878377

Table S2, related to figure 5. Selected gene coordinates for the MPRA experiment.

The promoters of ten gene targets were tiled. Marked is the non-B DNA motif that has been analyzed in previous studies. The human genome reference strand is defined as "Reference" and reference reverse complement strand as "Complement".

Gene Name	Diseases associated	Strand	Coordinates MPRA	TSS position	Non-B DNA Motif	References
C-MYC	Cancer	Reference	chr8:128748165-128748364	chr8:128748315	DR, **G4**, H-DNA, IR	[25], [71], [56]
BCL-2	Cancer	Complement	chr18:60987335-60987534	chr18: 60987360	**G4**, STR	[72], [73]
ADAM-12	Cancer	Complement	chr10:128076900-128077099	chr10:128077024	IR, **Z-DNA**	[18]
C-KIT	Cancer	Reference	chr4:55523900-55524099	chr4:55524084	**G4**, IR, Z-DNA	[74]
FMR1	Fragile X Syndrome	Reference	chrX:146993440-146993639	chrX:146993469	**STR**	[75], [76]
KRAS	Cancer	Complement	chr12:25403860-25404059	chr12:25403870	**G4**, STR	[77], [78]
SNX-12	neurodegenerative diseases	Complement	chrX:70288232-70288431	chrX:70288272	STR, **Z-DNA**	[79], [18]
VEGF-12	Cancer	Reference	chr6:43737771-43737970	chr6:43737921	**G4**, IR, STR	[80]
SRSF-6	Cancer	Reference	chr20:42086418-42086617	chr20:42086568	IR, **Z-DNA**	[18]
ALOX-5	Cancer	Reference	chr10:45869511-45869710	chr10:45869661	**G4,** STR	[81]

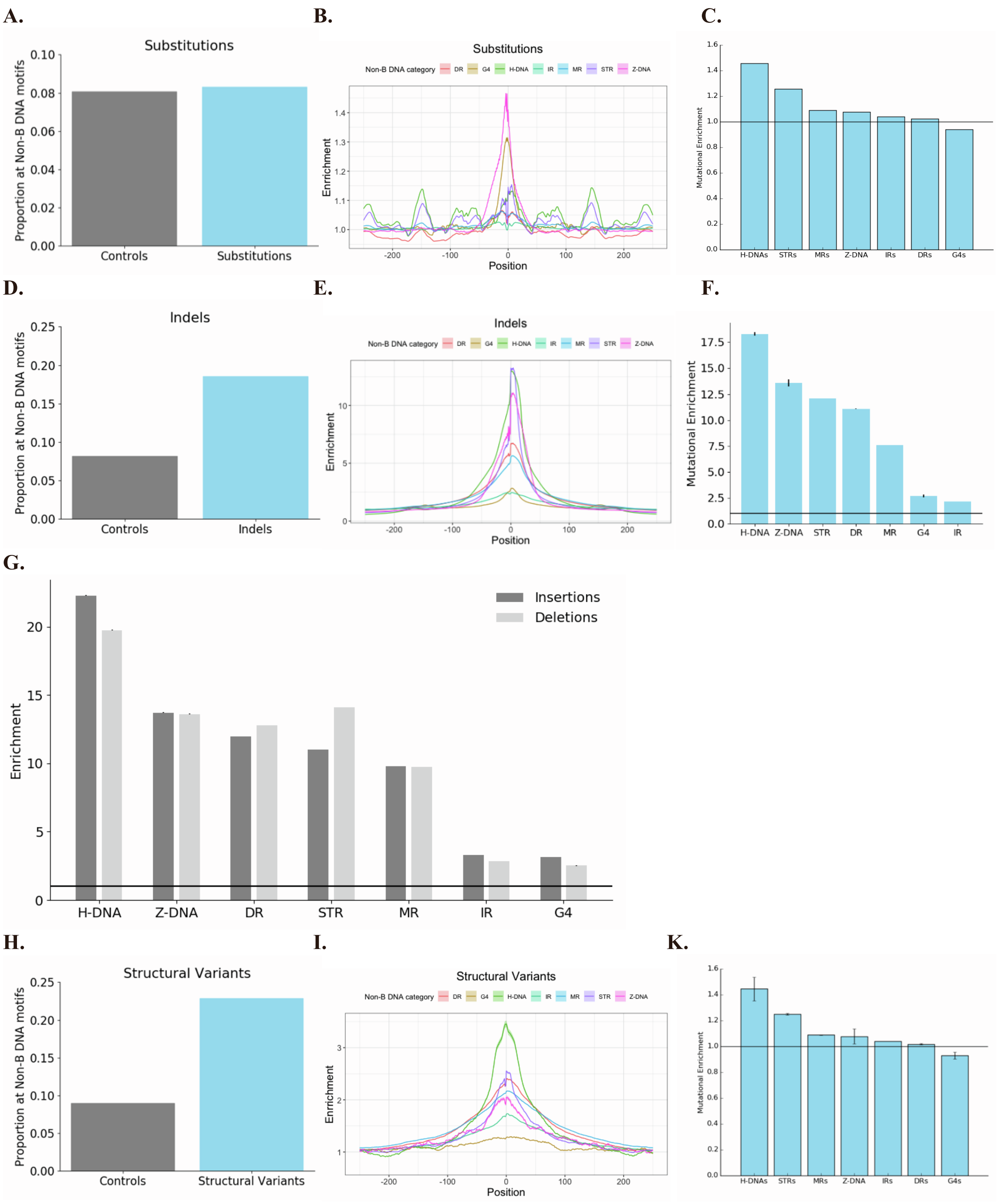

Figure S1, related to figure 1. Mutational density is locally increased at non-B DNA motifs.

a. Distribution of non-B DNA motifs relative to SNP positioning, uncorrected for trinucleotide context.

b. Proportion of SNP variants and simulated controls overlapping non-B DNA motifs. Mann-Whitney U test p-value <0.0001.

c. Enrichment of SNPs at non-B DNA motifs, relative to controls. Error bars indicate standard error from bootstrapping.

d. Distribution of non-B DNA motifs relative to indel variants positioning, uncorrected for trinucleotide context.

e. Proportion of indel variants and simulated controls overlapping non-B DNA motifs. Mann-Whitney U test p-value <0.0001.

f. Enrichment of indel variants at non-B DNA motifs, relative to controls. Error bars indicate standard error from bootstrapping.

g. Proportion of insertions and deletions overlapping each non-B DNA motif category. Error bars indicate standard error from bootstrapping.

h. Proportion of structural variants and simulated controls overlapping non-B DNA motifs. Mann-Whitney U test p-value <0.0001.

i. Proportion of structural variant breakpoints and simulated controls overlapping non-B DNA motifs. Mann-Whitney U test p-value <0.0001.

k. Enrichment of structural variant breakpoints at non-B DNA motifs, relative to controls. Error bars indicate standard error from bootstrapping. For certain barplots in this figure the standard error is smaller than the resolution of the image.

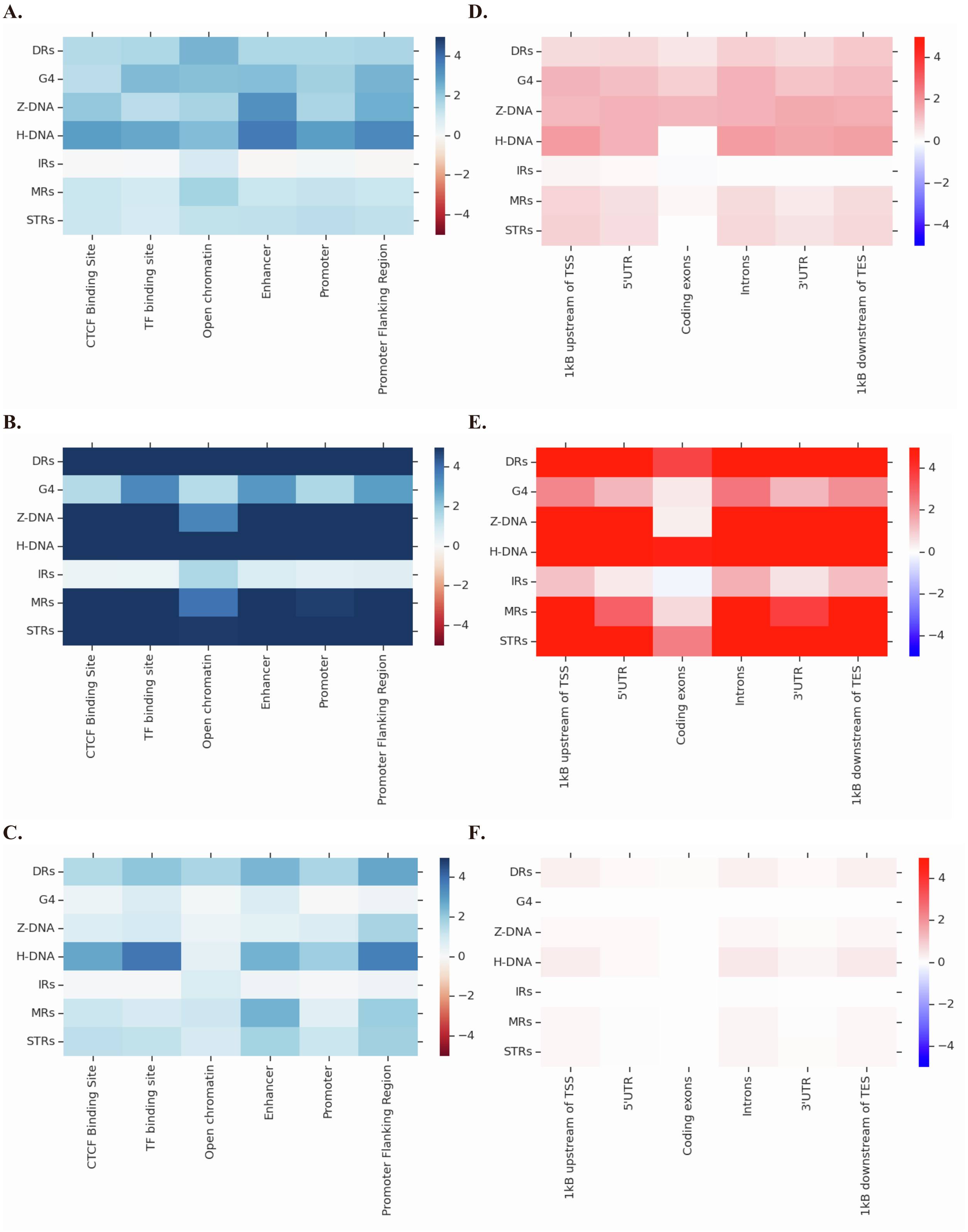

Figure S2, related to figure 1. Mutational distribution at non-B DNA motifs across regulatory elements and genic compartments.

a. Z-scores of the relationship between SNP variant frequency at Ensembl regulatory elements and non-B DNA motifs.
b. Z-scores of the relationship between indel variant frequency at Ensembl regulatory elements and non-B DNA motifs.
c. Z-scores of the relationship between structural variant frequency at Ensembl regulatory elements and non-B DNA motifs.
d. Z-scores of the relationship between SNP variant frequency at genic regions and non-B DNA motifs.
e. Z-scores of the relationship between indel variant frequency at genic regions and non-B DNA motifs.
f. Z-scores of the relationship between structural variant frequency at genic regions and non-B DNA motifs.

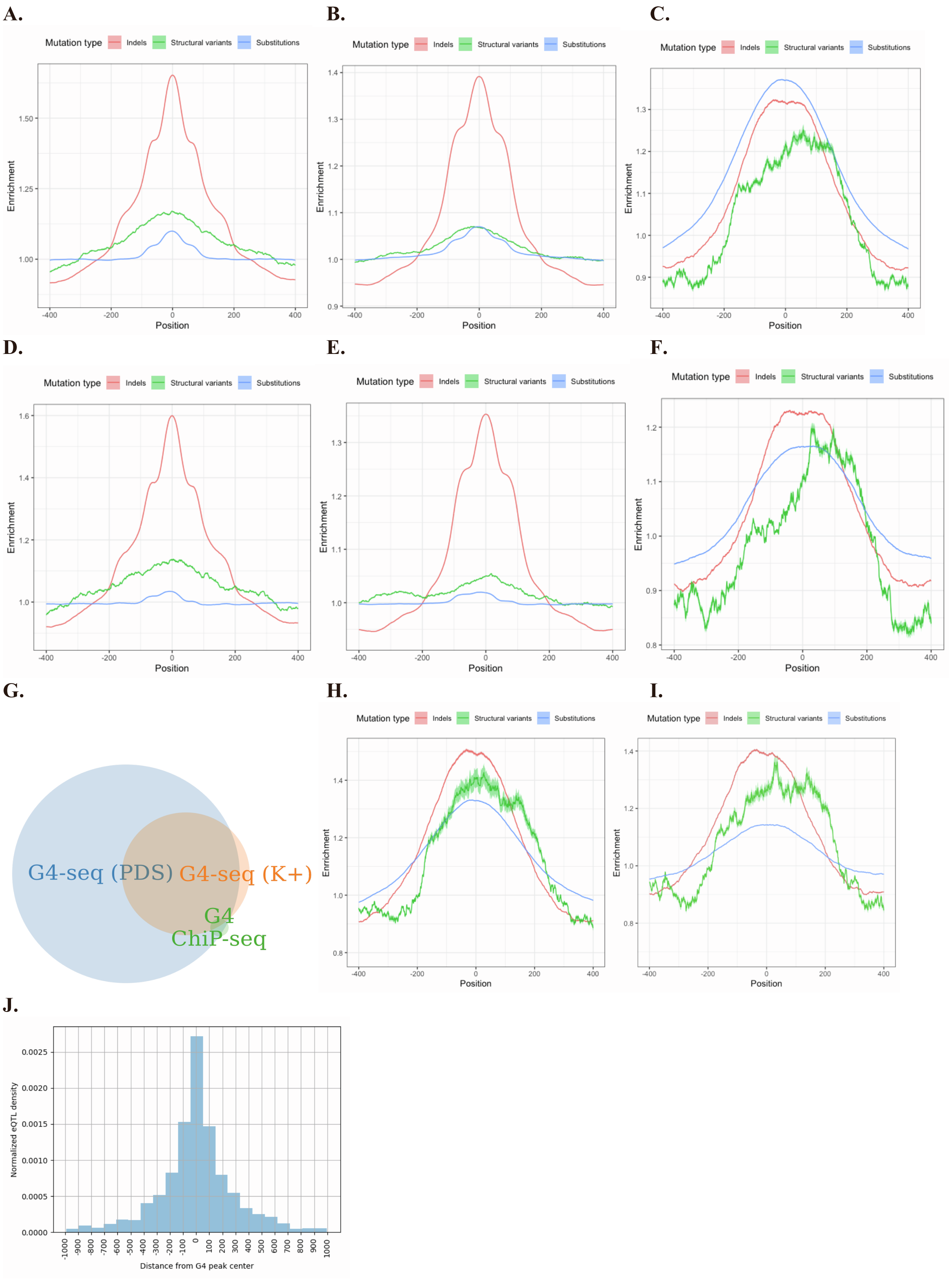
A.
B.
C.
D.
E.
F.
G.
H.
I.
J.
Mutation type
Indels
Structural variants
Substitutions
Enrichment
Position
G4-seq (PDS)
G4-seq (K+)
G4
ChiP-seq
Normalized eQTL density
Distance from G4 peak center

Figure S3, related to figure 1. G4 sequences identified from G4-seq and G4 ChIP-seq experiments are enriched for population variants.
a-c. Enrichment of mutations overlapping G4 sites derived from G4-seq and ChIP-seq experiments for **a.** K^+ treatment and **b.** PDS treatment, **c.** ChIP-seq experiment.
d-f Enrichment of mutations overlapping G4 sites correcting for trinucleotide context and mutation location, for G4-seq and ChIP-seq experiments for **d.** K^+ treatment and **e.** PDS treatment, **f.** G4 ChIP-seq experiment.
g. Venn diagram displaying the intersection between the two G4-seq experiments with PDS and K^+ treatments and the G4 ChIP-seq peaks.
h. Enrichment of variants from the center of G4 ChIP-seq peaks overlapping G4-seq peaks from PDS and K^+ treatments.
i. Enrichment of mutations overlapping G4 sites for SNPs, indels and structural variants at G4 peaks from G4 antibody treatment derived G4 sites.
j. eQTL density at the center of G4 ChIP-seq peaks overlapping G4-seq peaks from PDS and K^+ treatments.

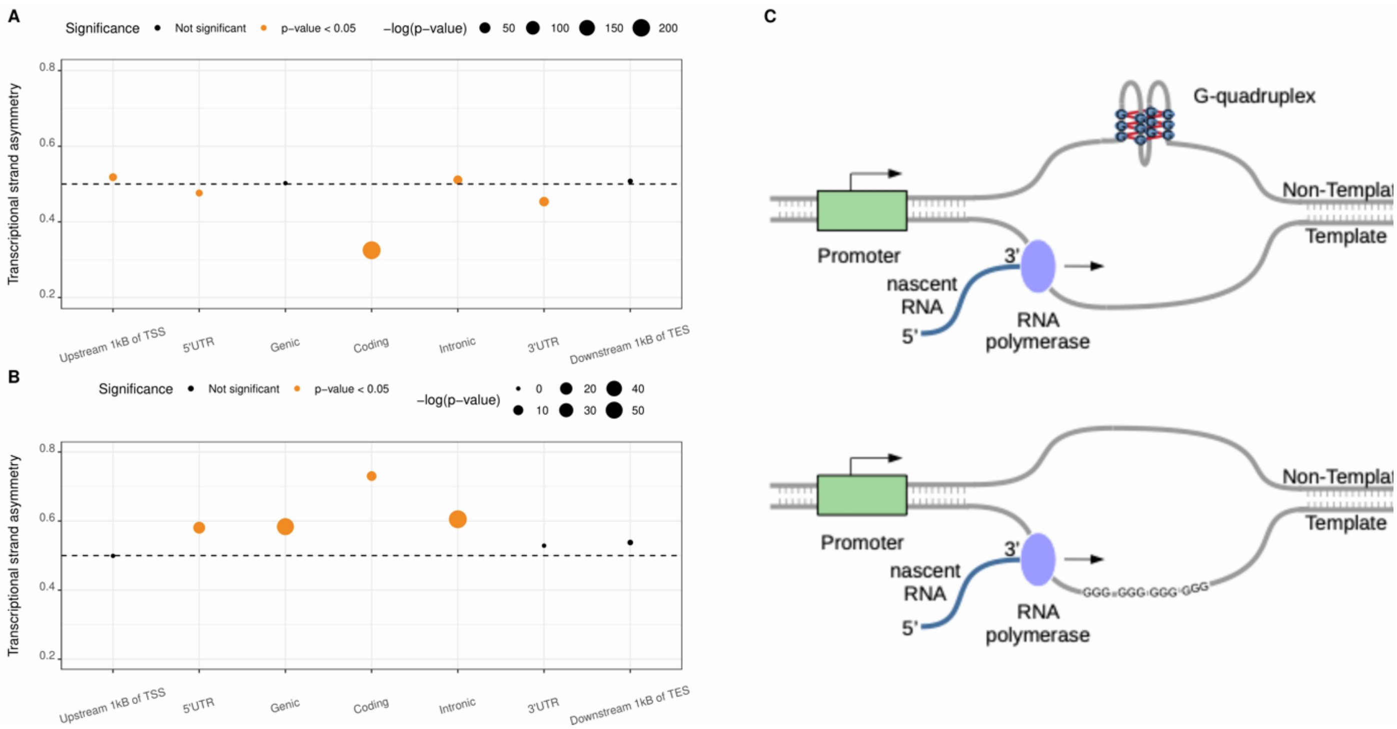

Figure S4, related to figure 1. G4 formation is influenced by its transcriptional orientation.
a. G4 motif strand bias between template and non-template strands.
b. Strand asymmetry of antibody-bound G4s across genic regions correcting for the background strand asymmetry of all the G4 motifs at each of the genic regions. Error bars represent standard deviation from bootstrapping. Statistical significance of strand asymmetry for antibody-bound G4s across genic regions correcting for the background strand asymmetry of all the G4 motifs at each of the genic regions. P-values are derived from binomial tests with Bonferroni correction.
c. G4s in vivo form preferentially at the non-template strand relative to the template strand, in which they could impede the RNA polymerase progression. Schematic representation of preferential G4 formation at the non-template strand (top) over the template strand (bottom) during transcription elongation.

A.

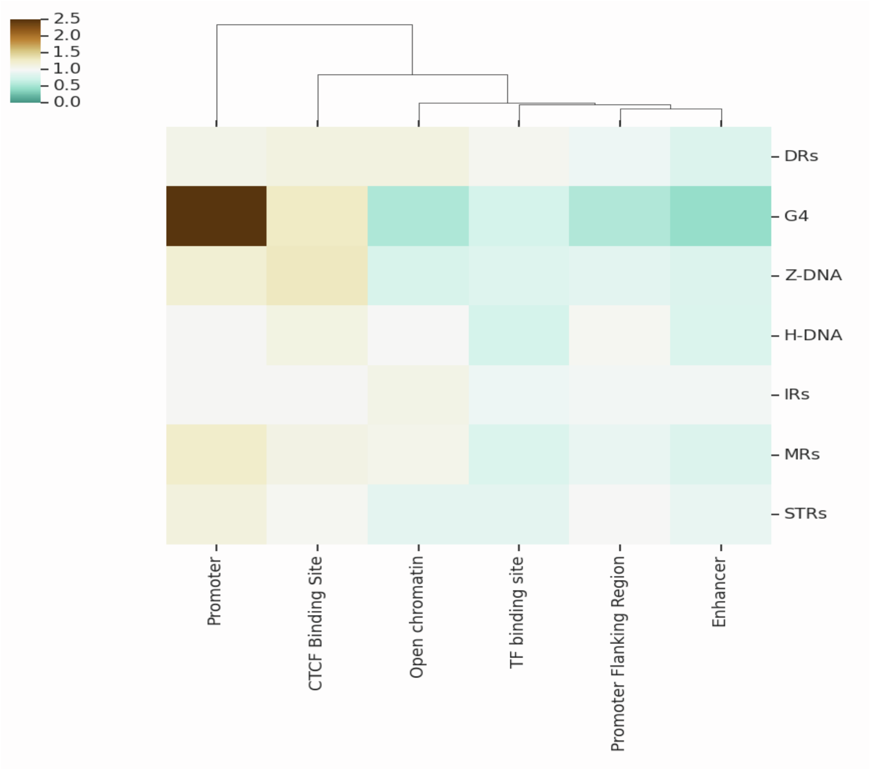
2.5
2.0
1.5
1.0
0.5
0.0
DRs
G4
Z-DNA
H-DNA
IRs
MRs
STRs
Promoter
CTCF Binding Site
Open chromatin
TF binding site
Promoter Flanking Region
Enhancer

B.

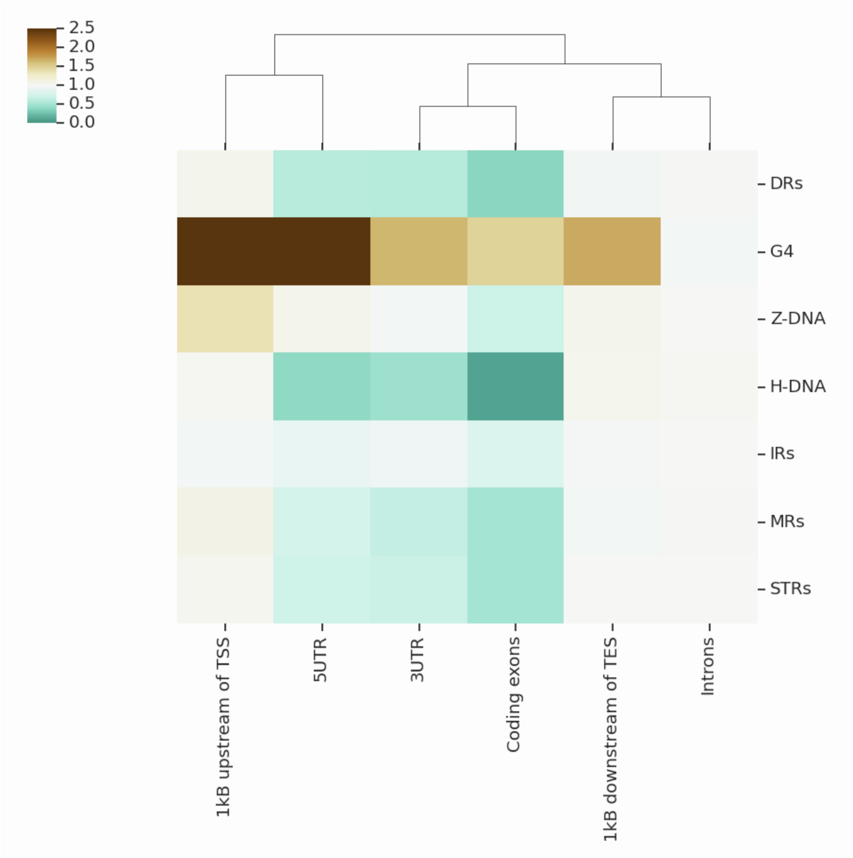
2.5
2.0
1.5
1.0
0.5
0.0
DRs
G4
Z-DNA
H-DNA
IRs
MRs
STRs
1kB upstream of TSS
5UTR
3UTR
Coding exons
1kB downstream of TES
Introns

Figure S5, related to figure 2. Non-B DNA motifs at regulatory regions and genes.
a. Enrichment of non-B DNA motifs across regulatory regions.
b. Enrichment of non-B DNA motifs across genic sub-compartments.

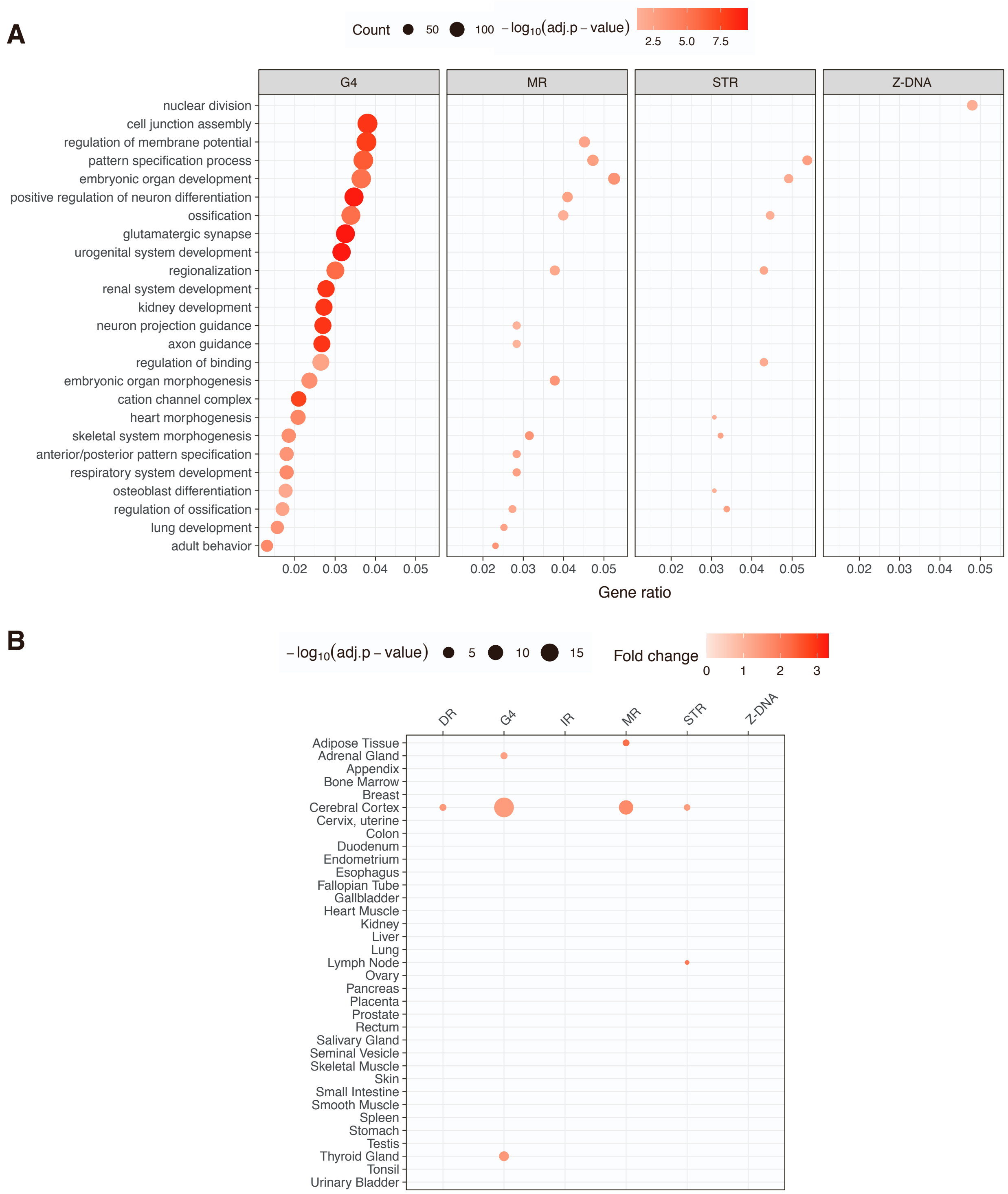

Figure S6, related to figure 2. Relationship between non-B DNA motifs at promoters, gene categories and tissue expression.

a. Gene ontology analysis of non-B DNA motifs found in gene promoters. For each non-B DNA motif up to the ten GO categories with highest gene ratio are shown.

b. Tissue-specific gene enrichment analysis for genes with non-B DNA motifs in their promoters.

A.

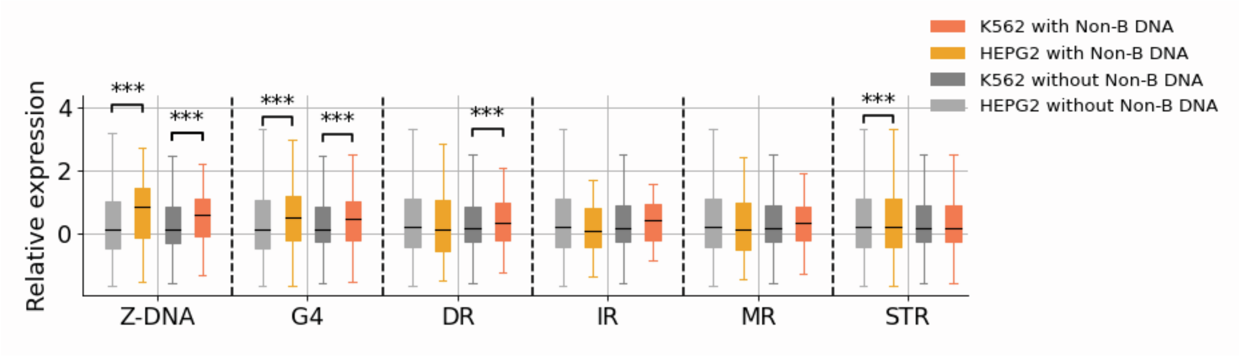

B.

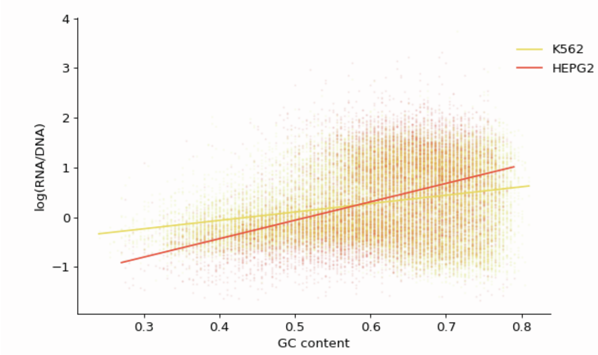

C.

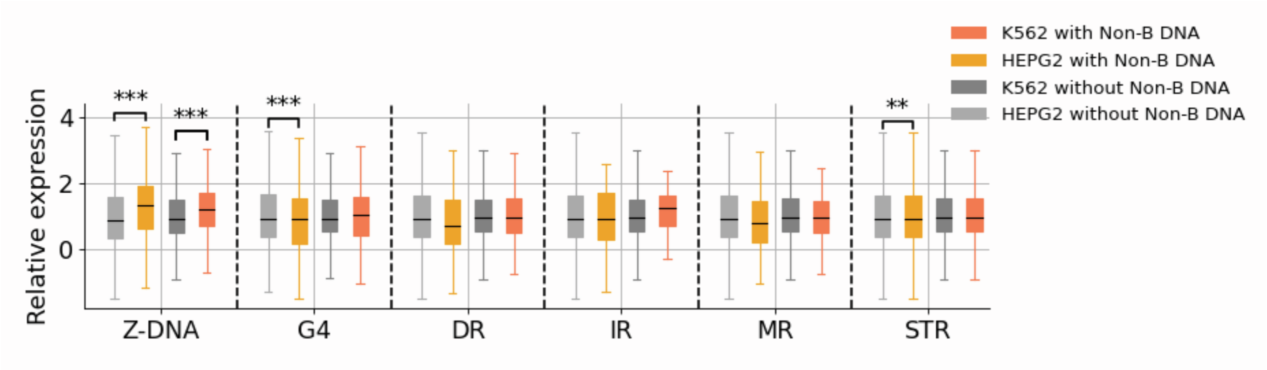

D.

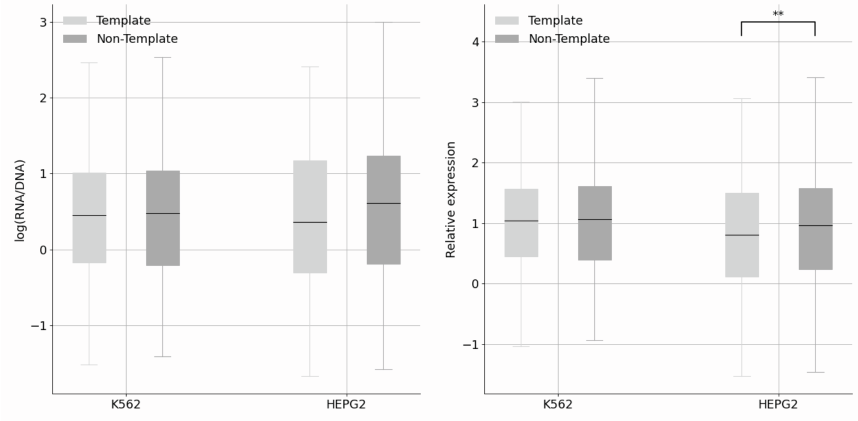

Figure S7, related to figure 3. Association between presence of different non-B DNA motifs and expression.

a. Expression of sequences with and without each non-B DNA motif are shown.
b. Fourth order polynomial model fitting the GC content and expression levels in HEPG2 and K562 cell lines.
c. Expression of sequences with and without each non-B DNA motif before and after GC content correction.
d. Expression of G4s found at the template and non-template orientations.
In figure panels adjusted p-values from t-tests with Bonferroni correction are displayed as * for p-value<0.05, ** for p-value<0.01 and *** for p-value<0.001.

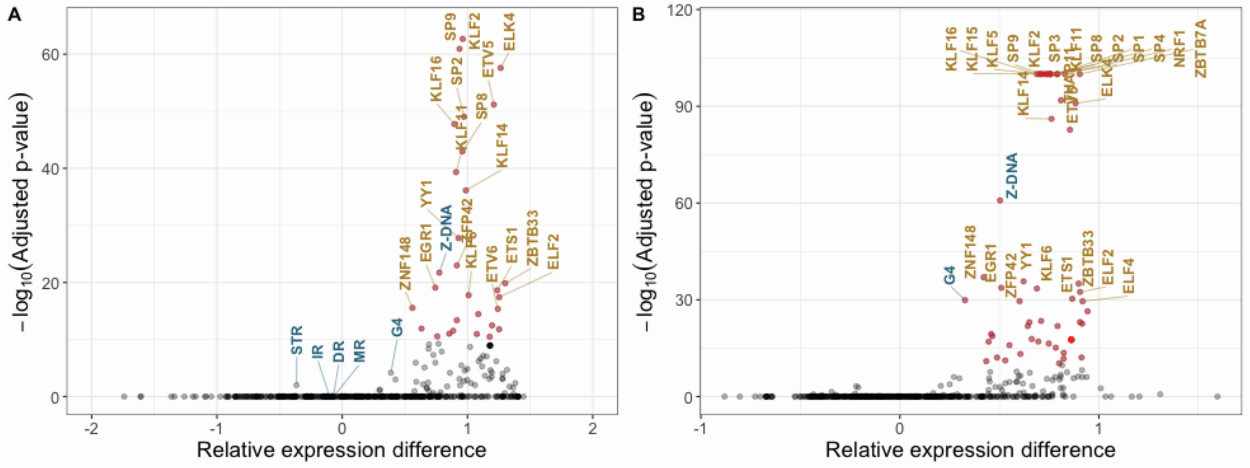

Figure S8, related to figure 3. Relative expression difference between the median expression for sequences with and without non-B DNA motifs and transcription factor binding sites. Results displayed in **A.** HepG2 and **B.** K562 cell lines, without performing GC content correction. Statistical significance is estimated with t-tests and Bonferroni correction.

A.

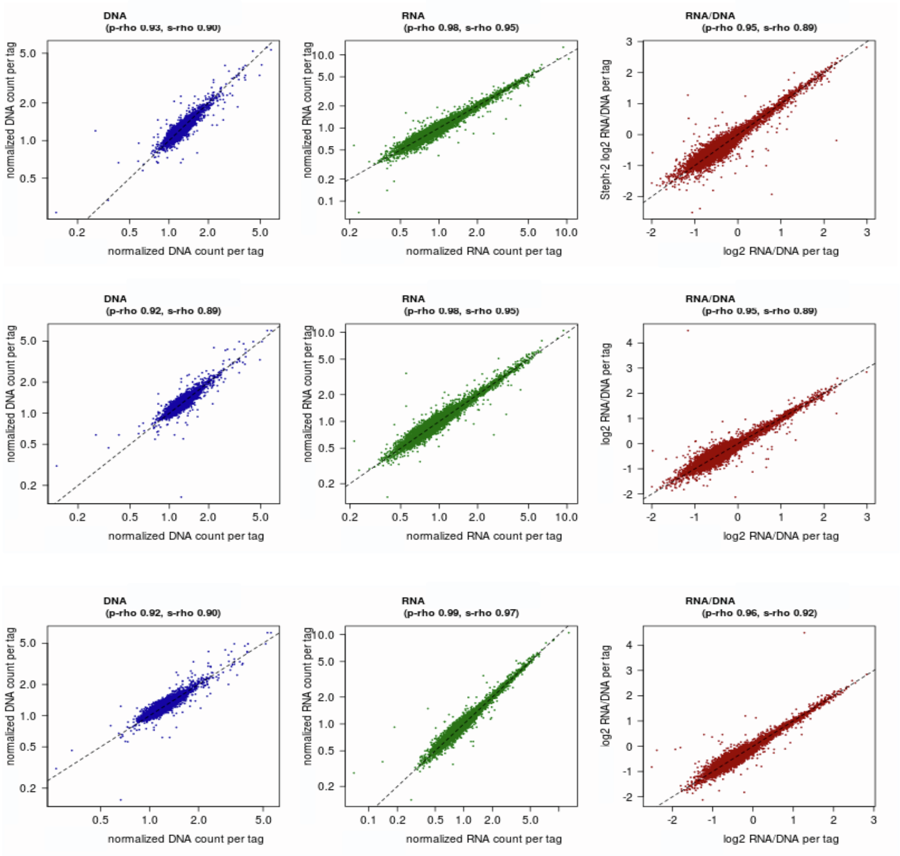
DNA
(p-rho 0.93, s-rho 0.90)
RNA
(p-rho 0.98, s-rho 0.95)
RNA/DNA
(p-rho 0.95, s-rho 0.89)
DNA
(p-rho 0.92, s-rho 0.89)
RNA
(p-rho 0.98, s-rho 0.95)
RNA/DNA
(p-rho 0.95, s-rho 0.89)
DNA
(p-rho 0.92, s-rho 0.90)
RNA
(p-rho 0.99, s-rho 0.97)
RNA/DNA
(p-rho 0.96, s-rho 0.92)
normalized DNA count per tag
normalized RNA count per tag
log2 RNA/DNA per tag
Steph-2 log2 RNA/DNA per tag

B.

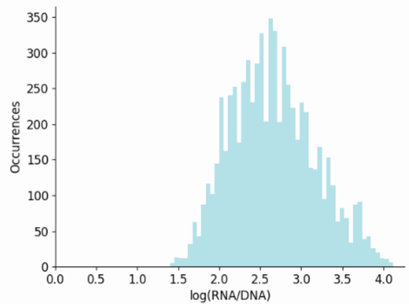
Occurrences
log(RNA/DNA)

C.

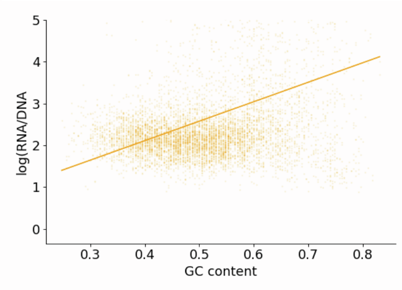
log(RNA/DNA)
GC content

D.

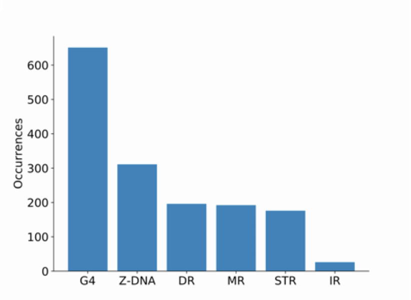
Occurrences
G4
Z-DNA
DR
MR
STR
IR

E.

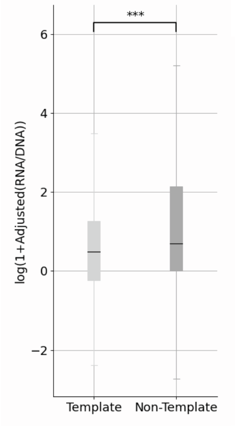
***
log(1+Adjusted(RNA/DNA))
Template
Non-Template

F.

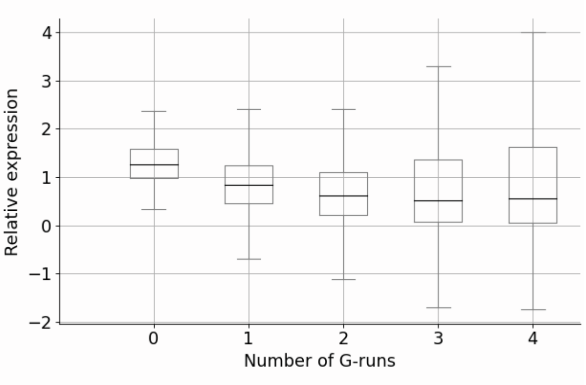
Relative expression
Number of G-runs

Figure S9, related to figure 4. Evaluation of non-B DNA motif regulatory roles using a neuronal MPRA.

a. Quality control between replicates of the MPRA experiment for the NPC cell line.

b. RNA/ DNA ratio for the autism dataset.

c. Scatter plot displaying association between expression levels and GC-content (Pearson correlation between GC content and expression levels: 0.32).

d. Number of occurrences of each non-B DNA motif category across the MPRA sequences

e. The orientation of G4s is significantly associated with expression (p-value<0.001, t-test).

f. Consecutive G-runs are associated with decreased expression levels.

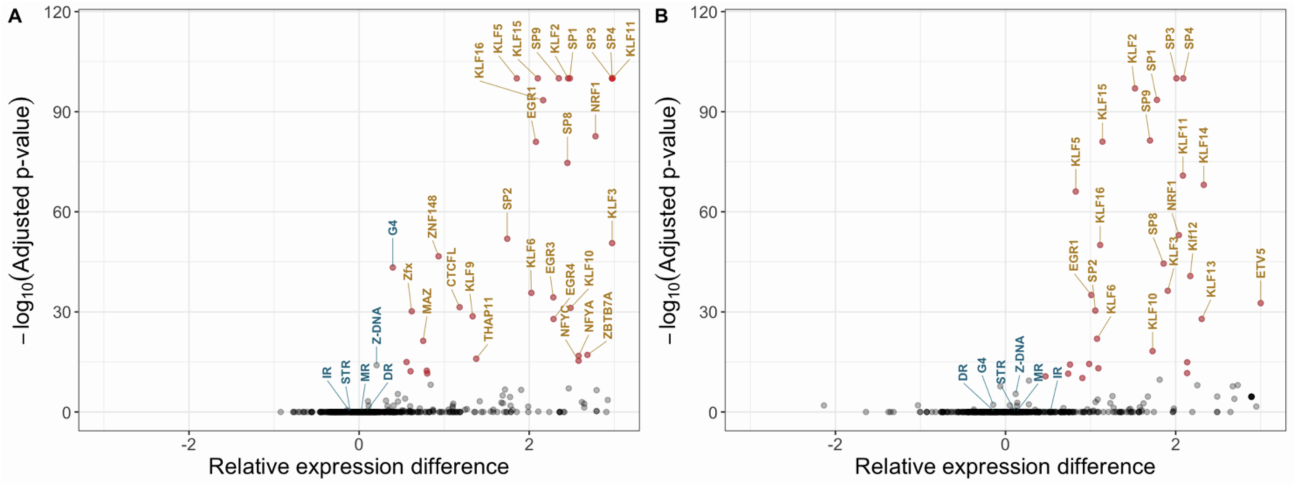

Figure S10, related to figure 4. Relative expression difference between the median expression for sequences with and without non-B DNA motifs and transcription factor binding sites in NPC cell lines.

a. Results displayed without performing GC content correction,

b. Results displayed after performing GC content correction.

Statistical significance is estimated with t-tests tests and Bonferroni correction.

A.

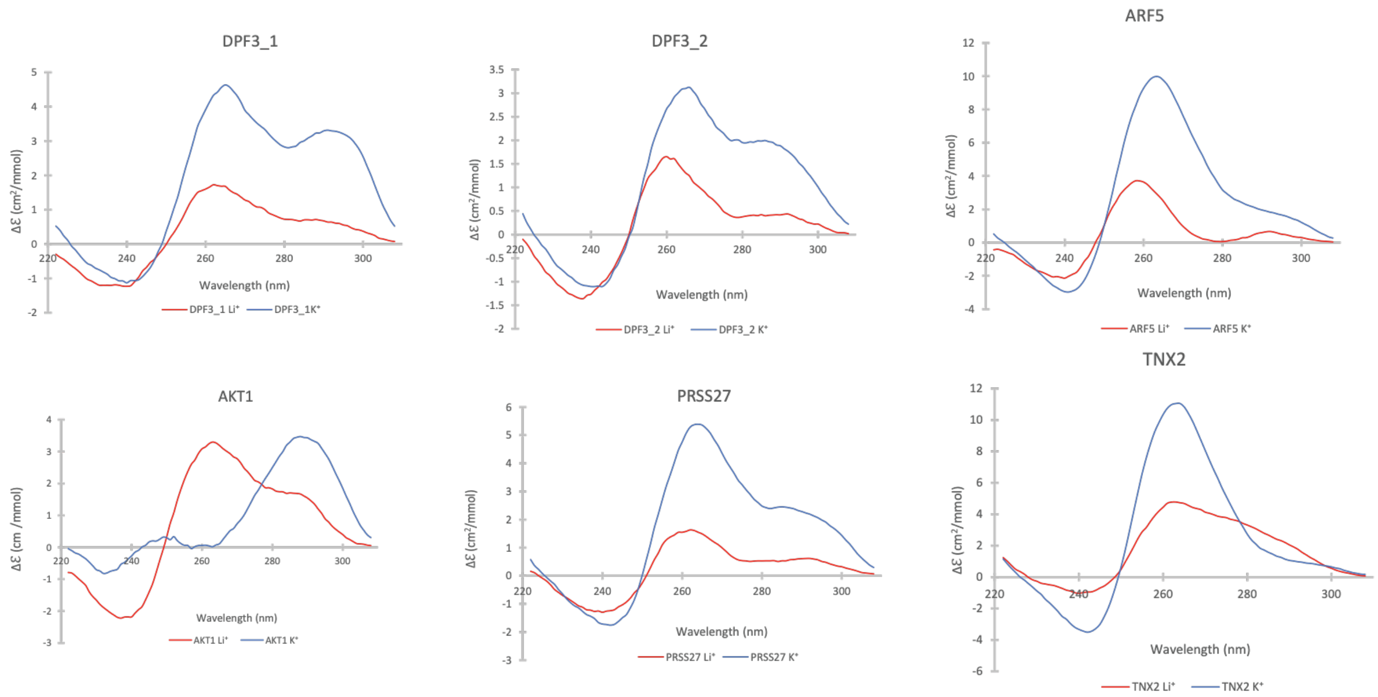
DPF3_1
DPF3_2
ARF5
AKT1
PRSS27
TNX2
Wavelength (nm)
Δε (cm²/mmol)
DPF3_1 Li⁺
DPF3_1K⁺
DPF3_2 Li⁺
DPF3_2 K⁺
ARF5 Li⁺
ARF5 K⁺
AKT1 Li⁺
AKT1 K⁺
PRSS27 Li⁺
PRSS27 K⁺
TNX2 Li⁺
TNX2 K⁺

B.

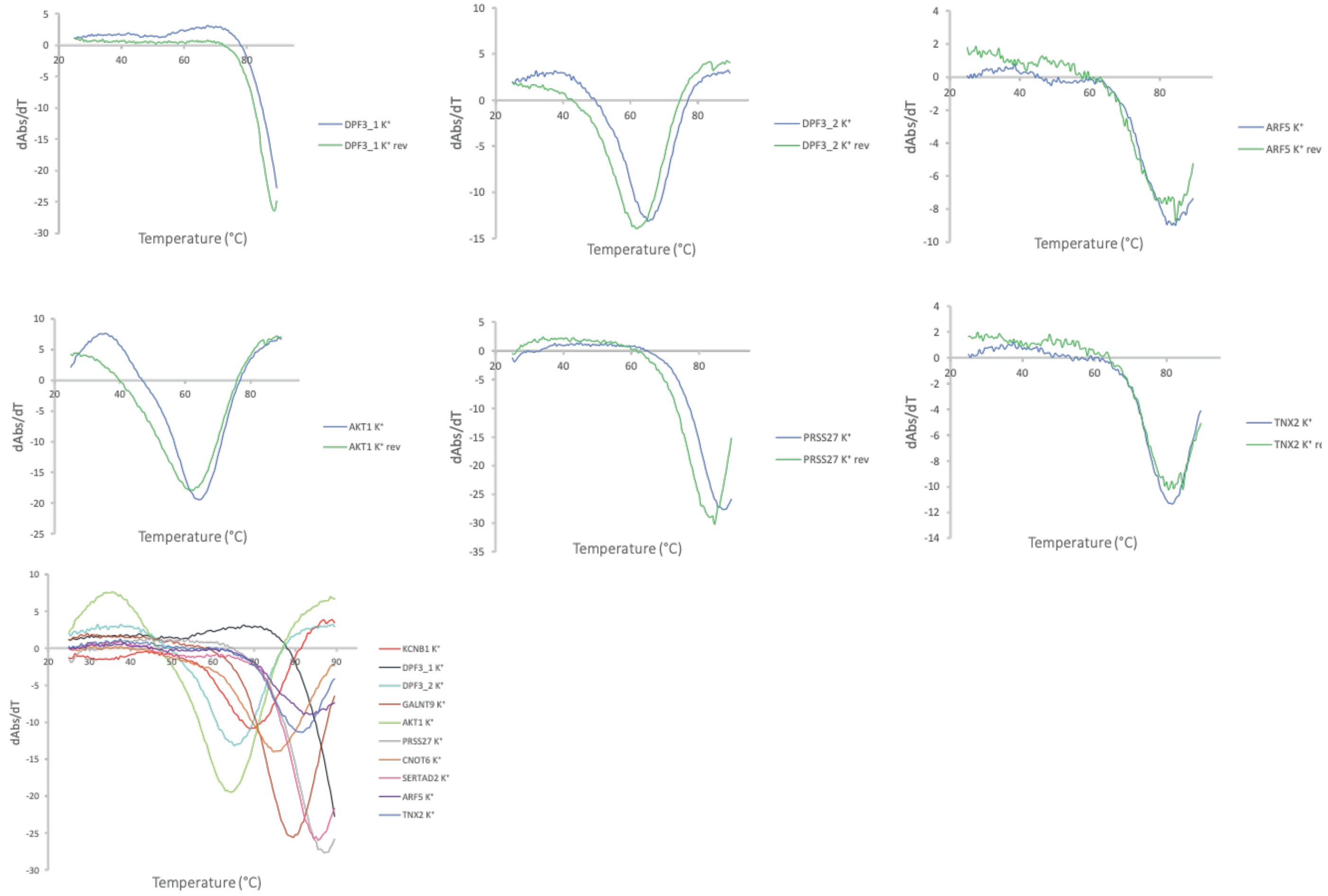
dAbs/dT
Temperature (°C)
DPF3_1 K⁺
DPF3_1 K⁺ rev
DPF3_2 K⁺
DPF3_2 K⁺ rev
ARF5 K⁺
ARF5 K⁺ rev
AKT1 K⁺
AKT1 K⁺ rev
PRSS27 K⁺
PRSS27 K⁺ rev
TNX2 K⁺
KCNB1 K⁺
GALNT9 K⁺
CNOT6 K⁺
SERTAD2 K⁺

Figure S11, related to figure 4. Experimental validation of G4 formation potential for selected sequences.

a. Circular dichroism (CD) spectra of the candidate targets for G4 formation potential in presence of two cations. The monovalent ion-dependent nature (G4 stabilized in K^+ but not in Li^+) indicate the formation of DNA G4s. b. UV melting profiles of the G4 candidates in presence of K^+.

b. The reverse melting profile (K^+rev) is also showed and matched well with the forward melting profile (K^+). Hyporchromic shift at 295nm is a hallmark for G4 formation, which can be transformed to negative peak in derivative plot (dAbs/dT) for G4 stability analysis. The melting temperature (Tm) of a G4 can be identified at the maximum negative value.

A.

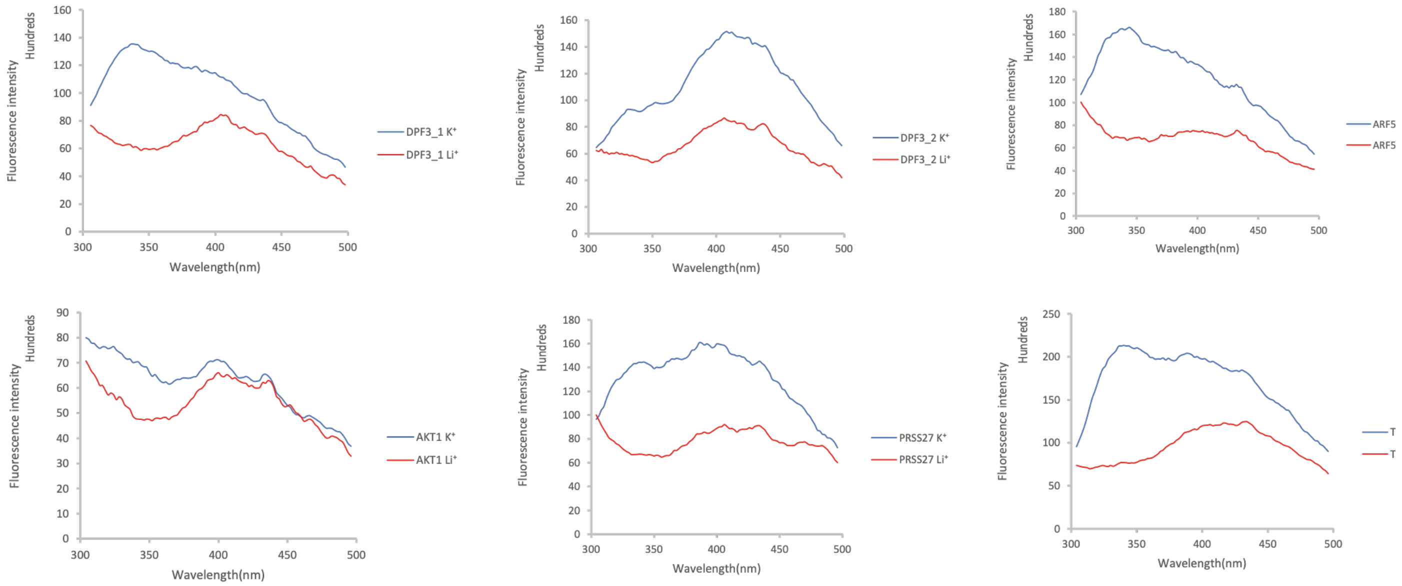

B.

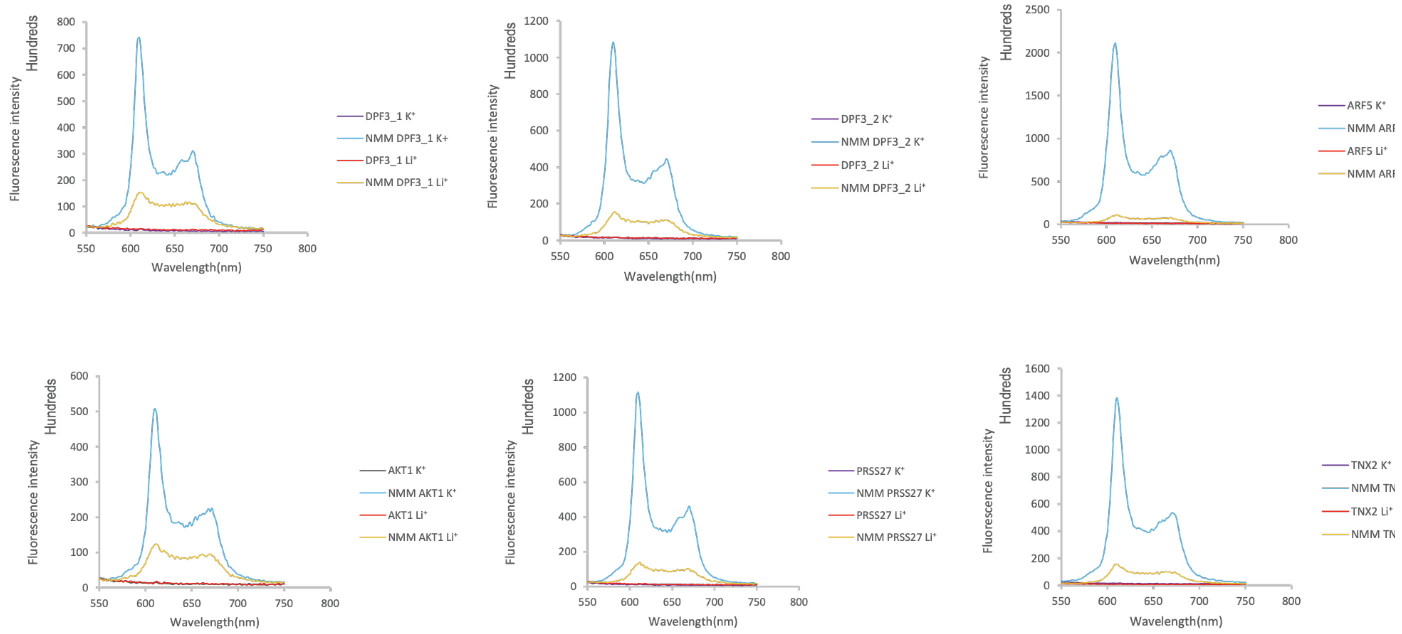

Figure S12, related to figure 4. Validation of G4 containing oligonucleotides using fluorescence based methods.

a. Intrinsic fluorescence of six candidate DNA oligonucleotides under Li^+ or K^+ conditions. The intrinsic fluorescence of G4s was increased when replacing Li^+ with K^+, highlighting the formation of DNA G4s.

b. Fluorescence emission associated with NMM ligand binding to G4 candidates in the presence of Li^+ or K^+ ions. In the absence of NMM ligand, no fluorescence was observed at ~610 nm. Upon NMM addition, weak fluorescence was observed under Li^+, which was substantially enhanced when substituted with K^+, supporting the formation of G4 which allows recognition of NMM and enhances its fluorescence.

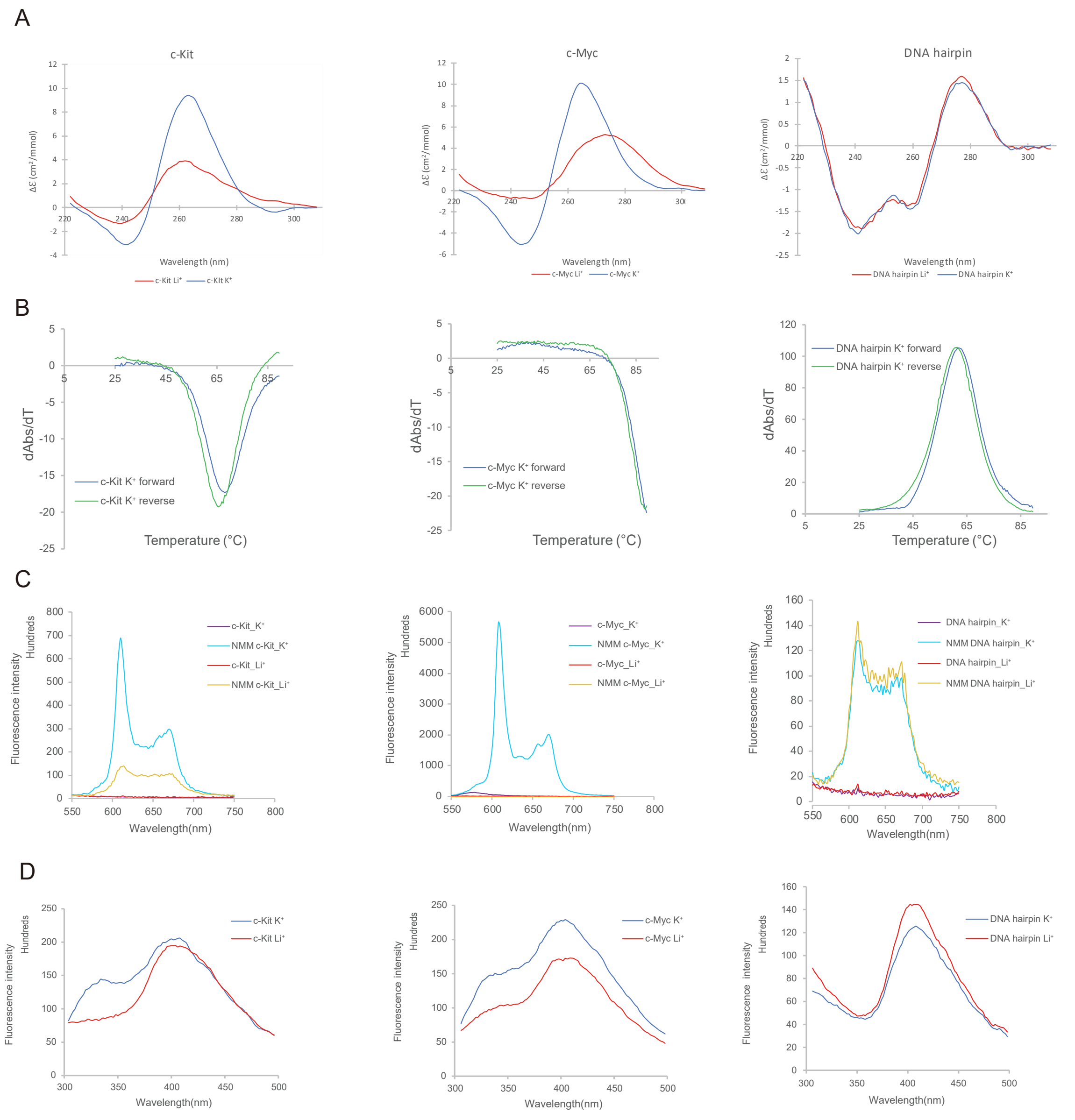

Figure S13, related to figure 4. Validation experiments performed with positive and negative control sequences.

a. Circular dichroism (CD) spectra of the candidate targets for G4 formation potential in presence of two cations. The monovalent ion-dependent nature (G4 stabilized in K^+ but not in Li^+) indicates the formation of DNA G4s, but not in DNA hairpin, the B-DNA motif.

b. UV melting profiles of the G4 candidates in presence of K^+. The reverse melting profile (K^+rev) is also shown and matched well with the forward melting profile (K^+). Hypochromic shift at 295nm is a hallmark for G4 formation, which can be transformed to a negative peak in the derivative plot (dAbs/dT) for G4 stability analysis. The melting temperature (Tm) of a G4 can be identified at the maximum negative value and the B-DNA motif showed a positive value at 260nm instead.

c. Fluorescence emission associated with NMM ligand binding to two G4-DNA and one B-DNA candidates in the presence of Li^+ or K^+ ions. In the absence of NMM ligand, no fluorescence was observed at ~610 nm. Upon NMM addition for two G4-DNAs, weak fluorescence was observed

under Li^+, which was substantially enhanced when substituted with K^+, supporting the formation of G4 which allows recognition of NMM and enhances its fluorescence. However, upon NMM addition for B-DNA, the fluorescence did not show a significant increase in the presence of Li^+ or K^+ ions which indicate no G4 formation for B-DNA with NMM.

d. Intrinsic fluorescence of two G4-DNA and one B-DNA candidates under Li^+ or K^+ conditions. The intrinsic fluorescence of G4s was increased when replacing Li^+ with K^+, highlighting the formation of DNA G4s but not in the B-DNA motif.

A.

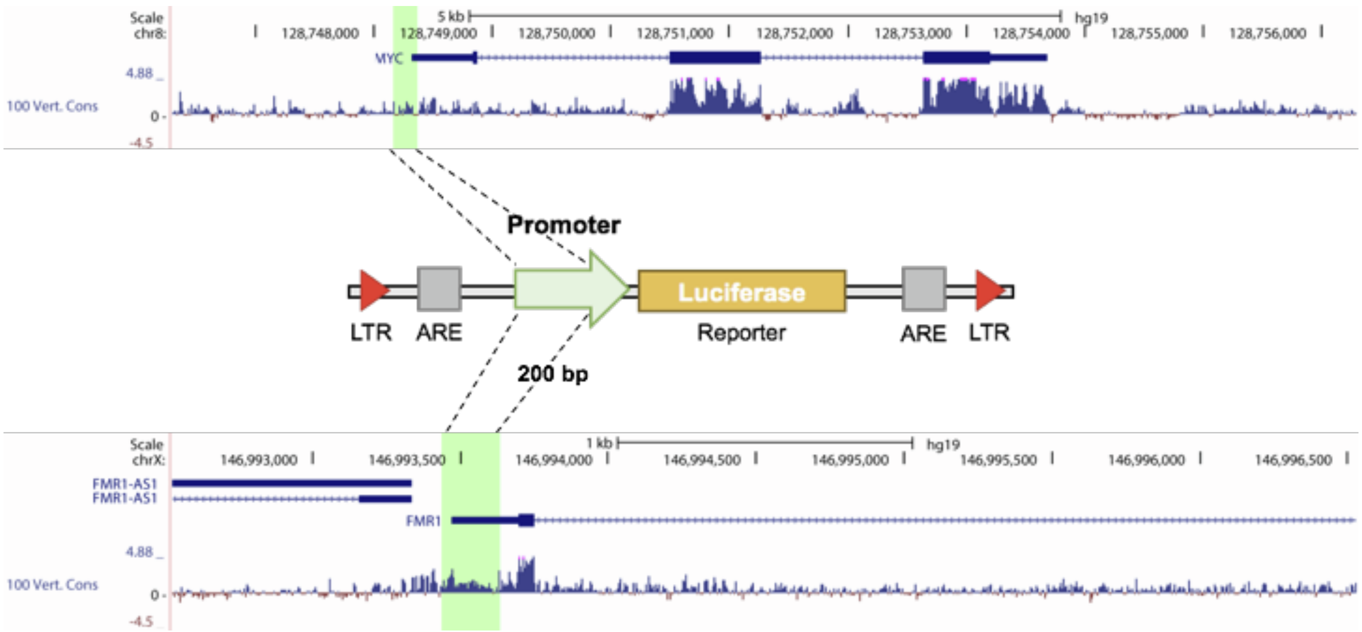

B.

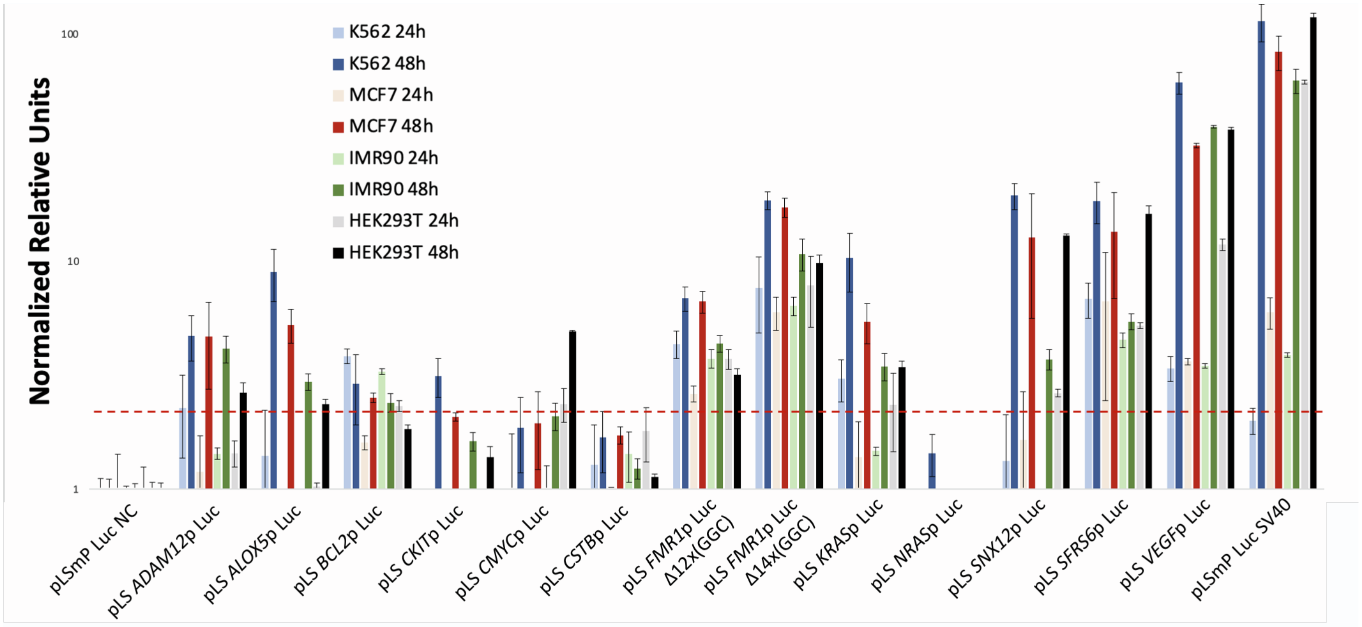

Figure S14, related to figure 5. Assessment of the activity of 12 disease-relevant gene promoters with non-B DNA motifs.

a. Schematic representation of the experimental design behind promoter activity assessment. In this example 200 bp near the TSS of the genes C-MYC and FMR1 are cloned right before a luciferase gene in a promoter-less lentiviral vector.

b. Luciferase assay in 4 different cell lines during 2 time points relative to a negative control (NC). Candidate promoters included two different constructs for the *FMR1* gene promoter, and a positive control (SV40). Results are normalized to the negative control. Red dashed line indicates the 2-fold threshold over the negative control.

A.

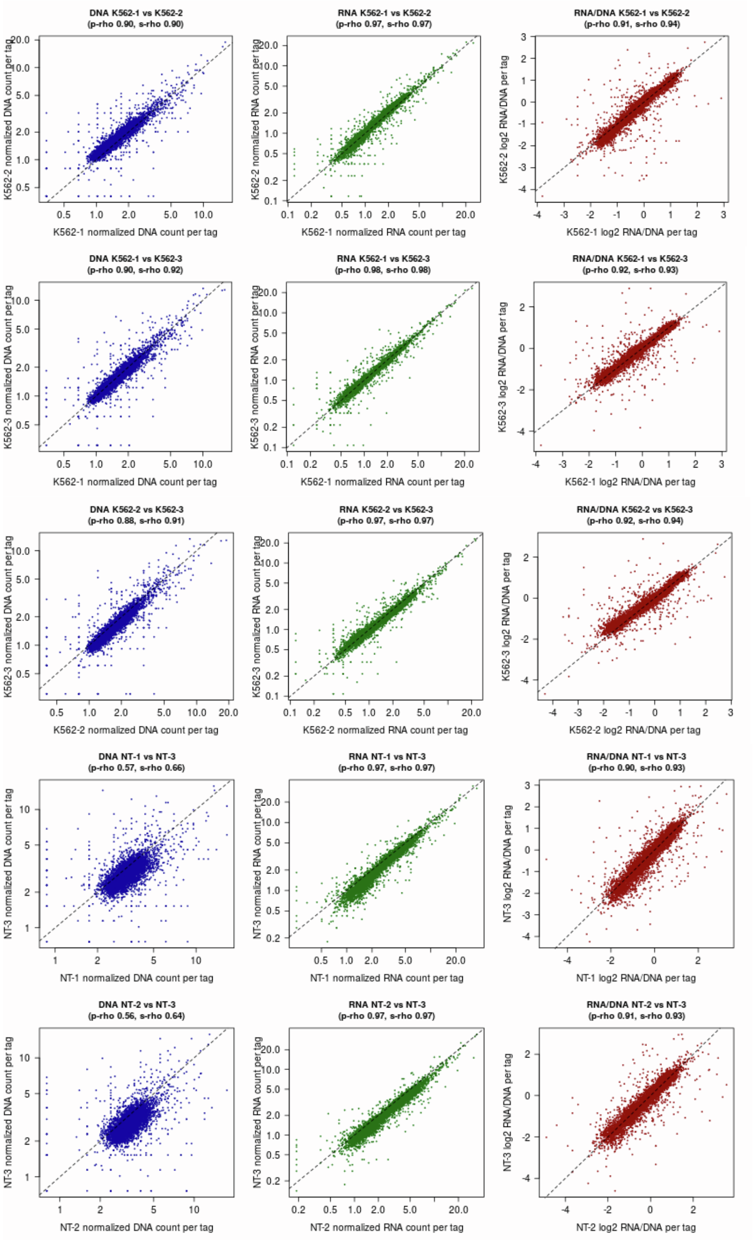

B.

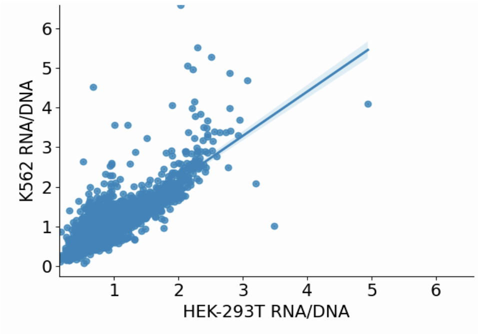

C.

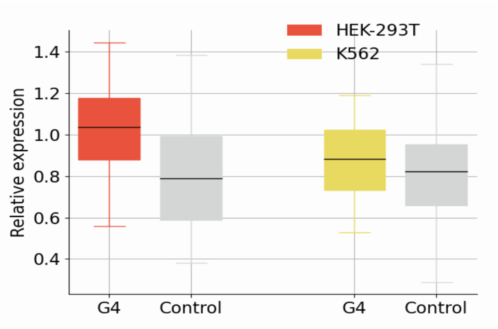

Figure S15, related to figure 5. MPRA quality control evaluation.

a. Quality control between replicates of the MPRA experiment for K562 and HEK-293T cell lines.

b. Comparison of expression levels of sequences between the two cell lines (Pearson r=0.87).
c. Mutations that disrupt G-quadruplexes relative to the original sequences in HEK-293T and K562 cell lines.
