# Supplementary material for: High-throughput characterization of the role of non-B DNA motifs on promoter function
Source: Cell Genom. 2022 Mar 15;2(4):100111. doi: 10.1016/j.xgen.2022.100111 (PMC9105345; doi:10.1016/j.xgen.2022.100111)
Supplement: Document S2. Transparent peer review — for Georgakopoulos-Soares et al. [file mmc2.pdf]

Cell Genomics

Transparent Peer Review Record

High-throughput characterization of the role of non-B DNA motifs on promoter function

Ilias Georgakopoulos-Soares, Jesus Victorino, Guillermo E. Parada, Vikram Agarwal, Jingjing Zhao, Hei Yuen Wong, Mubarak Ishaq Umar, Orry Elor, Allan Muhwezi, JoonYong An, Stephan J. Sanders, Chun Kit Kwok, Fumitaka Inoue, Martin Hemberg, Nadav Ahituv,

Summary

Initial submission:	Received : March 23rd 2021
	Scientific editor: Orli Bahcall, Judith Nicholson
First round of review:	Number of reviewers: 2<br>Revision invited : August 24th 2021<br>Revision received : October 21st 2021
Second round of review:	Number of reviewers: 2<br>Accepted :
Data freely available:	Yes
Code freely available:	Yes

This transparent peer review record is not systematically proofread, type-set, or edited. Special characters, formatting, and equations may fail to render properly. Standard procedural text within the editor's letters has been deleted for the sake of brevity, but all official correspondence specific to the manuscript has been preserved.

CellPress

Referees' reports, first round of review

Reviewer #1: CELL-GENOMICS-D-21-00105
In this manuscript the authors report the largest analysis conducted to date aimed at assessing the roles of sequences able to fold into nonB conformations on genomic structural variation and gene expression regulation. The field is of keen interest not only from the standpoint of evolutionary diversity within the human population, but also from the broader context of mechanisms of mutagenesis relevant to human disease and cancer. NonB DNA-forming sequences are abundant in the human genome and have consistently been observed to be associated with sites of genomic rearrangements and genetic diversification. The authors use a large dataset of single nucleotide polymorphism, they map the distribution of such motifs in different genomic regions, assess the expression of quantitative trait loci and, significantly, analyze their own massively parallel reporter assays (MPRA), both from published and ad hoc libraries.
The authors conclude that that deletions, insertions, and duplications are enriched in non-B DNA-forming motifs. Most non-B DNA-forming motifs are also enriched for SNPs, indels and structural variants at regulatory elements, and promoter regions display higher densities of non-B DNA-forming motifs than the rest of gene bodies. It was instructive for me to note that although sequences with G4 and Z-DNA motifs show increased expression levels, after correcting for GC content G4s are no longer associated with increased expression. These data on G4 are significant.
Despite the large amount of data used and massive of work conducted, the study raises some questions regarding the search criteria used and the lack of availability of the codes on GitHub. These questions need to be addressed to convey sufficient confidence on all conclusions reached by the authors.

Specific points
1. The authors used the dataset of nonB DNA-forming motifs from the nonB DB database described by Cer et al. (2013). The dataset reports all MRs, and MRs other that those that can form H-DNA are not known to form specific nonB DNA structures. If the authors did not filter the data for the subset of MRs that form H-DNA, i.e. the motifs that contain all purines or all pyrimidines on the same strand, then the data for MRs are not related to the formation of H-DNA structures. If

they did perform the filtering, it should be noted.
2. Both IRs and MRs from the dataset above include loop sizes up to 100 bases. Large loop sizes are not easily conducive to intramolecular structures, and therefore these motifs should be filtered for loop size, keeping preferably those below 10 bases or so. The python codes from the authors did keep loop size at or below 4 bases, and it would have been informative to compare the results from the Cer dataset with those from the MPRA searches with similar loop sizes. Again, if the authors filtered the data, they should note it as this is an important point.
3. I was not able to find the python scripts used by the authors for the lentiMPRA data on the GitHub site reported. There, I was expecting to see a brief description of the scripts in the README.md file, a status bar showing the types of computing languages used, and the scripts on the main page or a folder. By inspecting the codes I would have been interested in whether the MRs did conform to the H-DNA type of structures, as elaborated above, if Z-DNA contained any ApT or TpA steps, i.e. purine-pyrimidine steps that are unlikely to support Z-DNA, and what types of files did the scripts accept as input. In the absence of such information, it is difficult to evaluate the validity of the conclusions.

Other points
4. Supplementary Figure 1a and page 7. "We observed an excess of SNPs directly overlapping non-B DNA motifs". It may be helpful to the reader to caution that the difference is very small and that the highly significant p-value originates from the extremely large sample size.
5. Supplementary Figure 1g and page 7. "For example, STRs had a higher frequency of deletions"; in my opinion this is a consequential finding, considering the mechanisms known to lead to deletions at tandem repeats (including slippage and primer realignment). It may be informative to briefly mention this point in Discussion.
6. Figure 1. "Adjusted p-values displayed as…". Sorry I did not understand which two categories were used for pairwise comparisons (control dataset versus experimental dataset?).
7. Page 41 and elsewhere. It was not clear how "controlling for trinucleotide content" was performed.
8. Page 11. "reflecting a preference in structure formation at promoters in vivo". Reference to Figure 2g is missing.
9. Figure 2. "IRs, MRs, DRs, STRs and G4s are abbreviations for inverted repeats,

mirror repeats, direct repeats, short tandem repeats and G-quadruplexes respectively." Would this seem more appropriate in Figure 1?

10. Page 15. "The association between G-runs and gene expression was further investigated, finding that consecutive G-runs result in decreased expression when accounting for their GC content contribution ". Did the effect worsen with G4 tracts longer than 4 GGG runs?
11. Page 16. "fluorescent-based arrays N-methyl mesoporphyrin IX (NMM) ligand enhanced fluorescence and intrinsic fluorescence experiments". Please check the grammar.
12. Page 16. "Combined, these results validate that these sequences have G4 structures". Maybe change to "form G4 structures in vitro".
13. Figure 4b and c. Panels did not match the legends.
14. Figure 4f. I did not see the minima being above 85 C in all cases. The close correspondence between the forward and reverse curves is nice.
15. Page 19. "For G4s, we introduced a single, two or three mutations in one, two, three or every G-run at the original G4 genomic sites and found that sequences with the disruptions in the G- runs did not display significant expression differences from the original sites when aggregated (Supplementary Figure 13c)." This sentence was not clear, and the figure did not help understanding. "We found that there was a statistically significant reduction in expression following the disruption of Z- DNA motifs (Figure 5d), supporting the notion that they are activating sequences." This was confusing to me, what does control mean? Figures 5f and 5g should be 5e and 5f.
16. Figure 5. I was wondering if the scheme in panel a should not be shown earlier with the first lentiMPRA data. "The oligonucleotide library is PCR amplified and barcoded at the 5′ UTR using a degenerate reverse primer. Cloning of PCR products into a promoter-less lentiviral vector". Please check the grammar. Panel d, I found this to be confusing, what do control and Z-DNA mean?
17. Page 21. "We also observe an excess of eQTLs in the vicinity of non-B DNA motifs and at experimentally identified G4s the eQTL enrichment was even larger than for G4 motifs alone". Do you mean larger than with the aggregate G4 motifs?
18. Page 21. "These results are suggestive of inhibitory effects of G4s, which can be mis- characterized due to the effect of their nucleotide composition." The intended meaning is not clear. What are mis-characterized, the G4 or gene inhibition?
19. Page 21. "Thus, targeting these sequences could pose as a potential

therapeutic", please check the grammar.
20. Supplementary Table 2. Plus and minus strands are ambiguous; maybe template and non-template.
21. Supplementary Figure 1. Maybe specify that the standard error is smaller than the resolution of the image.
22. Supplementary Figure 2. Were z-scores relative to control?
23. Supplementary Figure 3. I did not understand the first part of the legend.
24. Supplementary Figure 4 and elsewhere. Fonts are very small, can they be increased? I would also suggest "Schematic representation of preferential G4 formation at the non-template strand (top) over the template strand (bottom) during transcription elongation."
25. Supplementary Figure 6. Panel b, isn't this a linear regression? In panel c, is before and after or just after?
26. Supplementary Figure 9. Are these negative log10 values?
27. Supplementary Figure 12. Do you mean 12 disease-relevant gene promoters? Please check that official gene symbols are in italic. Panel b, not clear, at 2 time points relative to a negative control?
28. Supplementary Figure 13. Panel c, is was not clear to me where were the original G4 sequences and where were the mutations. The plot is the same as for Z-DNA but the conclusions are opposite. I did not understand.
29. Page 41. Please use the "micro" symbol rather than the letter "u" and, consistently, capital L for liter.
30. Page 43. There is a space missing on line 3. LiCac - lithium cacodylate? "the molar residue ellipticity were obtained and then", please correct the tense.
31. Page 44. "lower the chance of vaporization of the sample", is evaporation more appropriate? "The collected data were deducted by the blanked solutions which have the identical", which had.
32. Data availability. I was not able to locate the PRJNA* repositories when searching the web. Could you please be more specific?

Reviewer #2: In the manuscript titled "High-throughput characterization of the role of non-B DNA motifs on promoter function" from Nadav Ahituv's lab, Georgakopoulos-Soares et al performed numerous analyses to deepen our knowledge of non B-DNA motifs and their role in gene regulation. They start with an examination of the association between these motifs and genetic variation,

showing that non B-DNA motifs are enriched for several types of variation. They further explore the correlation between non B-DNA motifs and expression, looking for enrichments of eQTLs and promoter sequences. Finally, they look at previous MPRA data and data generated for this study to verify enrichment for promoter activity in non B-DNA motifs.
In my opinion, the authors convincingly showed that non B-DNA motifs, and likely non B-DNA itself (Z-DNA and G4), have a role in regulation of gene expression, contributing to our understanding of how DNA structure may influence gene expression. The authors presented a multitude of analyses, which may have made the presentation more complicated than it could have been. Whereas it is good to have several lines of evidence supporting an analysis, it becomes tiring to go through similar results and various supplemental figures.

Overall, the manuscript is adequately written, but I would personally prefer a more streamlined paper. I will leave this choice to the authors' and editor's discretion.

Minor revisions:

1. Add abbreviations (IR, DR, etc) to Figures 1A-G.

2. The difference in Figure S1a seems to be very small, what is the fold-difference? How many observations were used? The statistical significance could be due to the large number of observations.

3. Text in several Figures and Supplemental figures is too small (e.g. Figure S1A-F, Figure 2A-G). Most figures will need to be redone to show text in adequate size. The authors could take the opportunity and label panels. There are many similar analyses resulting in similar panels, so figure titles could help speed up figure interpretation.

4. In Figure S1B there seems to be periodical enrichment of H-DNA and STR. What is the author's interpretation? Is the periodicity due to artifacts?

5. Figure 2e doesn't show what the authors claim in the text. It does not compare TSS with the region upstream. This panel is unnecessary, the next panel (2f) shows the enrichment across the TSS.

6. Regarding Figure 3a-b, what is the Gene Ontology of genes that have promoters with non-B DNA motifs? Are they housekeeping genes?

7. Figure 4 The authors should provide reference spectra for known G4-DNA and B-DNA. Whereas a clear difference between the stabilizing and non-stabilizing salt conditions is shown, it's not possible to judge whether the stabilizing condition leads to actual G4-DNA conformation without a reference spectrum, particularly to a genomic audience not used to judging DNA spectra.

5. In Figure 4F, CNOT6 and SERTAD2 do not show differences between conditions. Can the authors comment on this lack of effect?

6. Figure 5d shows control and Z-DNA, not disruption like the text and legend indicate. The title of the figure says, "Mutated Z-DNA". Does it refer to "disrupted Z-DNA"? Expression of Z-DNA is higher than the control, is "control" actually the disrupted Z-DNA? Which gene is the sequence from? Only one promoter was tested?

7. Figure 5 naming is incorrect in the text. 5e was skipped.

8. Unit is missing in figure 5F (bp?).

9. Check legend of Figure 5, should be Bonferroni correction and there's a missing comma after panels in the last sentence.

10. The authors should attempt to provide some commentary on the reasons behind the correlations and enrichment between the various non B-DNA motifs and the features analyzed. Can they speculate on mechanisms behind the different enrichments?

Authors’ response to the first round of review

Reviewer #1: In this manuscript the authors report the largest analysis conducted to date aimed at assessing the roles of sequences able to fold into nonB conformations on genomic structural variation and gene expression regulation. The field is of keen interest not only from the standpoint of evolutionary diversity within the human population, but also from the broader context of mechanisms of mutagenesis relevant to human disease and cancer. NonB DNA-forming sequences are abundant in the human

genome and have consistently been observed to be associated with sites of genomic rearrangements and genetic diversification. The authors use a large dataset of single nucleotide polymorphism, they map the distribution of such motifs in different genomic regions, assess the expression of quantitative trait loci and, significantly, analyze their own massively parallel reporter assays (MPRA), both from published and ad hoc libraries. The authors conclude that that deletions, insertions, and duplications are enriched in non-B DNAforming motifs. Most non-B DNA-forming motifs are also enriched for SNPs, indels and structural variants at regulatory elements, and promoter regions display higher densities of non-B DNA-forming motifs than the rest of gene bodies. It was instructive for me to note that although sequences with G4 and Z-DNA motifs show increased expression levels, after correcting for GC content G4s are no longer associated with increased expression. These data on G4 are significant. Despite the large amount of data used and massive of work conducted, the study raises some questions regarding the search criteria used and the lack of availability of the codes on GitHub. These questions need to be addressed to convey sufficient confidence on all conclusions reached by the authors.

We thank the reviewer for their constructive inputs and overall positive assessment. We have now considerably expanded on our methodology both in the manuscript and in GitHub, including the code and materials and methods section. We have also performed additional analyses to respond to the specific issues raised below. Below we provide a point by point response.

Specific points 1. The authors used the dataset of nonB DNA-forming motifs from the nonB DB database described by Cer et al. (2013). The dataset reports all MRs, and MRs other that those that can form H-DNA are not known to form specific nonB DNA structures. If the authors did not filter the data for the subset of MRs that form H-DNA, i.e. the motifs that contain all purines or all pyrimidines on the same strand, then the data for MRs are not related to the formation of H-DNA structures. If they did perform the filtering, it should be noted.

We thank the reviewer for their comment and apologize for this omission in our methods section. We have indeed performed the filtering. In accordance with the previous definition of Cer et al. 2013 and our previous work (Georgakopoulos-Soares et al. 2018), we use the same definition of H-DNA motifs. We have now added in our methods section the following statement to clarify this:

“The subset of MRs that have high AG content (>90%) and which are more likely to form H-DNA structures. Here, H-DNA motifs were defined as the subset of MRs that have a high (>90%) AG content, arm lengths of >=10bp and spacer size of less than 8bp.”

2. Both IRs and MRs from the dataset above include loop sizes up to 100 bases. Large loop sizes are not easily conducive to intramolecular structures, and therefore these motifs should be filtered for loop size, keeping preferably those below 10 bases or so. The python codes from the authors did keep loop size at or below 4 bases, and it would have been informative to compare the results from the Cer dataset with those from the MPRA searches with similar loop sizes. Again, if the authors filtered the data, they should note it as this is an important point.

We thank the reviewer for their comment. In our previous work (Georgakopoulos-Soares et al. 2018), we showed the association between loop and arm length and their effect on mutability across cancer genomes. IRs, MRs and DRs with large loops represent a very small fraction of the motifs examined. However, we have now performed an analysis of the mutagenesis of IRs, MRs and DRs as a function of the loop size as well as the arm size which we provide below. We find that indeed smaller loop/spacer length is associated with higher relative mutational density and larger arm lengths are associated with increased mutability. Similar to the rest of the manuscript mutational enrichment was calculated relative to the simulated controls, with values above 1 representing higher mutation rate than expected by chance. For the presented analyses arm length was considered between 10-25bp and spacer length

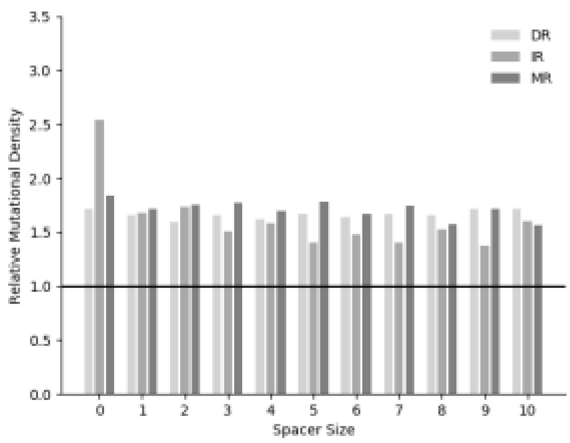

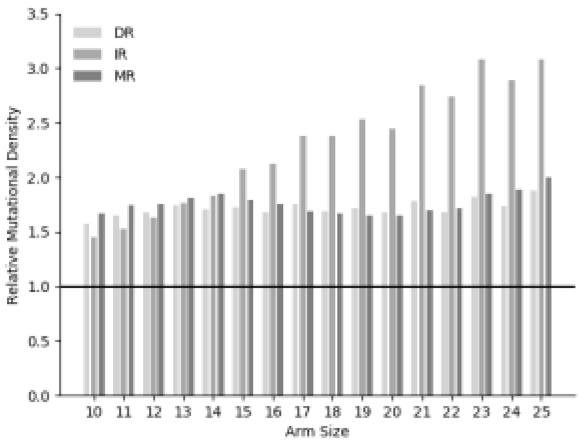

3. I was not able to find the python scripts used by the authors for the lentiMPRA data on the GitHub site reported. There, I was expecting to see a brief description of the scripts in the README.md file, a status bar showing the types of computing languages used, and the scripts on the main page or a folder. By inspecting the codes I would have been interested in whether the MRs did conform to the H-DNA type of structures, as elaborated above, if Z-DNA contained any ApT or TpA steps, i.e. purine-pyrimidine steps that are unlikely to support Z-DNA, and what types of files did the scripts accept as input. In the absence of such information, it is difficult to evaluate the validity of the conclusions.

We apologize for this shortcoming from our end. We have now added a folder called MPRA_scripts in which we present the functions used for the Non-B DNA motif detection. Indeed, Z-DNA containing ApT or TpA steps were not considered. H-DNA motifs were only considered for the mutation analysis described earlier due to the low number of sequences as described in point 1 and the revised methods section. We also provide a README.txt file explaining the functions and corrections used. We have also added additional scripts used in the other parts of the manuscript. The status bar shows mostly HTML code because the file Promoter_figures.nb.html contains all the R code used throughout the manuscript and the visualizations generated and can be opened for inspection as an Html page. This is the largest file and has the most lines of code and therefore the bar shows that the code is mostly HTML.

Other points

4. Supplementary Figure 1a and page 7. "We observed an excess of SNPs directly overlapping non-B DNA motifs". It may be helpful to the reader to caution that the difference is very small and that the highly significant p-value originates from the extremely large sample size.

We thank the reviewer for their comment and we agree with this conclusion. Indeed, the larger differences are observed for particular non-B DNA motifs. We have now modified this statement to reflect this: “We observed an excess of SNPs directly overlapping non-B DNA motifs (Supplementary Figure 1a, MannWhitney U, p-value <0.0001), but the magnitude of the effect was small and the highly significant p-value was due to the large sample size.”

5. Supplementary Figure 1g and page 7. "For example, STRs had a higher frequency of deletions"; in my opinion this is a consequential finding, considering the mechanisms known to lead to deletions at tandem repeats (including slippage and primer realignment). It may be informative to briefly mention this point in Discussion.

We thank the reviewer for their comment. We have now added the following statement in the discussion: “Different mechanisms underlying the higher mutation rate at individual non-B DNA motifs have been previously identified, such as DNA polymerase slippage errors at microsatellites causing deletions (Bacolla et al., 2004), which was also observed in this study.”

6. Figure 1. "Adjusted p-values displayed as…". Sorry I did not understand which two categories were used for pairwise comparisons (control dataset versus experimental dataset?).

Apologies. This statement refers to the Fisher Exact test we performed, which is now rewritten as: “Adjusted p-values from Fisher’s exact tests are displayed as * for p-value <0.05, ** for p-value <0.01 and

*** for p-value <0.001."

We have added the following statement in the methods section: "Fisher's exact tests with Bonferroni correction were performed across regulatory elements (Figure 1i), comparing the number of occurrences of each non-B DNA motif at each regulatory element and across all regulatory elements."

7. Page 41 and elsewhere. It was not clear how "controlling for trinucleotide content" was performed.

We have modified our methods section to better explain how we have generated the controls, provided below: "To achieve this, the base-pair at the randomly selected simulated position, within 10kb from the original mutation, and both the 5' and the 3' adjacent base-pairs had to match those at the mutated sites, and the mutation and simulation sites had to be different from one another."

8. Page 11. "reflecting a preference in structure formation at promoters in vivo". Reference to Figure 2g is missing.

We thank the reviewer; we have now added this figure reference in the text.

9. Figure 2. "IRs, MRs, DRs, STRs and G4s are abbreviations for inverted repeats, mirror repeats, direct repeats, short tandem repeats and G-quadruplexes respectively." Would this seem more appropriate in Figure 1?

We agree with the reviewer and we have now added the same abbreviation in Figure 1h, where the terms are introduced. We have added the following statement: "DR, G4, IR, MR and STR refer to direct repeats, G-quadruplexes, inverted repeats, mirror repeats and short tandem repeats, respectively."

10. Page 15. "The association between G-runs and gene expression was further investigated, finding that consecutive G-runs result in decreased expression when accounting for their GC content contribution ". Did the effect worsen with G4 tracts longer than 4 GGG runs?

We thank the reviewer for this comment. The error bars become quite large and the uncertainty increases as we increase the number of G-runs due to the small number of sequences with this property. We provide the schematic below.

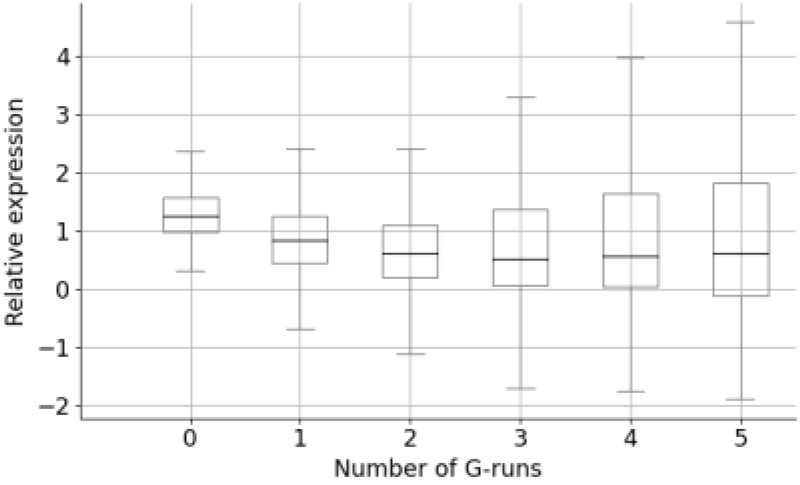

11. Page 16. "fluorescent-based arrays N-methyl mesoporphyrin IX (NMM) ligand enhanced fluorescence and intrinsic fluorescence experiments". Please check the grammar.

We have corrected this to: "To confirm the results from the circular dichroism and UV-melting experiments, we used fluorescentbased arrays including N-methyl mesoporphyrin IX (NMM) ligand enhanced fluorescence and intrinsic fluorescence experiments"

12. Page 16. "Combined, these results validate that these sequences have G4 structures". Maybe change to "form G4 structures in vitro".

We thank the reviewer. We have now added this clarification which is also provided below: "Combined, these results validate that these sequences form G4 structures in vitro."

13. Figure 4b and c. Panels did not match the legends.

We thank the reviewer for pointing this out. We have now corrected the legends.

14. Figure 4f. I did not see the minima being above 85 C in all cases. The close correspondence between the forward and reverse curves is nice.

Thank you for the comment. That was a typo and we have now removed this phrase.

15. Page 19. "For G4s, we introduced a single, two or three mutations in one, two, three or every G-run at the original G4 genomic sites and found that sequences with the disruptions in the G- runs did not display significant expression differences from the original sites when aggregated (Supplementary Figure13c)." This sentence was not clear, and the figure did not help understanding. "We found that there was a statistically significant reduction in expression following the disruption of Z- DNA motifs (Figure 5d), supporting the notion that they are activating sequences." This was confusing to me, what does control mean? Figures 5f and 5g should be 5e and 5f.

We thank the reviewer for this comment. We have revised this sentence, in which we were trying to say that the G4s that had one or more mutations at the G-runs were compared to the original sequences (without any mutations) and we could not see significant differences in expression. The modified sentence now reads: “For G4s, we introduced a single, two or three mutations in one, two, three or every G-run at the original G4 genomic sites. We compared the mutated sequences to the original sequence and found that sequences with the disruptions in the G-runs did not display significant expression differences from the original sequences (Supplementary Figure 15c).” For sequences with Z-DNA motifs, we designed sequences with scrambled versions of the original Z-DNA sequence or the modification of purines to pyrimidines as disruptions, both of which served as controls. We have now modified the sentence for more clarity to: “We designed MPRA sequences with scrambled Z-DNA motifs or with disruptions of purines to pyrimidines in the alternating purine-pyrimidine tract, which served as disrupted Z-DNA controls. We found that there was a statistically significant reduction in expression following the disruption of Z-DNA motifs (Figure 5d), supporting the notion that they are activating sequences.” We have now corrected Figures 5f and 5g to be 5e and 5f.

16. Figure 5. I was wondering if the scheme in panel a should not be shown earlier with the first lentiMPRA data. "The oligonucleotide library is PCR amplified and barcoded at the 5' UTR using a degenerate reverse primer. Cloning of PCR products into a promoter-less lentiviral vector". Please check the grammar. Panel d, I found this to be confusing, what do control and Z-DNA mean?

As the schematic is tailored to the MPRA experimental design we performed to directly test the effect of non-B DNA motifs, we would prefer to keep it in this order. The previous two MPRA experiments were based on promoter sequences found in the genome, that either have or do not have non-B DNA motifs. Here, in the third experiment, the promoter templates were modified to directly test hypotheses driven by Non-B DNA motifs. To better clarify this, we modified this sentence to read: “The oligonucleotide library is PCR amplified and barcoded at the 5' UTR using a degenerate reverse primer. Cloning of PCR products into a lentiviral promoter assay vector was performed next.” For panel d:

For sequences with Z-DNA motifs, we designed sequences with scrambled versions of the original Z-DNA sequence or the modification of purines to pyrimidines as disruptions, both of which served as controls. We have now modified the paragraph that explains this fiding for more clarity to: “We designed MPRA sequences with scrambled Z-DNA motifs or with disruptions of purines to pyrimidines in the alternating purine-pyrimidine tract, which served as disrupted Z-DNA controls. We found that there was a statistically significant reduction in expression following the disruption of Z-DNA motifs (Figure 5d), supporting the notion that they are activating sequences.”

17. Page 21. "We also observe an excess of eQTLs in the vicinity of non-B DNA motifs and at experimentally identified G4s the eQTL enrichment was even larger than for G4 motifs alone". Do you mean larger than with the aggregate G4 motifs?

We thank the reviewer for this comment. Yes, we refer to the enrichment antibody-bound sites relative to aggregate G4 motifs. We have rewritten these sentences to clarify this, as provided below: “We also observe an excess of eQTLs in the vicinity of non-B DNA motifs. In particular, at experimentally identified G4s the eQTL enrichment was even larger than that observed across G4 motifs (Figure 1j-k), which is likely due to the formation of G4 motifs being more frequent in open chromatin regions, nucleosome-depleted regions (Hänsel-Hertsch et al., 2016).”

18. Page 21. "These results are suggestive of inhibitory effects of G4s, which can be mis- characterized due to the effect of their nucleotide composition." The intended meaning is not clear. What are mischaracterized, the G4 or gene inhibition?

This referred to the need to take into account the GC content in modelling the role of G4s in promoter regulation. We have revised this sentence since it was indeed not clear as written below: "These results are suggestive of inhibitory effects of G4s in promoters, which can be mis-characterized if the effect of GC content is not taken into consideration as well as orientation-dependent regulatory effects."

19. Page 21. "Thus, targeting these sequences could pose as a potential therapeutic", please check the grammar.

We thank the reviewer. We have now revised and provide the new sentence below: "Thus, targeting these sequences in key regulatory sites could be a potential novel therapeutic path (Balasubramanian et al., 2011)"

20. Supplementary Table 2. Plus and minus strands are ambiguous; maybe template and non-template

We thank the reviewer for the correction. We have now added the terms Reference and Complement instead and have added the following statement in the table legend: "The human genome reference strand is defined as "Reference" and reference reverse complement strand as "Complement."

21. Supplementary Figure 1. Maybe specify that the standard error is smaller than the resolution of the image.

We thank the reviewer, we have now added this to the figure legend.

22. Supplementary Figure 2. Were z-scores relative to control?

The relative scores were calculated relative to the mean. We have now added the following statement in the Methods section to better reflect this: "Across regulatory elements, z-scores were calculated from the density of mutations at non-B DNA motifs at that element, relative to the mean mutational density at that element, divided by the standard deviation (Supplementary Figure 2)."

23. Supplementary Figure 3. I did not understand the first part of the legend.

We apologize for that. We have now corrected this figure legend, which we provide below: "Supplementary Figure 3: a-c. G4 sequences identified from G4-seq and G4 ChIP-seq experiments are enriched for population variants. a-c. Enrichment of mutations overlapping G4 sites derived from G4-seq and ChIP-seq experiments for a. K+ treatment and b. PDS treatment, c. ChIP-seq experiment. a-c Enrichment of mutations overlapping G4 sites correcting for trinucleotide context and mutation location, for G4-seq and ChIP-seq experiments for d. K+ treatment and e. PDS treatment, f. G4 ChIP-seq experiment. g. Venn diagram displaying the intersection between the two G4-seq experiments with PDS and K+ treatments and the G4 ChIP-seq peaks. h. Enrichment of variants from the center of G4 ChIP-seq peaks overlapping G4-seq peaks from PDS and K+ treatments. i. Enrichment of mutations overlapping G4 sites for SNPs, indels and structural variants at G4 peaks from G4 antibody treatment derived G4 sites. k. eQTL density at the center of G4 ChIP-seq peaks overlapping G4-seq peaks from PDS and K+ treatments."

24. Supplementary Figure 4 and elsewhere. Fonts are very small, can they be increased? I would also suggest "Schematic representation of preferential G4 formation at the non-template strand (top) over the template strand (bottom) during transcription elongation."

We have now added the suggestion in the figure legend and we have replotted the figure panels with larger fonts. We have also increased the font sizes in the figure panels in other parts of the paper

25. Supplementary Figure 6. Panel b, isn't this a linear regression? In panel c, is before and after or just after?

For Panel b, it is a linear regression. For panel c is after, panel a is before GC correction.

26. Supplementary Figure 9. Are these negative log10 values?

We apologize for that, these are indeed negative and we have corrected this.

27. Supplementary Figure 12. Do you mean 12 disease-relevant gene promoters? Please check that official gene symbols are in italic. Panel b, not clear, at 2 time points relative to a negative control?

We thank the reviewer. We have now corrected it to: "Assessment of the activity of 12 disease-relevant gene promoters with non-B DNA motifs." We have fixed the figure legend for panel b to: "Luciferase

assay in 4 different cell lines during 2 time points relative to a negative control (NC). Candidate promoters included two different constructs for the FMR1 gene promoter, and a positive control (SV40).” We have updated the figure panel to have the gene symbols in italics, which we also provide below.

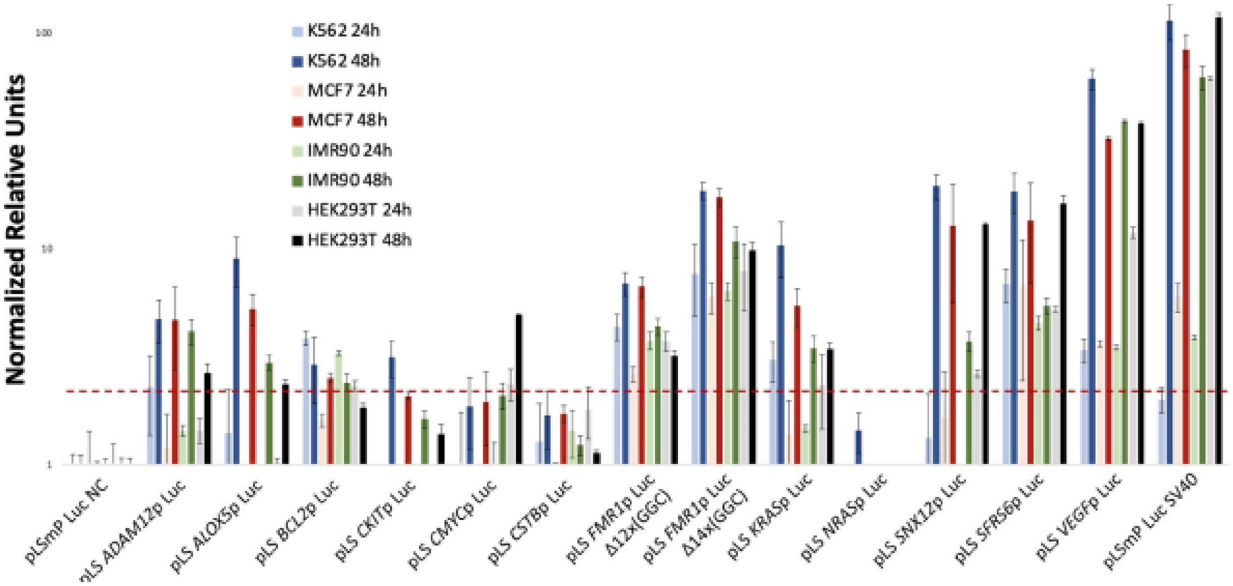

28. Supplementary Figure 13. Panel c, is was not clear to me where were the original G4 sequences and where were the mutations. The plot is the same as for Z-DNA but the conclusions are opposite. I did not understand.

We thank the reviewer for the comment. There are multiple mutations that have been introduced at different parts of the G4 sequences to disrupt their G-runs. Since the MPRA consisted of 7,500 sequences, we were able to generate many types of mutations, all of which introduced disruptions of the non-B DNA motif. We have revised this sentence and the modified sentence is now: “For G4s, we introduced a single, two or three mutations in one, two, three or every G-run at the original G4 genomic sites We compared the mutated to the original sequences and found that sequences with the disruptions in the G-runs did not display significant expression differences from the original sites (Supplementary Figure 15c).” For sequences with Z-DNA motifs, we introduced two types of disruptions. We designed sequences with scrambled versions of the original Z-DNA sequence or the modification of purines to pyrimidines as disruptions, both of which were controls in which the Z-DNA motif is disrupted. We have modified the sentence to: “We designed MPRA sequences with scrambled Z-DNA motifs or with disruptions of purines to pyrimidines in the alternating purine-pyrimidine tract, which served as disrupted Z-DNA controls. We found that there was a statistically significant reduction in expression following the disruption of Z-DNA motifs (Figure 5d), supporting the notion that they are activating sequences.”

29. Page 41. Please use the "micro" symbol rather than the letter "u" and, consistently, capital L for liter.
We thank the reviewer, we have now added µL for consistency across the manuscript.

30. Page 43. There is a space missing on line 3. LiCac - lithium cacodylate? "the molar residue ellipticity were obtained and then", please correct the tense.

We have now added the abbreviation of lithium cacodylate referring to LiCac. We have corrected the tense.

31. Page 44. "lower the chance of vaporization of the sample", is evaporation more appropriate? "The collected data were deducted by the blanked solutions which have the identical", which had.

We have corrected vaporization to evaporation. We have corrected the sentence to: “The collected data were deducted by the blanked solutions which had the identical concentrations of the KCl and LiCac buffer (pH 7.0) only.”

32. Data availability. I was not able to locate the PRJNA* repositories when searching the web. Could you please be more specific?

We thank the reviewer. You can find the data publicly at:

https://www.ncbi.nlm.nih.gov/biosample?Db=biosample&DbFrom=bioproject&Cmd=Link&LinkName=bioproject_biosample&LinkReadableName=BioSample&ordinalpos=1&IdsFromResult=763774 Under the project at: https://www.ncbi.nlm.nih.gov/bioproject/763774

Reviewer #2:

In the manuscript titled "High-throughput characterization of the role of non-B DNA motifs on promoter function" from Nadav Ahituv's lab, Georgakopoulos-Soares et al performed numerous analyses to deepen our knowledge of non B-DNA motifs and their role in gene regulation. They start with an examination of the association between these motifs and genetic variation, showing that non B-DNA motifs are enriched for several types of variation. They further explore the correlation between non BDNA motifs and expression, looking for enrichments of eQTLs and promoter sequences. Finally, they look at previous MPRA data and data generated for this study to verify enrichment for promoter activity in non B-DNA motifs. In my opinion, the authors convincingly showed that non B-DNA motifs, and likely non B-DNA itself (ZDNA and G4), have a role in regulation of gene expression, contributing to our understanding of how DNA structure may influence gene expression. The authors presented a multitude of analyses, which may have made the presentation more complicated than it could have been. Whereas it is good to have several lines of evidence supporting an analysis, it becomes tiring to go through similar results and various supplemental figures. Overall, the manuscript is adequately written, but I would personally prefer a more streamlined paper. I will leave this choice to the authors' and editor's discretion.

We thank the reviewer for their overall positive assessment. We provide below a point by point response to the points raised. We have also performed the additional experiments and analyses requested to provide additional evidence for our insights.

Minor revisions: 1. Add abbreviations (IR, DR, etc) to Figures 1A-G.

We apologize for this omission. We have now added the following sentence in Figure legend 1h, where the abbreviations are introduced: “DR, G4, IR, MR and STR refer to direct repeats, G-quadruplexes, inverted repeats, mirror repeats and short tandem repeats respectively

2. The difference in Figure S1a seems to be very small, what is the fold-difference? How many observations were used?

The statistical significance could be due to the large number of observations. Indeed, the statistical significance is due to the very large numbers (Enrichment of 1.03-fold), with more than 10 million non-B DNA motifs (Cer et al. 2013). However, the observed mutation patterns were largely dependent on individual non-B DNA motif categories, which was further dissected in Figures S1b-c. We have added the following statement to reflect that the observed statistically significant difference is driven by the large numbers and the effect has a very small magnitude: “We observed an excess of SNPs directly overlapping non-B DNA motifs (Supplementary Figure 1a, MannWhitney U, p-value <0.0001), but the magnitude of the effect was small and the highly significant p-value was due to the large sample size.”

3. Text in several Figures and Supplemental figures is too small (e.g. Figure S1A-F, Figure 2A-G). Most figures will need to be redone to show text in adequate size. The authors could take the opportunity and label panels. There are many similar analyses resulting in similar panels, so figure titles could help speed up figure interpretation.

We thank the reviewer. We have resized Figure 2A-G, Supplementary Figure 1, Supplementary Figure 2, Supplementary Figure 4, Supplementary Figure 5, Supplementary Figure 7, Supplementary Figure 9, Supplementary Figure 10, Supplementary Figure 11, Supplementary Figure 12, Supplementary Figure 14 and Supplementary Figure 15.

4. In Figure S1B there seems to be periodical enrichment of H-DNA and STR. What is the author's interpretation? Is the periodicity due to artifacts?

We thank the reviewer for their comment. We have encountered such periodicities in our previous manuscript as well (Georgakopoulos-Soares et al. 2018) and it has been shown previously that non-B DNA motifs influence the positioning of nucleosomes (Kouzine et al. 2017). Due to the distance from the center (~150bp), it most likely reflects nucleosome positioning patterns for specific non-B DNA motifs.

5. Figure 2e doesn't show what the authors claim in the text. It does not compare TSS with the region upstream. This panel is unnecessary, the next panel (2f) shows the enrichment across the TSS.

We have now corrected the explanation for this figure panel. Figure panel 2e shows the relative

enrichment of the non-B DNA motif density at the (-250bp,0) to that of (-1kB,0) for each motif, therefore indicating that closer to the TSS, the frequency of non-B DNA motifs is higher for most. Figure panel 2f shows only the region immediate to the TSS. We have updated the figure legend which we provide below: “e. Enrichment of non-B DNA motifs in the [-250, 0] region relative to the wider promoter region (-1kB,0). Error bars represent standard deviation from bootstrapping.”

6. Regarding Figure 3a-b, what is the Gene Ontology of genes that have promoters with non-B DNA motifs? Are they housekeeping genes?

Great suggestion! To test this, we performed a GO term analysis in promoter upstream regions. For G4s, Z-DNA motifs and MRs we found multiple terms associated with the majority being developmental and in particular neuronal related, while we could not find terms associated with IRs and DRs. We also performed a tissue enrichment analysis finding genes associated with the cerebral cortex as the most enriched tissue for the majority of non-B DNA motifs. We have added this in Supplementary Figure 6 and we have also added the following text: “We also performed a GO term analysis in promoter upstream regions. For G4s, Z-DNA motifs and MRs we found multiple terms associated with developmental processes, such as Pattern specification process (GO:0007389), Embryonic organ development (GO:0048568) and Positive regulation of neuron differentiation (GO:0045666), (Supplementary Figure 6a). As these analyses suggest that some non-B DNA motifs could control tissue-specific gene expression, we used TissueEnrich to calculate enrichment of tissue-specific genes and found sets of tissue-specific genes where a set of neuronal-specific genes were enriched in genes containing G4, MR, DR and STR at their upstream promoter regions (Supplementary Figure 6b). Altogether, these results demonstrate that promoters are enriched for non-B DNA motifs relative to other regulatory elements and relative to other genic compartments and some non-B DNA motifs are more likely to occur at developmental and neuronal genes. Therefore, the excess of genetic variants at non-B DNA motifs identified earlier could have broad implications on gene regulation and subsequent expression levels across tissues and developmental stages.” In the methods section we added the following: “Gene set enrichment analysis. For each type of non-B DNA motif, we extracted a group of genes that contain a non-B DNA motif within a 200-nt upstream window from their TSS and these were used to perform gene set enrichment analyses. GO analyses were performed using clusterProfiler (Yu et al. 2012), where GO terms with at least 20 genes and gene ratios greater than 0.01 for at least one of the non-B DNA sets were considered. For visualisation purposes, we only displayed a maximum of ten GO terms with the highest gene ratio per non-B DNA set. Finally, we calculated the enrichment of each non-B DNA group across sets of tissue-specific genes using TissueEnrich (Jain and Tuteja 2019) using default parameters.

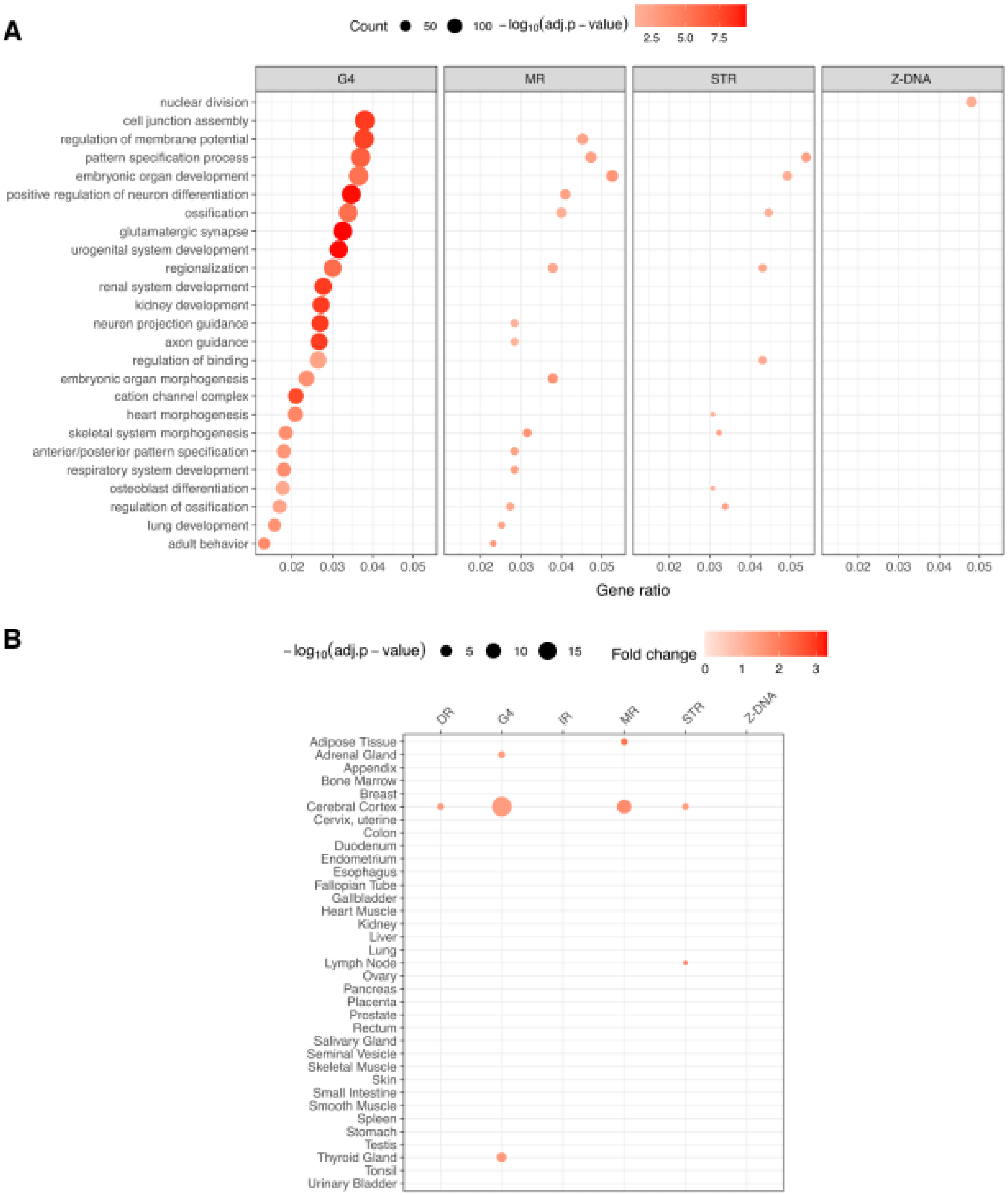

Supplementary Figure 6: Relationship between non-B DNA motifs at promoters, gene categories and tissue expression. a. Gene ontology analysis of non-B DNA motifs found in gene promoters. b. Tissue expression for genes with non-B DNA motifs in their promoters.

7. Figure 4 The authors should provide reference spectra for known G4-DNA and B-DNA. Whereas a clear difference between the stabilizing and non-stabilizing salt conditions is shown, it's not possible to judge whether the stabilizing condition leads to actual G4-DNA conformation without a reference spectrum, particularly to a genomic audience not used to judging DNA spectra.

We have now performed additional experiments to provide more evidence. We have generated a new

supplementary figure that summarizes our findings. 2 known G4-DNA and 1 B-DNA was used as positive and negative control. These serve as reference spectrum in the revised manuscript. We also provide the added supplementary figure below.

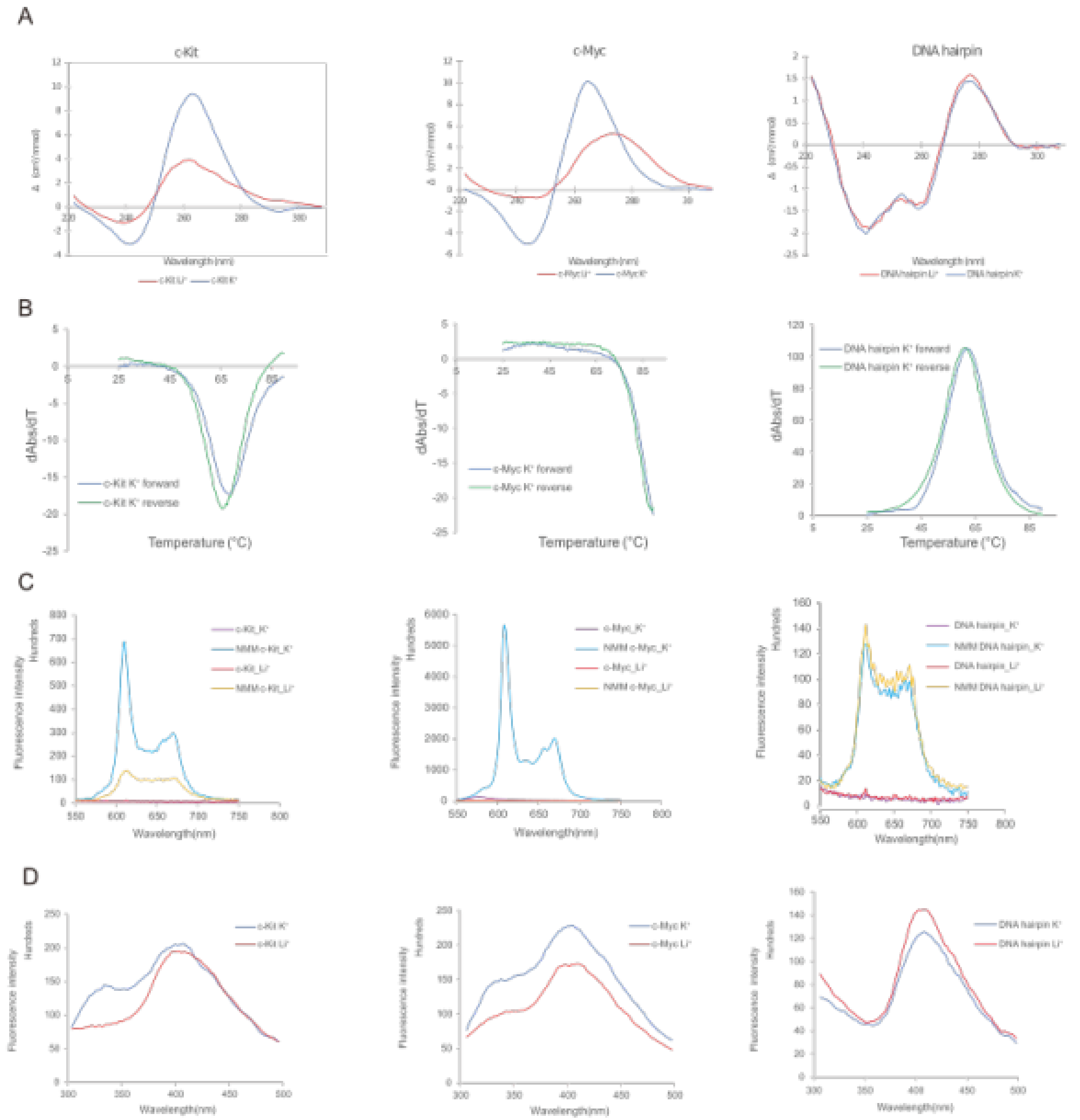

Supplementary Figure 13 Validation experiments performed with positive and negative control sequences. a. Circular dichroism (CD) spectra of the candidate targets for G4 formation potential in presence of two cations. The monovalent ion-dependent nature (G4 stabilized in K+ but not in Li+) indicates the formation of DNA G4s, but not in DNA hairpin, the B-DNA motif. b. UV melting profiles of the G4 candidates in presence of K+ . The reverse melting profile (K+ rev) is also shown and matched well with the forward melting profile (K+). Hyperchromic shift at 295nm is a hallmark for G4 formation, which can be transformed to a negative peak in the derivative plot (dAbs/dT) for G4 stability analysis. The melting temperature (Tm) of a G4 can be identified at the maximum negative value and the B-DNA motif showed a positive value at 260nM instead. c. Fluorescence emission associated with NMM ligand binding to two G4-DNA and one B-DNA candidates in the presence of Li+ or K+ ions. In the absence of NMM ligand, no fluorescence was observed at ~610 nm. Upon NMM addition for two G4-DNAs, weak fluorescence was observed under Li+ , which was substantially enhanced when substituted with K+ ,

supporting the formation of G4 which allows recognition of NMM and enhances its fluorescence. However, upon NMM addition for B-DNA, the fluorescence did not show a significant increase in the presence of Li+ or K+ ions which indicate no G4 formation for B-DNA with NMM. d. Intrinsic fluorescence of two G4- DNA and one B-DNA candidates under Li+ or K+ conditions. The intrinsic fluorescence of G4s was increased when replacing Li+ with K+ , highlighting the formation of DNA G4s but not in the B-DNA motif. We have also updated the text and provide below the added sentence: “We also carried out 2 positive G4 controls and a negative B-DNA control to verify our findings above (Supplementary Figure 13).”

5. In Figure 4F, CNOT6 and SERTAD2 do not show differences between conditions. Can the authors comment on this lack of effect?

For the Figure 4F, they are the forward and reverse UV melting of G4-DNA, which normally will show the same melting profile. We have now clarified about the conditions and expectations in the figure legend.

6. Figure 5d shows control and Z-DNA, not disruption like the text and legend indicate. The title of the figure says, "Mutated Z-DNA". Does it refer to "disrupted Z-DNA"? Expression of Z-DNA is higher than the control, is "control" actually the disrupted Z-DNA? Which gene is the sequence from? Only one promoter was tested?

We thank the reviewer for their comment and apologize for the lack of clarity. The sequences analyzed included scrambled Z-DNA motifs and the genes that included them were SNX12 and SRSF6. We have now removed the misleading figure title from the figure and added the genes for which the analysis refers to in the figure legend.

7. Figure 5 naming is incorrect in the text. 5e was skipped.

We corrected this.

8. Unit is missing in figure 5F (bp?).

We corrected this.

9. Check legend of Figure 5, should be Bonferroni correction and there's a missing comma after panels in the last sentence.

We corrected this.

10. The authors should attempt to provide some commentary on the reasons behind the correlations and enrichment between the various non B-DNA motifs and the features analyzed. Can they speculate on mechanisms behind the different enrichments?

We have added the following statements in the discussion in which we speculate on potential mechanisms: “The increased likelihood of mutagenesis at non-B DNA motifs is also consistent with previous analyses of somatic mutations in cancer genomes (Georgakopoulos-Soares et al., 2018). Different mechanisms underlying the higher mutation rate at individual non-B DNA motifs have been previously identified, such as DNA polymerase slippage errors at microsatellites causing deletions (Bacolla et al., 2004), which was also observed in this study.” “In particular, at experimentally identified G4s, the eQTL enrichment was even larger than that observed across G4 motifs (Figure 1j-k), which is likely due to the formation of G4 motifs being more frequent in open chromatin regions/nucleosome-depleted regions (Hänsel-Hertsch et al., 2016).” “One of the mechanisms by which Z-DNA motifs might increase gene expression might be the reduction of nucleosome occupancy which they elicit (Maruyama et al., 2013). The reduction of expression at promoters with G4 motifs could be due to interference with transcription factor or RNA polymerase II binding. In addition, template G4s have a more inhibitory effect than non-template. The stronger inhibitory effect at the template strand is also aligned with potentially interfering with RNA polymerase II binding. These results are suggestive of inhibitory effects of G4s, which can be mis-characterized if the effect of GC content is not taken into consideration as well as orientation-dependent regulatory effects.”

Referees’ report, second round of review

Reviewer #1: Authors have addressed my concerns

Reviewer #2: The authors thoroughly addressed my comments and concerns, their responses are impressive and insightful. I have no further concerns about this paper.

Authors' response to the second round of review

We thank the reviewers for all their constructive inputs throughout the review process. No further comments from the reviewers were requested at this stage.
